# A Phase 2a randomized, single-center, double-blind, placebo-controlled study to evaluate the safety and preliminary efficacy of oral iOWH032 against cholera diarrhea in a controlled human infection model

**DOI:** 10.1371/journal.pntd.0009969

**Published:** 2021-11-18

**Authors:** Rahsan Erdem, Gwen Ambler, Mohamed Al-Ibrahim, Katarzyna Fraczek, Steven D. Dong, Christopher Gast, Laina D. Mercer, Michael Raine, Sharon M. Tennant, Wilbur H. Chen, Eugenio L. de Hostos, Robert K. M. Choy

**Affiliations:** 1 PATH, Seattle, Washington, United States of America; 2 Pharmaron, Baltimore, Maryland, United States of America; 3 Center for Vaccine Development and Global Health, School of Medicine, University of Maryland, Baltimore, Maryland, United States of America; Washington University School of Medicine, UNITED STATES

## Abstract

Cholera remains a major cause of infectious diarrhea globally. Despite the increased availability of cholera vaccines, there is still an urgent need for other effective interventions to reduce morbidity and mortality. Furthermore, increased prevalence of antibiotic-resistant *Vibrio cholerae* threatens the use of many drugs commonly used to treat cholera. We developed iOWH032, a synthetic small molecule inhibitor of the cystic fibrosis transmembrane conductance regulator chloride channel, as an antisecretory, host-directed therapeutic for cholera. In the study reported here, we tested iOWH032 in a Phase 2a cholera controlled human infection model. Forty-seven subjects were experimentally infected with *V*. *cholerae* El Tor Inaba strain N16961 in an inpatient setting and randomized to receive 500 mg iOWH032 or placebo by mouth every 8 hours for 3 days to determine the safety and efficacy of the compound as a potential treatment for cholera. We found that iOWH032 was generally safe and achieved a mean (± standard deviation) plasma level of 4,270 ng/mL (±2,170) after 3 days of oral dosing. However, the median (95% confidence interval) diarrheal stool output rate for the iOWH032 group was 25.4 mL/hour (8.9, 58.3), compared to 32.6 mL/hour (15.8, 48.2) for the placebo group, a reduction of 23%, which was not statistically significant. There was also no significant decrease in diarrhea severity and number or frequency of stools associated with iOWH032 treatment. We conclude that iOWH032 does not merit future development for treatment of cholera and offer lessons learned for others developing antisecretory therapeutic candidates that seek to demonstrate proof of principle in a cholera controlled human infection model study.

**Trial registration:** This study is registered with ClinicalTrials.gov as NCT04150250.

## Author summary

Cholera, a disease caused by infection with the bacterium *Vibrio cholerae*, remains a major cause of diarrheal illness and death, particularly in settings with poor sanitation and hygiene. We developed a synthetic chemical, named “iOWH032,” as a potential treatment for cholera, which is administered as oral tablets. The chemical acts by blocking secretions from cells in the intestine, and thereby was expected to prevent fluid loss and dehydration caused by cholera illness. We tested iOWH032 in a clinical study using a cholera human challenge model. Study volunteers were intentionally infected with *V*. *cholerae* in an inpatient clinic setting to better study the effects of iOWH032 on infected individuals. This challenge model had been used previously to test cholera vaccine candidates, but this study represents the first test of a potential cholera treatment using the model. We found that treatment of individuals with iOWH032 was safe, but did not result in a significant reduction of cholera illness, based on several different measurements of diarrheal symptoms and severity. This study demonstrates how human challenge models incorporating a relatively small number of subjects can help support decision-making about potential new therapeutics and other interventions for infectious diseases.

## Introduction

Acute secretory diarrhea caused by infection with the bacterium *Vibrio cholerae* is a major cause of diarrheal disease morbidity and mortality. Cholera is responsible for an estimated 2.9 million cases and 95,000 deaths annually [1]. Despite efforts to improve water, sanitation, and hygiene [2], and vaccine introduction [3], major epidemic outbreaks have recently occurred in low-resource settings, including Haiti [4] and Yemen [5]. Furthermore, cholera remains endemic in other regions, including South Asia and parts of sub-Saharan Africa [6,7,8].

Cholera diarrhea is triggered by the action of cholera toxin, an AB_5_-family toxin secreted by *V*. *cholerae* that is taken up by intestinal epithelial cells, which activates cyclic adenosine monophosphate signaling and thereby stimulates activity of the cystic fibrosis transmembrane conductance regulator (CFTR) chloride channel [9]. Activation of CFTR results in hypersecretion of chloride and water into the intestinal tract, leading to rapid, severely dehydrating diarrhea, and up to 50% mortality if untreated [10]. Appropriate treatment of cholera, including oral rehydration therapy, is highly effective and can reduce mortality to <1% [11].

Current therapeutics for cholera commonly used in low-resource settings include antibiotics such as doxycycline and azithromycin, which have demonstrated efficacy in reducing bacterial shedding and diarrheal stool output [12] but are severely threatened by antibiotic resistance [13,14], as are treatments for many other Gram-negative pathogens [15]. Host-directed antisecretory drugs represent an alternative therapeutic strategy for cholera and have the potential to conserve precious resources such as limited supplies of sterile intravenous fluids, but thus far none have rigorously proven efficacious. The antipsychotic drug chlorpromazine [16,17] and the plant alkaloid berberine [18,19] showed mixed results in clinical studies; however, because of liabilities including sedation (chlorpromazine) and drug-drug interactions due to cytochrome P450 inhibition (berberine), these treatments have not been widely adopted. The antisecretory enkephalinase inhibitor racecadotril was tested in Bangladeshi cholera patients but did not significantly reduce diarrheal stool output [20]. The natural product polyphenolic extract crofelemer was reported to modestly reduce diarrheal stool output [21], but this result has not been confirmed in a peer-reviewed report. Anti-motility agents such as the mu opioid agonist loperamide are contraindicated for cholera, particularly in younger children, due to risk of paralytic ileus [22]. A host-directed therapeutic should provide an added benefit in addition to antibiotic therapy, thus raising the bar for demonstrating efficacy and for adoption.

The small molecule antisecretory drug candidate iOWH032 is a CFTR chloride channel inhibitor containing an oxadiazole-carboxamide core with a dibromo-hydroxyphenyl pharmacophore [23]. It was developed for the treatment of cholera and other secretory diarrheas mediated by CFTR activation, such as enterotoxigenic *Escherichia coli*, and has been manufactured at multikilogram scale with an estimated cost-of-goods that is compatible with distribution through public health care channels to enable widespread access. This compound inhibited CFTR in vitro with a 50% inhibitory concentration (IC_50_) of approximately 5 μM (equivalent to 2,725 ng/mL) on Chinese hamster ovary (CHO) cells expressing human CFTR and on T84 colon carcinoma cells (S1 Text). Furthermore, iOWH032 blocked cholera toxin–induced intestinal secretion by more than 90% in a mouse closed-loop model (S1 Fig and S1 Text) and cholera toxin–induced fecal output by nearly 70% in a cecectomized rat model (S2 Fig and S1 Text). iOWH032 was safe in a standard panel of Good Laboratory Practice–compliant toxicology studies, including repeat dose studies in rats and dogs (S1 Text), with no observed adverse effect levels of 2,000 mg/kg/day and 1,000 mg/kg/day, respectively. In two Phase 1 studies conducted in the United States, iOWH032 was administered to 72 healthy adult volunteers and was found to be generally well tolerated at single doses ranging from 30 mg to 1,000 mg, and when administered for 3 days at doses ranging from 100 mg every 12 hours to 500 mg every 8 hours. In a pharmacokinetics study in Bangladeshi cholera patients, a single 300 mg dose of iOWH032 demonstrated an acceptable safety and pharmacokinetic profile [24]. (See S1 Text for more detail on these two studies.)

The cholera controlled human infection model (CHIM) has been in use since the 1960s [25] and involves the experimental infection of healthy volunteers with fully virulent *V*. *cholerae*. Investigators typically measure quantitative endpoints of cholera diarrheal disease, including stool volume output and proportion of subjects with moderate or severe cholera. The model has been used to test several vaccine candidates, including PXVX0200, a live, oral cholera vaccine that was licensed by the United States Food and Drug Administration for prevention of cholera in travelers based on efficacy in a cholera CHIM study [26]. However, prior to this study, no therapeutic candidates had been tested in the cholera CHIM.

In the study described here, we aimed to demonstrate clinical proof of concept of iOWH032 in a cholera CHIM in healthy adult volunteers. While recognizing that efficacy in a CHIM study with a modest number of subjects may not necessarily predict efficacy in the field with a large number of cholera patients, particularly children living in a cholera-endemic setting, we viewed this study as a critical gating step to justify investment in a Phase 3 field study.

## Methods

### Ethics statement

The study protocol and the informed consent documents and amendments were reviewed and approved by the institutional review board of record, Advarra. Written informed consent was obtained from all subjects. This trial is registered on ClinicalTrials.gov (NCT04150250), where the protocol and statistical analysis plan are publicly posted.

### Study design

**Fig 1:**
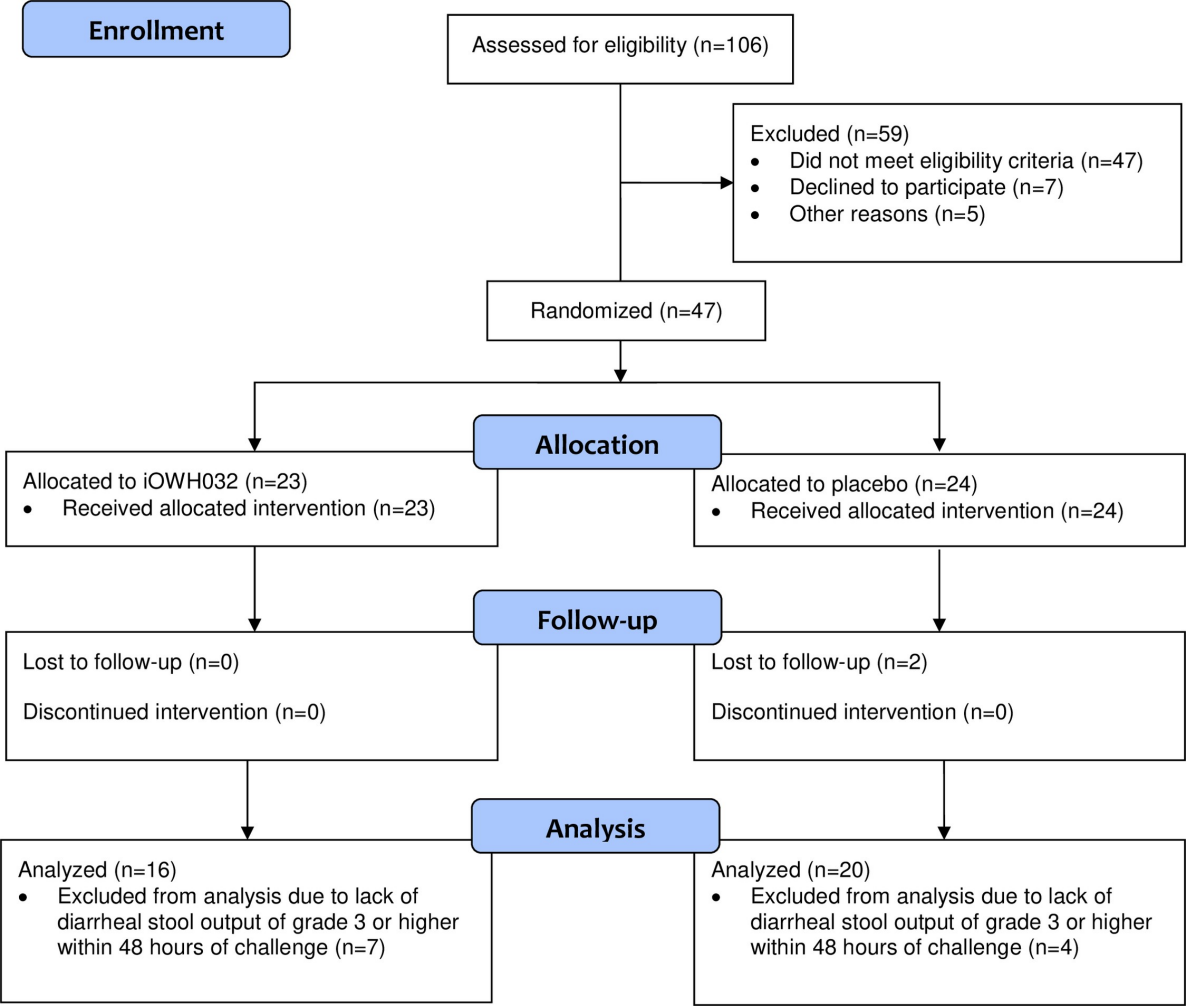
Consolidated Standards for Reporting of Trials flow diagram.

This was a randomized, double-blind, placebo-controlled, parallel, group-sequential Phase 2a study to assess the preliminary clinical efficacy (diarrheal output and clinical symptoms) of oral iOWH032 in a cholera challenge model. The full study protocol is available as S1 Protocol. The study was conducted at a single site in the United States: Pharmaron in Baltimore, Maryland. The study consisted of a screening phase; an inpatient containment period with challenge with *V*. *cholerae* on day 1 followed by treatment with iOWH032 (or placebo); and a post-challenge observation period until discharge, an outpatient follow-up period of at least 28 days, and a final telephone follow-up 6 months post challenge for the collection of severe adverse events (SAEs). The disposition of all subjects from enrollment through allocation, follow-up, and analysis is shown in a diagram that follows the Consolidated Standards for Reporting of Trials (Fig 1).

The study was conducted in two sequential cohorts. The Safety Review Committee reviewed the partially blinded interim analysis of safety data and the primary efficacy endpoint after cohort 1 had completed the inpatient phase and recommended proceeding to cohort 2. All participants in either cohort who received the cholera challenge were randomly assigned in a 1:1 ratio to either the iOWH032 group or the matching placebo group. Randomization was stratified by blood type: O versus non-O.

Treatment started at the onset of diarrhea symptoms or 48 hours after cholera challenge, whichever came first. All participants received 500 mg iOWH032 (two 250 mg immediate-release tablets) or two matching placebo tablets approximately every 8 hours for 3 consecutive days per randomized assignment. All participants also received a 3-day course of ciprofloxacin starting at 4 days post challenge, or when the participant met the criterion for severe cholera diarrhea (>5 L of cumulative diarrheal stool), or at an earlier time at the discretion of the investigator. For participants who remained asymptomatic, the first dose of antimicrobial therapy was administered within 1 to 2 hours after the preceding dose of iOWH032 (or placebo).

Except for the unblinded study site research pharmacist responsible for study drug preparation and dispensing, participants, clinical staff, the investigator, and sponsor personnel were blinded to the study treatment allocation.

### Study population

The study population consisted of healthy adults aged 18 to 44 years recruited from the greater Baltimore, Maryland, USA, area, who were eligible based on the inclusion and exclusion criteria. Participants were excluded for clinically significant medical history—including history of cholera infection or vaccination within the past 10 years. Pregnant women, women who were breastfeeding, and women planning to become pregnant while enrolled in the study were not eligible for inclusion.

### Challenge strain

Participants drank 120 mL sodium bicarbonate solution (approximately 1.3% NaHCO_3_) and then 1 minute later were challenged with 10^6^ colony-forming units of freshly harvested wild-type *V*. *cholerae* El Tor Inaba strain N16961 suspended in 30 mL of sodium bicarbonate solution (approximately 1.3% NaHCO_3_). Consistently, this dose has been shown to elicit acute watery diarrhea within 18 to 48 hours of ingestion [26]. Participants fasted for 90 minutes before and after ingestion of the challenge inoculum.

### Study treatment

The iOWH032 drug product is a chemically and physically stable immediate-release oral 250 mg tablet with a disintegration time of less than 5 minutes. Each tablet contained 40% iOWH032 (w/w) and 60% excipients (w/w), including Pearlitol 160 C, Avicel PH-102, Kollidon CL-F, Kollidon 30, Aerosil 200 Pharma, and magnesium stearate. The matching placebo contained the same excipients, but not the active drug substance.

In previous studies of iOWH032, the highest maximum plasma concentration (C_max_) value among the regimens tested was observed in the group that received 500 mg every 8 hours. The safety profile was similar in all doses evaluated. Accordingly, a dose of 500 mg every 8 hours for 3 days was selected for this trial.

### Clinical management

A full physical examination was performed at screening. At other study visits, a focused physical examination was performed based on clinically relevant issues, symptoms, and medical history. Vital signs (blood pressure and pulse rate) and oral temperature were measured before the challenge and then approximately every 8 hours, or more frequently if indicated, while participants stayed in the inpatient Clinical Research Unit. Vital signs were measured roughly every 4 hours while participants passed diarrheal stool(s) grades through 5 and while any participant sustained a fever. Orthostatic blood pressure was assessed in the event a participant complained of lightheadedness or dizziness upon standing.

Anticipated dehydration was managed with fluid rehydration through administration of standard oral rehydration solution and/or intravenous fluids, based on volume of fluid loss, symptoms indicative of dehydration, and investigator’s discretion. Serum electrolytes, blood urea nitrogen, and creatinine were measured if intravenous fluids were required.

Laboratory adverse events were followed closely until resolution or until a clinically stable endpoint was reached. Pregnancy testing and a 12-lead electrocardiogram were performed for screening and enrollment purposes only.

Participants were discharged from the Clinical Research Unit on day 10 or sooner if they had (1) three consecutive stool cultures, each at least 12 hours apart, negative for growth of *V*. *cholerae*; (2) absence of moderate- or higher-grade objective reactogenicity (diarrhea, fever, and vomiting) for at least 12 hours prior to discharge; and (3) completed a 3-day course of antimicrobial therapy with ciprofloxacin 500 mg twice daily.

### Assessment of efficacy and safety

The maximum temperature, total diarrheal stool volume, number of diarrheal stools, total vomitus volume, and number of vomiting episodes were calculated for the first 7 days. Grading of stool consistency and diarrhea severity was based on a protocol-specified toxicity scale. Diarrheal stool output rate was defined as the total volume of diarrheal stools (as defined in Table 1) divided by the number of hours between the start of study drug administration and the initiation of antimicrobial therapy. A maximum of two stool samples or rectal swabs were collected and examined daily for the presence of *V*. *cholerae* prior to the start of antimicrobial therapy. Quantitative cultures were performed by inoculating stool specimens directly onto thiosulfate citrate bile salts sucrose (TCBS) agar plates. Qualitative cultures were performed by overnight incubation enrichment in alkaline peptone water and then plating on TCBS agar. Suspected *V*. *cholerae* colonies were agglutinated with polyvalent anti-O1 antisera. Blood samples for pharmacokinetic analysis were collected 7±1 hours after the first and last doses of iOWH032. Because of practical limitations on the number of blood samples that could be collected from subjects with cholera at risk for dehydration, we collected blood at this single time point, which represented a trough level.

**Table 1:** Grading of stool consistency and diarrhea severity.

**Normal stool**			**Loose or diarrheal stool**			
Grade 1	Grade 2		Grade 3	Grade 4		Grade 5
Well formed; does not take the shape of the container	Soft; does not easily take the shape of the container		Thick liquid stool; easily takes the shape of the container	Opaque watery diarrheal stool		Clear watery or “rice water” diarrheal stool
**Diarrhea severity**						
Mild		Moderate			Severe	
2 or more loose stools >200 mL^a^ or a single loose stool >300 mL^a^		Cumulative loose stools of 3–5 L^a^			Cumulative loose stools >5 L^a^	

^a^ Onset within 48 hours.

Treatment-emergent adverse events (TEAEs) were defined as adverse events that started or worsened following the start of study medication and up until the follow-up visit. Unsolicited adverse events were collected through 28 days post challenge, and SAEs were collected throughout the study.

### Laboratory methods

Clinical screening and laboratory safety tests were conducted in real-time by the site accredited laboratory. Testing included biochemistry assays for sodium, potassium, creatinine, alkaline phosphatase, total bilirubin, alanine aminotransferase, albumin, aspartate aminotransferase, creatinine phosphokinase, and glucose; whole blood count with differential; hepatitis and HIV serology; blood typing; and pregnancy testing.

For analysis of iOWH032 plasma levels, collected blood specimens were processed into plasma in dipotassium ethylenediaminetetraacetic acid, extracted in acetonitrile, and analyzed using a validated liquid chromatography/mass spectrometry-mass spectrometry assay. Concentration-time data were summarized by treatment group using descriptive statistics at each time point (number, arithmetic mean, geometric mean, median, arithmetic standard deviation, minimum, maximum, and arithmetic coefficient of variation [in %]). A concentration value reported as being “below the lower level of quantification (<LLOQ)” was considered to be zero.

### Statistical methods

#### Sample size calculations

Data from placebo-vaccinated participants (including both type O and non-type O) in a previous cholera challenge study conducted at the University of Maryland, USA, demonstrated a roughly uniform distribution of diarrheal output volumes between 100 mL/day (for both groups) and 2,200 mL/day (non-type O) or 3,000 mL/day (type O) [27]. For sample size computation, it was assumed that in the present study (1) the placebo group would yield daily diarrheal stool rates uniformly distributed between 100 and 3,000 mL/day for type O participants, and between 100 and 2,200 mL/day for non-type O participants; (2) treatment with iOWH032 would yield approximately a 50% reduction in daily diarrheal stool rate, with values uniformly distributed between 100 mL/day and half the upper limit for the placebo group (i.e., 1,500 mL/day and 1,100 mL/day for type O and non-type O participants, respectively); and (3) approximately 40% of participants would have blood type O, and would be equally distributed across study groups and study stage. The estimate of a 50% reduction in stool output was based on preclinical studies in which iOWH032 reduced cholera toxin–induced intestinal secretion by more than 90% in a mouse closed–loop model (S1 Fig and S1 Text) and fecal output by nearly 70% in a cecectomized rat model (S2 Fig and S1 Text). However, the predictive value of these models to human clinical studies has not been established. The estimated efficacy was also based on a reduction that was expected to be clinically meaningful, based on discussions with clinicians experienced in treating cholera in endemic settings.

The primary efficacy endpoint of diarrheal stool output rate was analyzed at both an interim and a final analysis. As the distribution of diarrheal stool output rate is not well characterized and the randomization is stratified by blood type group (O versus non-O), the stratified analog of the Wilcoxon rank-sum test, the Van Elteren test, was employed for joint analysis across blood type groups. Because of the multiplicity of testing and accompanying efficacy and futility thresholds at the interim analysis, a group-sequential framework was employed. Due to the anticipated distribution of the endpoint and the use of stratified nonparametric testing for its evaluation, a simulation-based framework was used with a constrained optimization routine to select boundary values that ensured overall ≥90% power and Type I error ≤0.025, simultaneously.

Due to the non-zero probability of concluding efficacy at the interim analysis, the Type I error rate (α level) for the final analysis was adjusted to maintain the overall 1-sided level of α = 0.025. The threshold to define success on the primary efficacy endpoint at the final analysis was a 1-sided level of α = 0.0238. This hypothesis test was supplemented with a 2-sided 95% confidence interval (CI) for difference in median diarrheal stool output rate, using the percentile bootstrap method (n = 10,000 replicates).

For the secondary endpoint of proportion of participants with moderate (3 to 5 L) or severe (>5 L) diarrhea, with 24 participants per group and assuming 40% of participants would have blood type O, adequate power was available to detect odds ratios of moderate or severe diarrhea in iOWH032-treated participants versus placebo, which were significantly less than 1, using a Cochran-Mantel-Haenszel test stratified by blood type, with a 2-sided level of α = 0.05. A single analysis was conducted for the secondary efficacy endpoints, upon collection of data from all participants included in the study.

#### Analysis of endpoints

The principal analysis was performed using the modified intent-to-treat (mITT) population of those who received at least one dose of study treatment who also displayed evidence of cholera infection within 48 hours of challenge. If >20% of subjects were excluded from the mITT population due to onset of symptoms after 48 hours, the primary endpoint was calculated again including patients with symptom onset after 48 hours. Supportive analysis was conducted using the per-protocol population, defined as a subset of the mITT population that had no major protocol deviations and received all doses of assigned study drug. All safety analyses and summaries were based on the safety population, including all participants who received any study treatment.

The boundary values that were used at the interim analysis were (1) if the 1-sided Van Elteren test of the superiority of the diarrheal stool output rate (iOWH032 versus placebo) yielded a p-value <0.0051, then the study drug was deemed superior to placebo and the second cohort was not enrolled; (2) if the 1-sided Van Elteren test of the superiority of the diarrheal stool output rate (iOWH032 versus placebo) yielded a p-value >0.4585, this was considered to be evidence of futility (lack of demonstrated efficacy) and the second cohort was not enrolled; and (3) in all other cases, the second cohort was enrolled.

All analyses were performed by using Statistical Analysis System version 9.4. Continuous variables were summarized using descriptive statistics. Categorical variables were summarized by frequencies and percentages. Unless otherwise specified, inference such as confidence interval construction was conducted using a 2-tailed Type I error level of α = 0.05. No adjustment for multiple comparisons across endpoints was conducted. All secondary efficacy endpoints were considered as supportive evidence and analyzed without any procedures, to account for multiple comparisons. No algorithm for missing data imputation was employed.

The Van Elteren test was employed for joint analysis across blood type groups. Nonparametric analyses or exact methods (e.g., Fisher’s exact test) were used for efficacy analyses, with confidence intervals for binary variables computed via the Clopper-Pearson exact method, and confidence intervals for continuous variables computed via the percentile bootstrap method, using n = 10,000 replicates each.

## Results

### Demographics

The demographics of the study population are listed in Table 2. There were no significant differences in these characteristics at baseline between treatment groups. Subjects were randomized to ensure approximately equal distribution of O and non-O blood types between treatment groups.

**Table 2:** Demographics and baseline characteristics of the safety population.

Variable	Statistic/Category	Treatment group	
		iOWH032 (N = 23)	Placebo (N = 24)
Age at consent (years)	N	23	24
	Mean (SD)	32.0 (6.15)	32.3 (5.97)
	Median (min, max)	33.0 (21, 44)	32.5 (23, 42)
Sex, n (%)	Male	14 (60.9%)	13 (54.2%)
	Female	9 (39.1%)	11 (45.8%)
Blood type, n (%)	O POS	10 (43.5%)	11 (45.8%)
	O NEG	2 (8.7%)	2 (8.3%)
	A POS	5 (21.7%)	8 (33.3%)
	A NEG	1 (4.3%)	0
	B POS	3 (13.0%)	2 (8.3%)
	B NEG	1 (4.3%)	0
	AB POS	1 (4.3%)	1 (4.2%)
	AB NEG	0	0
	Other	0	0
Blood type status, n (%)	O	12 (52.2%)	13 (54.2%)
	Non-type O	11 (47.8%)	11 (45.8%)
Race, n (%)	White	2 (8.7%)	5 (20.8%)
	Black or African American	21 (91.3%)	19 (79.2%)
Height (cm)	N	23	24
	Mean (SD)	171.5 (6.55)	170.9 (10.84)
	Median (min, max)	170.4 (162, 186)	171.2 (152, 191)
Weight (kg)	N	23	24
	Mean (SD)	84.29 (16.861)	84.75 (12.366)
	Median (min, max)	86.10 (57.7, 122.2)	83.25 (57.9, 110.5)
Body mass index (kg/m^2^)	N	23	24
	Mean (SD)	28.71 (5.660)	29.08 (3.884)
	Median (min, max)	28.40 (20.3, 37.4)	30.35 (19.8, 35.5)

Abbreviations: max, maximum; min, minimum; N, number of participants in respective treatment in safety population; n, number of participants with specified category or non-missing values; %, n/N*100; NEG, negative; POS, positive; SD, standard deviation.

### Safety

Only four subjects (17.4%) in the iOWH032 group and three subjects (12.5%) in the placebo group reported a study drug–related TEAE. The most frequently reported study drug–related TEAEs were nausea, abdominal discomfort, and vomiting (Table 3).

**Table 3:** Study drug–related treatment-emergent adverse events by system organ class and preferred term in the safety population.

System organ class Preferred term	iOWH032 (N = 23)		Placebo (N = 24)	
	n (%)	No. of events	n (%)	No. of events
Participants with at least 1 study drug–related TEAE	4 (17.4%)	5	3 (12.5%)	6
**Gastrointestinal disorders**	**3 (13.0%)**	**4**	**2 (8.3%)**	**3**
Nausea	2 (8.7%)	2	1 (4.2%)	1
Abdominal discomfort	2 (8.7%)	2	0	0
Vomiting	0	0	2 (8.3%)	2
**Nervous system disorders**	**1 (4.3%)**	**1**	**0**	**0**
Headache	1 (4.3%)	1	0	0
**General disorders and administration site conditions**	**0**	**0**	**1 (4.2%)**	**1**
Malaise	0	0	1 (4.2%)	1
**Investigations**	**0**	**0**	**1 (4.2%)**	**2**
Alanine aminotransferase increased	0	0	1 (4.2%)	1
Aspartate aminotransferase increased	0	0	1 (4.2%)	1

Abbreviations: N, number of participants in safety population; n, number of participants with event; TEAE, treatment-emergent adverse event.

Adverse events were coded using the Medical Dictionary for Regulatory Activities, version 22.1. Participants with multiple occurrences of adverse events by the same preferred term or within the same system organ class were counted only once under that preferred term or system organ class, respectively.

As many as 18 subjects (78.3%) in the iOWH032 group and 21 subjects (87.5%) in the placebo group reported at least one TEAE, including both study drug-related and those that could not be specifically attributed to the study drug or cholera challenge. The most frequently reported TEAEs were headache, nausea, diarrhea, and pyrexia. All TEAEs reported by more than one participant are listed in S1 Table.

Overall, treatment with 500 mg iOWH032 every 8 hours for 3 consecutive days was considered safe and well tolerated. None of the participants discontinued from the study due to TEAEs and none of the participants died during the study. One participant in the placebo group experienced an SAE of pyelonephritis during the follow-up phase of the study, 8 weeks after discharge from the inpatient unit on day 68 after enrollment. The SAE was of grade 3 severity and the event was considered by the investigator as not related to study treatment.

### Primary clinical efficacy endpoint

**Fig 2:**
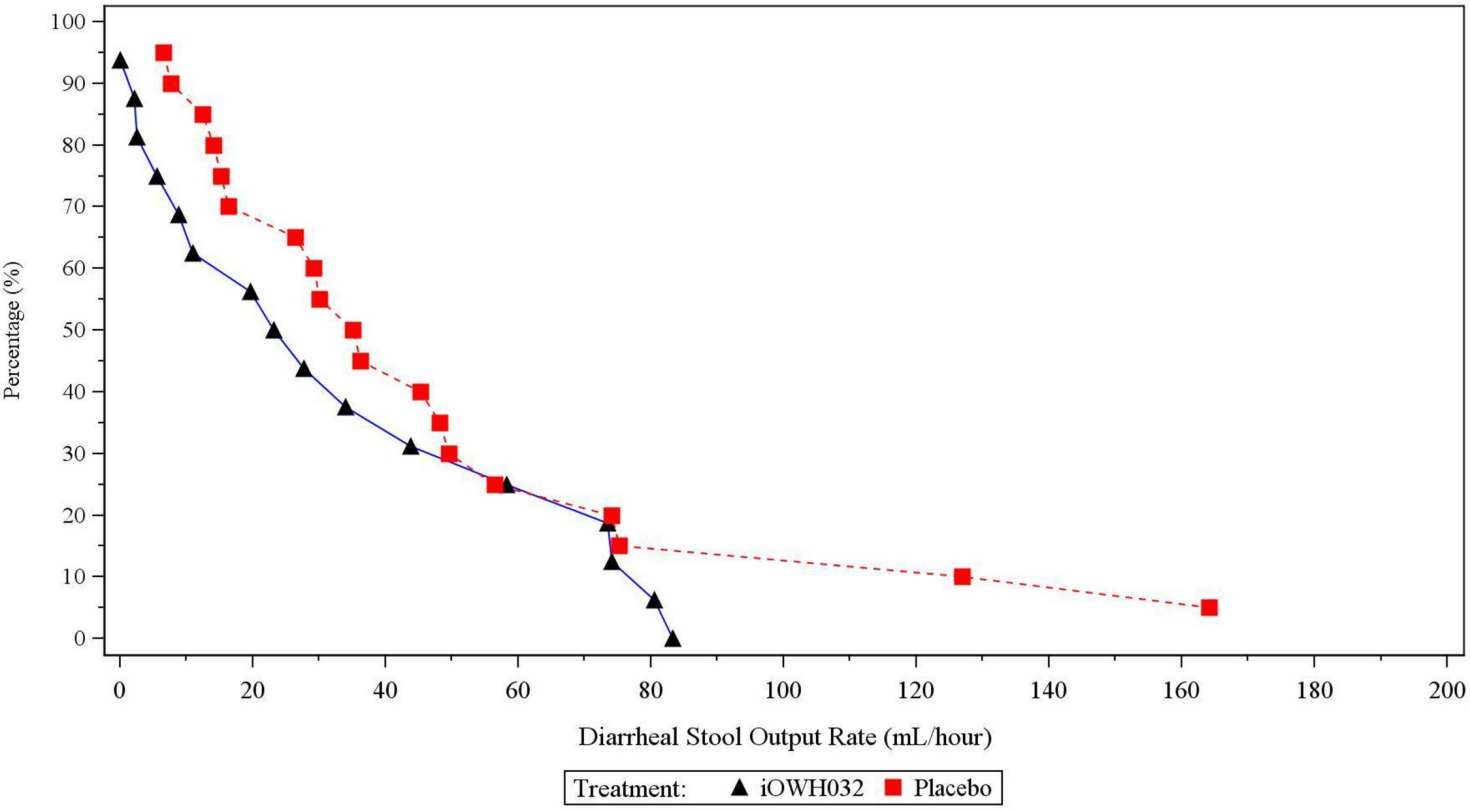
Reverse cumulative distribution plot for diarrheal stool output rate overall in the modified intent-to-treat population. The curve for the iOWH032 is shifted to the left of the placebo group, indicating a lower diarrheal stool output rate; however, this difference was not statistically significant (Van Elteren test: p = 0.2254).

Most of the participants developed diarrhea 18 to 36 hours after the cholera challenge and started the study drug treatment shortly afterward. Three subjects in the iOWH032 treatment group and one subject in the placebo group had no loose stools and were excluded from the efficacy analysis. Furthermore, four additional subjects in the iOWH032 group and three additional subjects in the placebo group had onset of diarrhea more than 48 hours after cholera challenge; these subjects were excluded from the mITT population. A listing of the cumulative diarrhea stool volume for all subjects is shown in S2 Table. For the mITT population, the median (95% CI) diarrheal stool output rate was 25.4 mL/hour (8.9, 58.3) for the 16 participants in the iOWH032 group and 32.6 mL/hour (15.8, 48.2) for the 20 participants in the placebo group, corresponding to a 23% reduction in the iOWH032 group (Table 4). This difference was not statistically significant (Van Elteren test: p = 0.2254). A reverse-cumulative distribution plot is shown in Fig 2. For participants with blood type status O, median diarrheal stool output was similar between the iOWH032 group (30.8 mL/hour) and the placebo group (32.1 mL/hour), whereas for participants with blood type status non-O, median diarrheal stool output tended to be lower in the iOWH032 group (17.1 mL/hour) compared to the placebo group (39.8 mL/hour) (Table 4). However, the study was not adequately powered to determine if these differences were statistically significant. When including participants with symptom onset after 48 hours, the median of diarrheal stool output rate (95% CI) was 25.4 mL/hour (7.8, 51.0) for participants in the iOWH032 group and 29.2 mL/hour (14.1, 45.3) for participants in the placebo group, corresponding to a 13% reduction in the iOWH032 group, also not statistically significant (S3 Table).

**Table 4:** Diarrheal stool output rate overall and by blood type status in the modified intent-to-treat population.

Blood type status Diarrheal stool output rate (mL/hour)	Treatment group	
	iOWH032 (N = 16)	Placebo (N = 20)
**Overall**		
N	16	20
Mean (SD)	34.26 (30.486)	43.47 (41.284)
Median (Q1, Q3)	25.42 (7.2, 65.9)	32.57 (14.7, 53.0)
Min, Max	0.0, 83.3	0.0, 164.2
**Type O status**		
N	8	10
Mean (SD)	38.06 (30.474)	35.40 (36.108)
Median (Q1, Q3)	30.83 (12.6, 65.9)	32.13 (12.5, 45.3)
Min, Max	2.6, 83.3	0.0, 126.9
**Non-type O status**		
N	8	10
Mean (SD)	30.45 (32.091)	51.53 (46.366)
Median (Q1, Q3)	17.09 (5.5, 58.9)	39.84 (16.4, 74.1)
Min, Max	0.0, 80.5	7.7, 164.2

Abbreviations: Max, maximum; Min, minimum; N, number of participants in respective treatment in modified intent-to-treat population; Q1, first quartile; Q3, third quartile; SD, standard deviation.

Diarrheal stool output rate was defined as the total volume of diarrheal stools (mL, grade 3 and higher) divided by the number of hours between initiation of study product dosing and initiation of antimicrobial therapy.

Modified intent-to-treat (mITT) is the subset of the intent-to-treat population that received at least one dose of the study drug. Any participant displaying no indication of cholera infection (no diarrheal stool output of grade 3 or higher) within 48 hours of challenge was removed from the mITT population, prior to unblinding of data.

### Secondary and exploratory efficacy endpoints

One of the key secondary endpoints was a reduction in moderate-to-severe diarrheal disease severity (more than 3 L diarrheal stool output). The proportion (95% CI) of participants in the mITT population with moderate or severe diarrhea following cholera challenge was 43.8% (19.8, 70.1) in the iOWH032 group and 55% (31.5, 76.9) in the placebo group (Table 5). The difference between the treatment groups was not statistically significant (Cochran-Mantel-Haenszel test: p = 0.5145). There was also no statistically significant difference in the proportion of subjects with severe diarrhea (more than 5 L diarrheal stool output) (Table 5). No notable differences in severity of diarrhea between the treatment groups were observed based on blood group status of the participants.

**Table 5:** Diarrheal disease severity by treatment group for the modified intent-to-treat population.

Diarrhea severity	Treatment group	
	iOWH032 (N = 16) n (%)	Placebo (N = 20) n (%)
Mild	9 (56.3%)	9 (45.0%)
Moderate	2 (12.5%)	7 (35.0%)
Severe	5 (31.3)	4 (20.0%)

Several other secondary and exploratory clinical efficacy endpoints were evaluated in this study and are summarized in Table 6. There were no statistically significant differences between treatment groups for median area under the curve of diarrheal stool volume, time to first formed stool, or number of loose (grades 3 through 5) stools. In addition, there were no statistically significant differences between the occurrence of fever, vomiting, or the need for oral rehydration solution and/or intravenous fluid replacement therapy between treatment groups during the study. One participant (6.25%; 95% CI 0.2–30%) in the iOWH032 group and four (20%; 95% CI 5.7–43.7%) in the placebo group received intravenous fluids.

**Table 6:** Secondary efficacy endpoints for the modified intent-to-treat population.

Endpoint	Treatment group		p-value
	iOWH032 (N = 16)	Placebo (N = 20)	
Diarrheal stool volume AUC in liters•hours, median (95% CI)	14.9 (9.3, 20.0)	13.8 (10.0, 16.9)	0.5992 (Van Elteren test)
Time to first formed stool in hours, median (95% CI)	156.5 (114.1, 193.0)	169.7 (108.9, 179.7)	0.6527^a^ (log-rank test)
Number of loose (grades 3–5) stools, median (95% CI)	12.0 (5.0, 15.0)	10.5 (8.0, 16.0)	0.5377 (Van Elteren test)

Abbreviations: AUC, area under the curve; CI, confidence interval.

^a^ For the time to first formed stool analysis, N = 9 for the iOWH032 group and N = 10 for the placebo group because 7 subjects in the iOWH032 group and 10 in the placebo group did not meet the formed stool condition and were excluded from this analysis.

In terms of microbiological endpoints, the median time to cessation of detectable cholera organisms in stool was 6.8 hours longer for the iOWH032 treatment group as compared to placebo, a difference that was statistically significant (S4 Table). More participants in the placebo group received early antibiotics as compared to the iOWH032 group (four versus one). When these five subjects are removed, the difference is reduced to 1.3 hours and is no longer statistically significant. There were no statistically significant differences between treatment groups in median area under the curve or peak shedding of cholera organisms (S4 Table). In addition, there were no notable differences in these parameters between treatment groups based on blood group status.

### Pharmacokinetics

**Fig 3:**
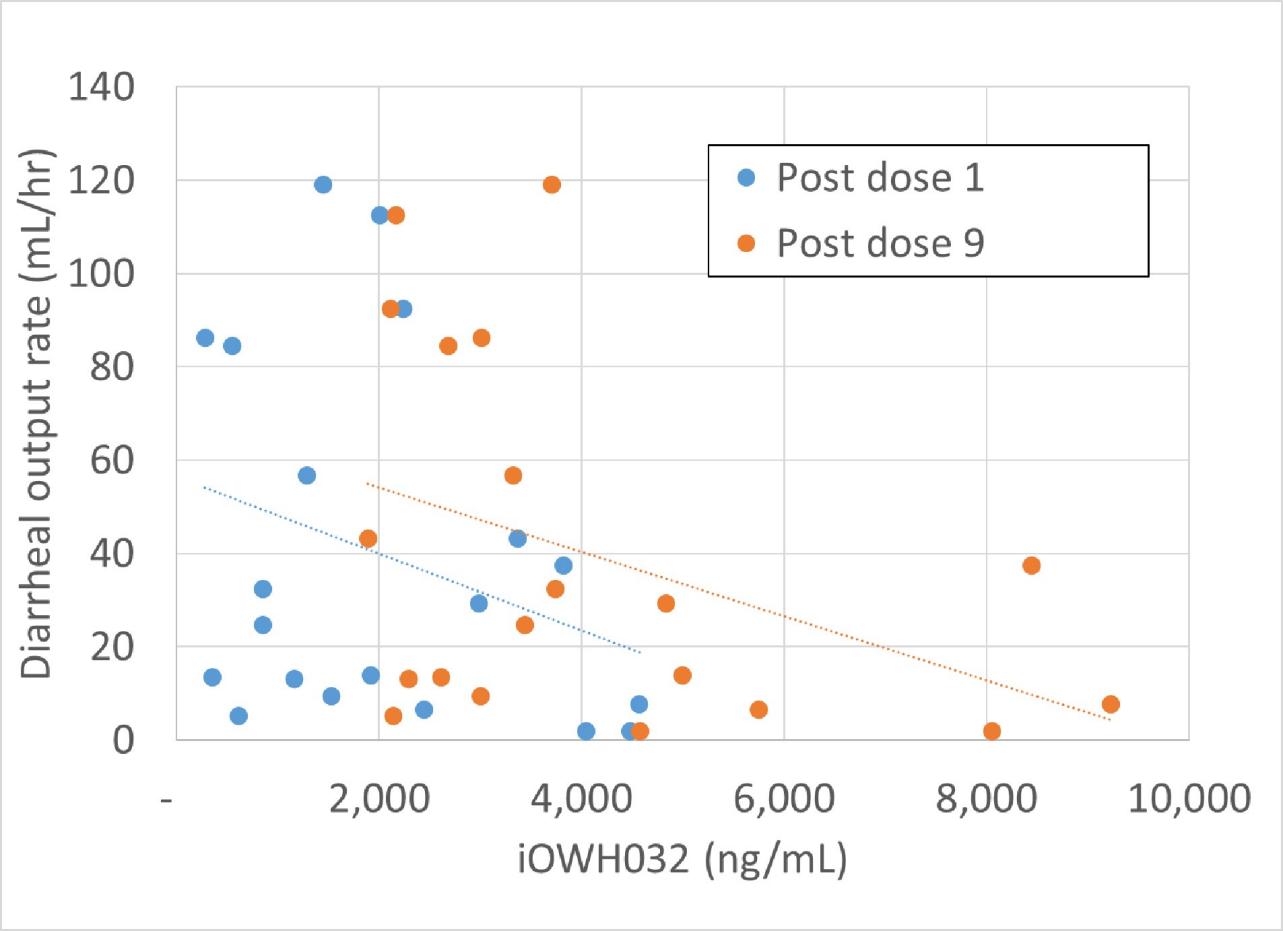
Scatterplot of iOWH032 plasma concentrations versus diarrheal stool output rate. Blue dots: plasma levels at 7 hours after dose 1; orange dots: plasma levels at 7 hours after dose 9. Dotted lines: linear regression plots. The Pearson correlation coefficients for these lines are –0.2997 for post dose 1 data and –0.3937 for post dose 9 data.

For all participants in the iOWH032 group, plasma levels of iOWH032 exceeded the limit of quantitation of 1 ng/mL at both time points sampled. Mean (± standard deviation) iOWH032 plasma levels were 2,250 ng/mL (±1,440) 7±1 hours post dose 1 and 4,270 ng/mL (±2,170) 7±1 hours post dose 9, with median (interquartile range) levels of 2,010 ng/mL (1,006; 3,595) and 3,700 ng/mL (2,645; 4,910), respectively, indicating that iOWH032 plasma concentrations generally increased after 3-day dosing. There was a weak negative correlation between plasma concentrations and diarrheal stool volume output rate (Fig 3); the Pearson correlation coefficients were –0.2997 post dose 1 and –0.3937 post dose 9.

## Discussion

While this study found that administration of 500 mg iOWH032 every 8 hours was safe and resulted in substantial plasma levels of the test compound, we did not observe a significant reduction in the primary efficacy endpoint of stool volume output rate. Furthermore, we did not observe significant effects on any of the secondary efficacy endpoints, such as duration of diarrheal episode, number of diarrheal stools, or diarrheal disease severity. One possibility is that lumenal concentrations of iOWH032 did not reach levels sufficient to inhibit the activation of CFTR by cholera toxin. We did not measure compound concentrations in feces because it is difficult to correlate fecal levels with concentration at the site of action, i.e., CFTR chloride channels on intestinal epithelia. Furthermore, iOWH032 has relatively poor aqueous solubility. This aspect of the compound may be considered as a virtue if it promotes a slow dissolution of compound and spread throughout the intestinal lumen. However, low solubility also means it is difficult to interpret compound levels in feces because they may represent insoluble compound that passed through the entire intestinal tract without the possibility of engaging with the CFTR protein target. An additional complication is that in cases of acute secretory diarrhea, intestinal transit time is greatly reduced [28], thereby reducing the time that compound has for target engagement. Furthermore, compounds may be subject to convective washout forces that reduce concentrations at lumenal targets such as CFTR [29]. The dosing regimen selected for this study was based on the highest dose and frequency tested in a Phase 1 study of healthy volunteers. The target product profile developed at the outset of this project aimed for no more than three times per day dosing to both minimize cost per course of treatment and maximize patient compliance.

Although we did not demonstrate clinical efficacy of iOWH032, this was the first cholera CHIM study to test a therapeutic candidate and there are several important lessons that may be applied to future studies. One relates to the timing of initiation of treatment. We initiated treatment after the first diarrheal stool (grade 3 or higher), which for most patients in our study occurred 18 to 36 hours after cholera challenge. We acknowledge that this regimen may be different from the typical course of treatment for a case of cholera diarrhea in a clinical setting, where most patients do not present for treatment immediately after the first loose stool, but more typically within 2 days after diarrhea onset [30,31]. However, this study was obviously a model rather than a field study, and we selected this dosing regimen based on practicalities of minimizing the total time volunteers would need to be admitted to the in-patient facility, as well as maximizing the amount of time between initiation of test article treatment and when a subject met the case definition for severe cholera, after which they would be required to receive rescue antibiotic therapy according to ethics guidelines.

Another important consideration for the implementation of a cholera CHIM study is the practicality of achieving appropriate statistical power. Because of resource limitations and the maximum capacity of the in-patient clinical ward, we were limited to testing two cohorts of 24 subjects each. This provided 90% power to detect a difference of 50% in the primary efficacy endpoint of diarrheal stool volume rate if all subjects were evaluable, but only 70% power for a 40% reduction or 30% power for a 25% reduction. As a point of comparison, the antisecretory enkephalinase inhibitor racecadotril demonstrated at most a 50% reduction in diarrheal stool volume in children with acute secretory diarrhea [32]. Hypothetically, a study with double the number of subjects (96) would provide the same 90% statistical power to detect a 37% reduction in diarrheal stool volume rate; however, we did not consider this magnitude of reduction to be clinically significant. Although we enrolled and challenged 47 subjects, only 36 were evaluable for the primary endpoint, because roughly 20% of subjects did not meet the case definition for diarrheal disease (at least one loose stool within 48 hours of challenge). Subjects with diarrhea onset after 48 hours were included in many of the analyses because this 20% threshold was exceeded. Future cholera CHIM studies testing therapeutic candidates should carefully consider what magnitude of effect would be clinically significant and the number of subjects needed to obtain statistically significant results.

In a previous Phase 1 pharmacokinetics study in adult Bangladeshi cholera patients who received a single 300 mg dose of iOWH032 [24], the average C_max_ was 482 ± 388 ng/mL (mean ± standard deviation). This represented a 62% decrease compared to healthy adult Bangladeshi volunteers who received the same dose of iOWH032, who had an average C_max_ of 1,275 ± 491 ng/mL. In the study described here, we observed an average concentration of 2,254 ± 1,439 ng/mL 7 hours after the first dose of 500 mg, and an average plasma concentration of 4,266 ± 2,174 ng/mL 7 hours after dose 9. Previous studies indicated the mean (± standard deviation) time to maximum plasma concentration for iOWH032 was 4.8 ± 3.7 hours and the mean (± standard deviation) half-life was 11.5 ± 3.1 hours, suggesting that the time point analyzed in this study was beyond the time to maximum plasma concentration. The reason for this higher compound exposure is unclear, but one possibility is differences in intestinal absorption of compound between cholera patients living in low-income versus high-income country settings.

While we did not observe a reduction in cholera diarrheal stool output with iOWH032 treatment, we established a safe regimen and trough plasma concentrations for which we did not observe any statistically significant increases in treatment-related adverse events. While we do not plan to conduct additional studies to test iOWH032 as a cholera therapeutic, we are exploring applications of this compound for treatment of other disease indications. In contrast to our data that iOWH032 inhibits CFTR, another group reported this compound can paradoxically both inhibit and potentiate human CFTR heterologously expressed in *Xenopus* oocytes, depending on the concentration applied [33]. The basis for these activities and their relevance to in vivo effects is unclear, but these results suggest iOWH032 could have therapeutic benefit for other diseases, including cystic fibrosis [34], influenza [35], and COVID-19 [36].

## Supporting information

S1 Text Supporting information. (DOCX)

S1 Fig iOWH032 inhibits cholera toxin-induced secretion in a mouse closed-loop model. Abbreviations: CTX, cholera toxin; PBS, phosphate buffered saline; DMSO, dimethyl sulfoxide.
(TIF)

S2 Fig iOWH032 inhibits cholera toxin-induced fecal output in a cecectomized rat model. Abbreviations: FOI, fecal output index; PBS, phosphate buffered saline.
(TIF)

S1 Table Treatment-emergent adverse events by preferred term reported in more than one participant (>5%) in any treatment group in the safety population. (DOCX)

S2 Table Cumulative diarrhea stool volume for all subjects. (DOCX)

S3 Table Diarrheal stool output rate overall and by blood type status in the modified intent-to-treat population plus participants with symptom onset after 48 hours. (DOCX)

S4 Table Microbiological endpoints for modified intent-to-treat population. (DOCX)

S1 Protocol Trial protocol. DRG-032-PO-2-01-USA, NCT04150250.
(PDF)

S1 Dataset Source data for Fig 1. (XLSX)

S2 Dataset Source data for Fig 2. (RTF)

S3 Dataset Source data for Fig 3. (XLSX)

S4 Dataset Source data for S1 and S2 Figs. (XLSX)

S1 Checklist CONSORT 2010 checklist. (PDF)
