# Supplementary material for: A Phase 2a randomized, single-center, double-blind, placebo-controlled study to evaluate the safety and preliminary efficacy of oral iOWH032 against cholera diarrhea in a controlled human infection model
Source: PLoS Negl Trop Dis. 2021 Nov 18;15(11):e0009969. doi: 10.1371/journal.pntd.0009969 (PMC8639072; doi:10.1371/journal.pntd.0009969)
Supplement: S1 Text — (DOCX) [file pntd.0009969.s001.docx]

# Supporting information

## Preclinical in vitro and in vivo efficacy studies

Three in vitro assays were used to characterize iOWH032 activity; the fluorescence imaging plate reader (FLIPR) assay, the IonWorks patch clamp assay, and the Ussing chamber short-circuit current assay. The FLIPR assay used T84 colon carcinoma cells labeled with a lipophilic membrane potential-sensitive fluorescent probe. CFTR was activated with forskolin and 3-isobutyl-1-methylxanthine (IBMX) which mimics the effect of cholera toxin by raising intracellular cyclic AMP. In this assay, iOWH032 showed an average IC_50_ of 7.5 μM. In the second assay, a CHO cell line expressing human CFTR was used in a semi-automated patch clamp system (Ion Works Quattro, Molecular Devices Inc.). Chloride currents were again stimulated with forskolin/IBMX, and CFTR inhibition was measured by blocking of this current. The IC_50_ of iOWH032 in this assay was 6.8 μM. In the third in vitro assay, monolayers of T84 cells were mounted in Ussing chambers and stimulated with forskolin/IBMX to activate CFTR. In this assay, the average IC_50_ of iOWH032 was 2.3 μM.

Two rodent assays were used to characterize the ability of iOWH032 to inhibit cholera toxin activation of CFTR in vivo; the mouse closed-loop model (S1 Fig) and the cecectomized rat model (S2 Fig). In the mouse closed-loop model, surgically sealed intestinal loops were injected with cholera toxin with iOWH032 or dimethyl sulfoxide (DMSO) vehicle and intestinal swelling was measured after 4 hours by calculating the weight-to-length ratio of loops. When 100 μg iOWH032 was injected into loops, it decreased cholera toxin-induced swelling by over 90% in this assay. In the second in vivo assay, cecectomized rats were given cholera toxin orally with iOWH032 at 5 mg/kg or DMSO vehicle. A fecal output index (FOI) was calculated by the number and consistency of fecal pellets excreted over an 8 hour period. iOWH032 decreased the FOI by 68% in this study.

## Preclinical toxicology studies in rats and dogs

The toxicology program for iOWH032 was designed to support the three-times-daily oral dose administration of iOWH032 for up to 3 days in clinical trials. The pivotal studies included in the toxicology program to evaluate the safety and support of clinical use were conducted in accordance with US Food and Drug Administration GLP regulations. iOWH032 was well tolerated in all toxicology studies in the rat and dog. There were no major findings in the 14-day GLP toxicity study in rats dosed orally, once daily, with 0, 500, 1000, and 2000 mg/kg. The NOAEL in rats was 2000 mg/kg. In the 14-day GLP toxicity study in dogs dosed orally once a day with 0, 50, 150, and 500 mg/kg, then twice daily (equivalent to 0, 100, 300, and 1000 mg/kg/day), findings were limited to an increase in incidence of slight diarrhea and decrease in body weight gain in the iOWH032-treated dogs. All findings were completely reversible after a two-week recovery period. The NOAEL in dogs was 1000 mg/kg/day. Considering the data summarized above, the FDA’s *Guidance for Industry and Reviewers – Estimating the Safe Starting Dose in Clinical Trials for Therapeutics in Adult Healthy Volunteers* was used to calculate a safe starting dose for the Phase 1 study.

## Phase 1 single-ascending dose and multiple-ascending dose studies

In the single ascending dose study, the most common adverse event was pollakiuria. In the 30 mg dose group, four of six participants who received active drug and none who received placebo reported pollakiuria beginning 3 to 6 hours following receipt of the single dose and persisting for 19 to 30 hours. Three of these same participants also reported micturition urgency, and two reported an increase in stool frequency. All adverse events were rated as mild in severity and resolved spontaneously. Repeated urinalyses were all normal throughout the period of time the participants were symptomatic. Plasma concentrations of iOWH032 in these four participants ranged from approximately 400 to 900 ng/mL. No urinary symptoms were reported by participants enrolled in the 300, 500, or 1,000 mg dose cohorts in the single ascending dose portion of the study. One participant in the food effects portion of the study reported pollakiuria following receipt of the 500 mg dose in both the fed and fasted portions of the study. This participant reported onset of symptoms beginning approximately 4 hours after dosing in each treatment period; and on both occasions, reported being symptomatic for approximately 4 days. As observed in the 30 mg dose cohort, repeated urinalyses were normal and the participant’s symptoms resolved spontaneously. One female participant with no history of cardiovascular disease experienced isolated, asymptomatic premature ventricular contractions at 2 and 3 hours following a single dose of 100 mg of iOWH032.

In the multiple ascending dose study, the most frequently occurring treatment-emergent adverse events (TEAEs) reported in two or more participants overall were application site irritation (at the site of the electrocardiogram patches) (11/40, 27.5%), sinus tachycardia (3/40, 7.5%), catheter site pain (2/40, 5%), decreased appetite (2/40, 5%), and headache (2/40, 5%). Three mild TEAEs of sinus tachycardia were reported by a single participant in each of the following 3-dose cohorts: 300 mg iOWH032 every 8 hours, 500 mg iOWH032 every 12 hours, and 500 mg iOWH032 every 8 hours. Two of these participants had the maximum iOWH032 exposures in their dose cohorts and had close to the maximum overall exposure of all participants during the time of their sinus tachycardia; one of the two participants experienced an accompanying TEAE of increased alertness and the other participant reported no accompanying symptoms. The third participant who had a TEAE of sinus tachycardia reported an accompanying symptom of a “stimulated feeling” and had iOWH032 exposures that approximated the median for his dose cohort and also for all participants. One participant reported a TEAE of palpitations that was unaccompanied by tachycardia. A slight upward trend in average pulse rate change from baseline was observed with increasing iOWH032 plasma concentration, but it was not statistically significant. Mean systolic blood pressure decreased by approximately 10 mmHg in the 300 mg and 500 mg iOWH032 every 8 hours dose cohorts compared to the pooled placebo group, beginning approximately 1 hour following receipt of dose 1 and persisting until approximately 112 hours after dose 1 (approximately 40 hours after receipt of dose 10 of iOWH032).

## Phase 1 pharmacokinetics in cholera patients

In 2013, a two-part bridging pharmacokinetics study was performed in eight healthy adult volunteers (part A) and 12 adult males hospitalized for cholera (part B) at the International Centre for Diarrhoeal Disease Research, Bangladesh. Subjects received a single oral dose of 300 mg iOWH032 in each part of the study, or placebo (part A only), followed by pharmacokinetic sampling over the next 48 hours. In part A, the subjects remained in the clinical trial unit for a minimum of 48 hours post dose; and subjects in part B remained in the hospital for a minimum of 48 hours post dose or 24 hours after resolution of diarrhea, whichever was later. In this bridging pharmacokinetics study, one serious adverse event of elevated serum creatine kinase/creatine phosphokinase was reported in a patient with cholera. This event was deemed unlikely related to the drug, as no clinically relevant features or changes in electrocardiogram parameters were found to be associated.
