# Supplementary figures and images for: A Phase 2a randomized, single-center, double-blind, placebo-controlled study to evaluate the safety and preliminary efficacy of oral iOWH032 against cholera diarrhea in a controlled human infection model
Source: PLoS Negl Trop Dis. 2021 Nov 18;15(11):e0009969. doi: 10.1371/journal.pntd.0009969 (PMC8639072; doi:10.1371/journal.pntd.0009969)

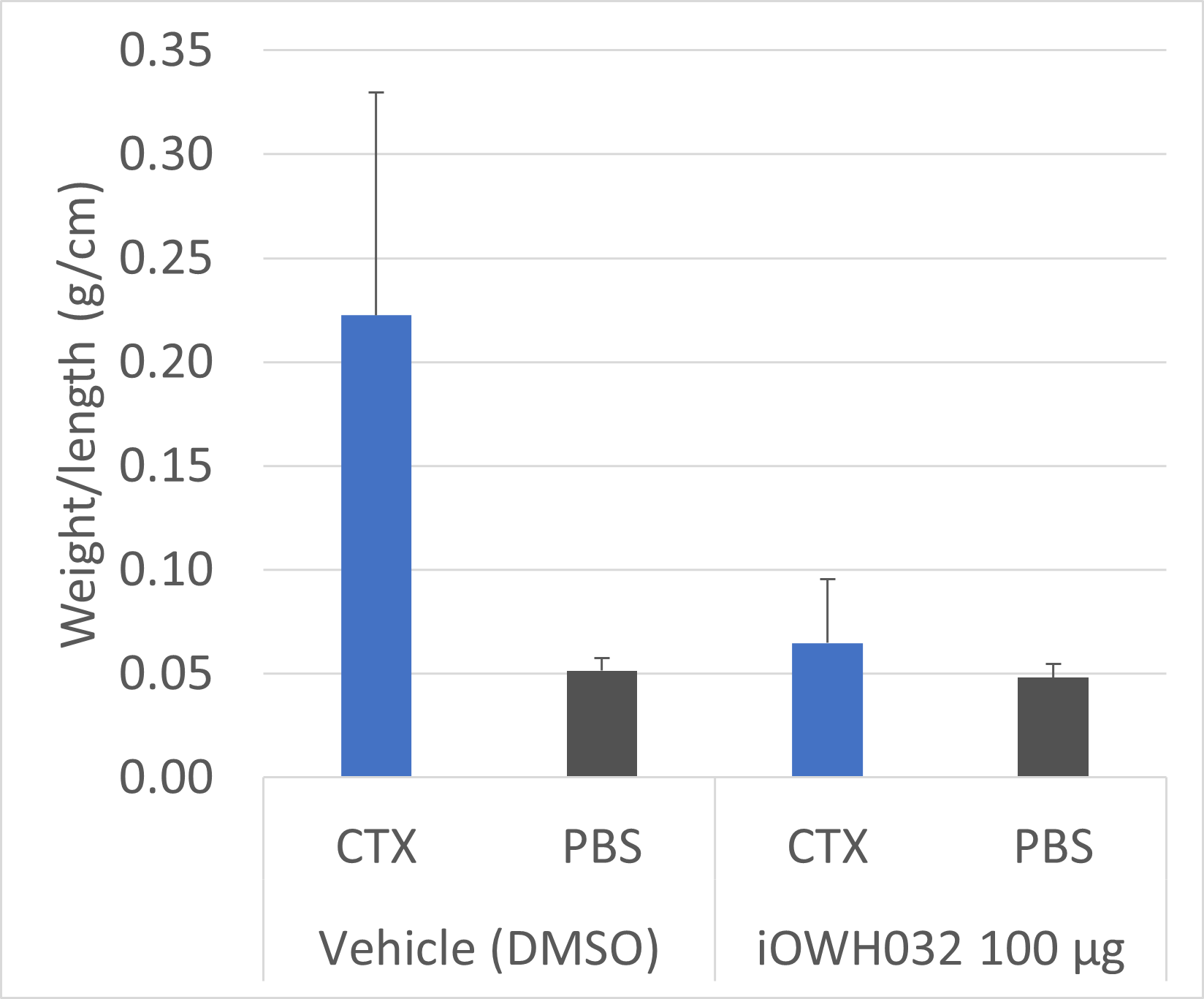

Supplement: S1 Fig — Abbreviations: CTX, cholera toxin; PBS, phosphate buffered saline; DMSO, dimethyl sulfoxide. (TIF) [file pntd.0009969.s002.tif]

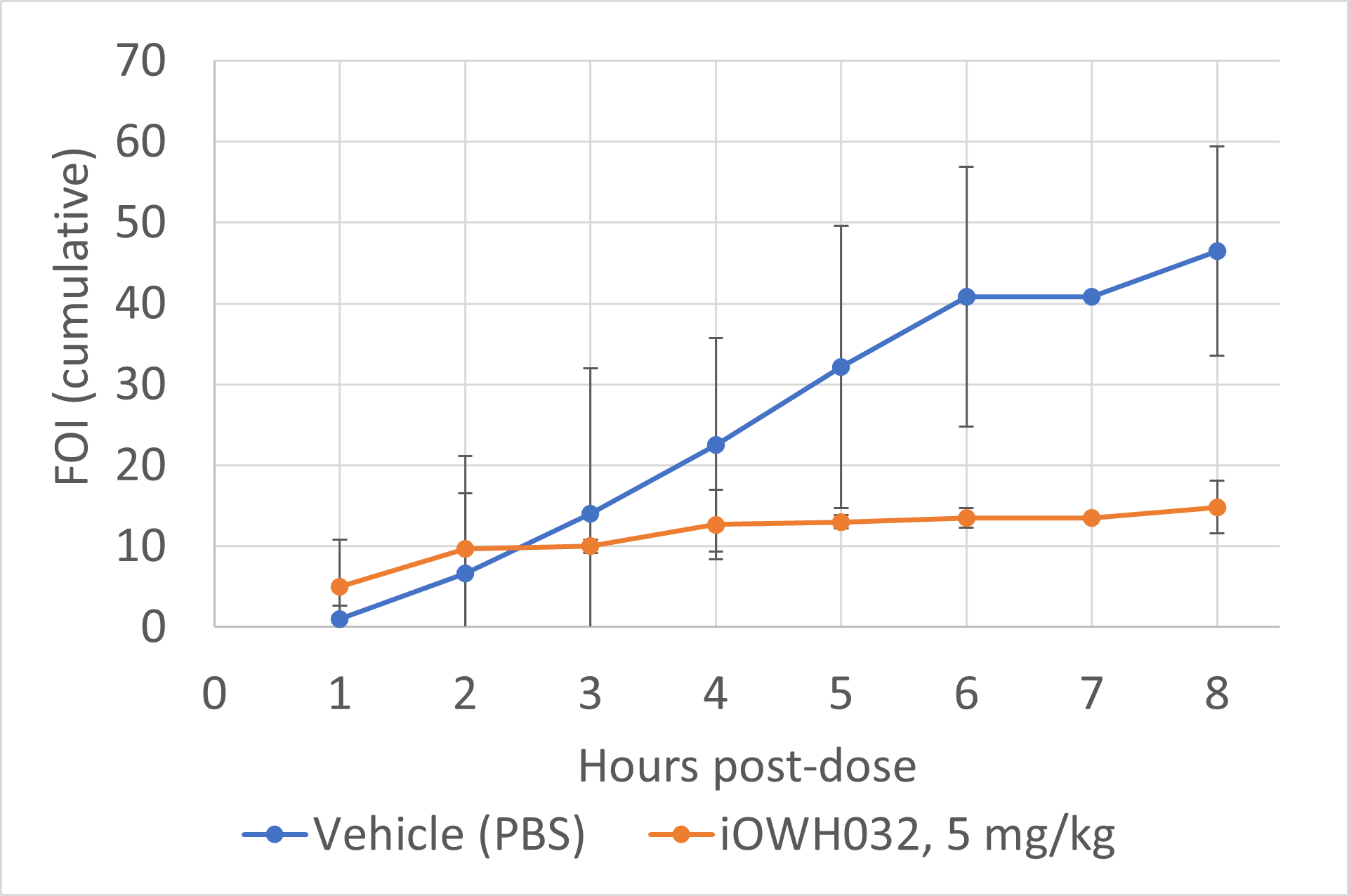

Supplement: S2 Fig — Abbreviations: FOI, fecal output index; PBS, phosphate buffered saline. (TIF) [file pntd.0009969.s003.tif]
