# Supplementary material for: A Phase 2a randomized, single-center, double-blind, placebo-controlled study to evaluate the safety and preliminary efficacy of oral iOWH032 against cholera diarrhea in a controlled human infection model
Source: PLoS Negl Trop Dis. 2021 Nov 18;15(11):e0009969. doi: 10.1371/journal.pntd.0009969 (PMC8639072; doi:10.1371/journal.pntd.0009969)
Supplement: S1 Table — (DOCX) [file pntd.0009969.s004.docx]

**S1 Table. Treatment-emergent adverse events by preferred term reported in more than one participant (>5%) in any treatment group in the safety population.**

| **Preferred term** | **iOWH032 (N=23)** | | **Placebo (N=24)** | |
| --- | --- | --- | --- | --- |
|  | **n (%)** | **No. of events** | **n (%)** | **No. of events** |
| Participants with at least 1 TEAE | 18 (78.3%) | 67 | 21 (87.5%) | 91 |
| Headache | 8 (34.8%) | 8 | 9 (37.5%) | 12 |
| Nausea | 6 (26.1%) | 7 | 5 (20.8%) | 7 |
| Diarrhea | 5 (21.7%) | 5 | 4 (16.7%) | 4 |
| Pyrexia | 4 (17.4%) | 6 | 5 (20.8%) | 5 |
| Sinus tachycardia | 6 (26.1%) | 6 | 1 (4.2%) | 1 |
| Vomiting | 1 (4.3%) | 1 | 6 (25.0%) | 6 |
| Abdominal discomfort | 4 (17.4%) | 5 | 2 (8.3%) | 2 |
| Chills | 4 (17.4%) | 4 | 2 (8.3%) | 2 |
| Decreased appetite | 3 (13.0%) | 3 | 3 (12.5%) | 3 |
| Abdominal pain | 2 (8.7%) | 3 | 4 (16.7%) | 4 |
| Hematochezia | 2 (8.7%) | 2 | 3 (12.5%) | 4 |
| Oropharyngeal pain | 2 (8.7%) | 2 | 2 (8.3%) | 2 |
| Pain | 1 (4.3%) | 1 | 3 (12.5%) | 3 |
| Alanine aminotransferase increased | 0 | 0 | 3 (12.5%) | 3 |
| Back pain | 0 | 0 | 3 (12.5%) | 3 |
| Cough | 0 | 0 | 3 (12.5%) | 3 |
| Aspartate aminotransferase increased | 0 | 0 | 2 (8.3%) | 2 |
| Constipation | 0 | 0 | 2 (8.3%) | 2 |
| Rash | 0 | 0 | 2 (8.3%) | 2 |

Abbreviations: N, number of participants in safety population; n, number of participants with event; TEAE, treatment-emergent adverse event.

Treatment-emergent adverse events were defined as adverse events that started or worsened following the start of study medication and up until the follow-up visit.

Adverse events were coded using the Medical Dictionary for Regulatory Activities, version 22.1. Participants with multiple occurrences of adverse events by the same preferred term were counted only once under that preferred term. Treatment-emergent adverse events are presented in decreasing order of overall frequency.
