# Supplementary material for: A Phase 2a randomized, single-center, double-blind, placebo-controlled study to evaluate the safety and preliminary efficacy of oral iOWH032 against cholera diarrhea in a controlled human infection model
Source: PLoS Negl Trop Dis. 2021 Nov 18;15(11):e0009969. doi: 10.1371/journal.pntd.0009969 (PMC8639072; doi:10.1371/journal.pntd.0009969)
Supplement: S2 Table — (DOCX) [file pntd.0009969.s005.docx]

**S2 Table. Cumulative diarrhea stool volume for all subjects.**

| **Subject number** | **Treatment group** | **Cumulative diarrhea stool volume (mL)** | **Subject number** | **Treatment group** | **Cumulative diarrhea stool volume (mL)** |
| --- | --- | --- | --- | --- | --- |
| 1 | iOWH032 | 7,542 | 24 | Placebo | 12,736 |
| 2 | iOWH032 | 6,832 | 25 | Placebo | 7,656 |
| 3 | iOWH032 | 6,731 | 26 | Placebo | 6,197 |
| 4 | iOWH032 | 6,547 | 27 | Placebo | 5,858 |
| 5 | iOWH032 | 6,042 | 28 | Placebo | 4,557 |
| 6 | iOWH032 | 3,179 | 29 | Placebo | 4,550 |
| 7 | iOWH032 | 3,012 | 30 | Placebo | 4,493 |
| 8 | iOWH032 | 2,823 | 31 | Placebo | 4,375 |
| 9 | iOWH032 | 2,533 | 32 | Placebo | 3,565 |
| 10 | iOWH032 | 1,757 | 33 | Placebo | 3,555 |
| 11 | iOWH032 | 1,407 | 34 | Placebo | 3,273 |
| 12 | iOWH032 | 1,003 | 35 | Placebo | 2,592 |
| 13 | iOWH032 | 1,001 | 36 | Placebo | 2,510 |
| 14 | iOWH032 | 947 | 37 | Placebo | 2,157 |
| 15 | iOWH032 | 566^a^ | 38 | Placebo | 1,586 |
| 16 | iOWH032 | 399 | 39 | Placebo | 1,471 |
| 17 | iOWH032 | 375^a^ | 40 | Placebo | 1,444 |
| 18 | iOWH032 | 324^a^ | 41 | Placebo | 1,358 |
| 19 | iOWH032 | 169 | 42 | Placebo | 906 |
| 20 | iOWH032 | 96 | 43 | Placebo | 724^a^ |
| 21 | iOWH032 | 0^b^ | 44 | Placebo | 532^a^ |
| 22 | iOWH032 | 0^b^ | 45 | Placebo | 483^a^ |
| 23 | iOWH032 | 0^b^ | 46 | Placebo | 341 |
|  |  |  | 47 | Placebo | 0^b^ |

^a^ Excluded from modified intent-to-treat population because of diarrhea onset after 48 hours.

^b^ Excluded from modified intent-to-treat population because of no diarrheal stools.
