# Supplementary material for: A Phase 2a randomized, single-center, double-blind, placebo-controlled study to evaluate the safety and preliminary efficacy of oral iOWH032 against cholera diarrhea in a controlled human infection model
Source: PLoS Negl Trop Dis. 2021 Nov 18;15(11):e0009969. doi: 10.1371/journal.pntd.0009969 (PMC8639072; doi:10.1371/journal.pntd.0009969)
Supplement: S3 Table — (DOCX) [file pntd.0009969.s006.docx]

**S****3 Table. Diarrheal stool output rate overall and by blood type status in the modified intent-to-treat population plus participants with symptom onset after 48 hours.**

| **Blood type status**  Diarrheal stool output rate (mL/hour) | **Treatment group** | |
| --- | --- | --- |
|  | **iOWH032**  **(N=20)** | **Placebo**  **(N=23)** |
| **Overall** |  |  |
| N | 20 | 23 |
| Mean (SD) | 32.99 (29.659) | 39.20 (40.004) |
| Median (Q1, Q3) | 25.42 (6.3, 63.8) | 29.22 (12.5, 49.6) |
| Min, Max | 0.0, 83.3 | 0.0, 164.2 |
| **Type O status** |  |  |
| N | 10 | 12 |
| Mean (SD) | 34.10 (28.654) | 31.64 (33.826) |
| Median (Q1, Q3) | 28.68 (6.8, 58.3) | 21.68 (12.3, 40.8) |
| Min, Max | 2.6, 83.3 | 0.0, 126.9 |
| **Non-type O status** |  |  |
| N | 10 | 11 |
| Mean (SD) | 31.87 (32.144) | 47.45 (46.028) |
| Median (Q1, Q3) | 17.09 (5.8, 69.2) | 30.09 (15.3, 74.1) |
| Min, Max | 0.0, 80.5 | 6.6, 164.2 |

Abbreviations: Max, maximum; Min, minimum; N, number of participants in respective treatment in modified intent-to-treat population and participants with symptom onset after 48 hours; Q1, first quartile; Q3, third quartile; SD, standard deviation.

For the modified intent-to-treat (mITT) population, diarrheal stool output rate was defined as the total volume of diarrheal stools (mL, grade 3 and higher) divided by the number of hours between initiation of study product dosing and initiation of antimicrobial therapy.

For the non-mITT population, diarrheal stool output rate was defined as the total volume of diarrheal stools (mL, grade 3 and higher) divided by the number of hours between onset of symptoms and initiation of antimicrobial therapy. mITT is the subset of the intent-to-treat population that received at least one dose of the study drug. Any participant displaying no indication of cholera infection (no diarrheal stool output of grade 3 or higher) within 48 hours of challenge was removed from the mITT population, prior to unblinding of data.
