# Supplementary material for: A Phase 2a randomized, single-center, double-blind, placebo-controlled study to evaluate the safety and preliminary efficacy of oral iOWH032 against cholera diarrhea in a controlled human infection model
Source: PLoS Negl Trop Dis. 2021 Nov 18;15(11):e0009969. doi: 10.1371/journal.pntd.0009969 (PMC8639072; doi:10.1371/journal.pntd.0009969)
Supplement: S4 Table — (DOCX) [file pntd.0009969.s007.docx]

**S4 Table. Microbiological endpoints for modified intent-to-treat population.**

| **Endpoint** | **Treatment group** | | **p-value** |
| --- | --- | --- | --- |
|  | **iOWH032 (N=16)** | **Placebo (N=20)** |  |
| Median (95% CI) time to cessation of cholera organisms in stool (h) | 109.4 (102.9, 119.2) | 102.6 (98.3, 111.2) | 0.0272  (Kaplan-Meier method) |
| Median (95% CI) AUC shedding of cholera organisms (x10^5^ CFU•h/g) | 6.7 (0.4, 25.1) | 16.5 (6.1, 31.8) | 0.1427  (Van Elteren test) |
| Median (95% CI) peak shedding of cholera organisms (x10^7^ CFU/g) | 6.6 (0.47, 14.2) | 7.3 (3.1, 14.0) | 0.4252  (Van Elteren test) |

Abbreviations: AUC, area under the curve; CFU, colony forming units; CI, confidence interval; g, grams; h, hours.
