# Supplementary material for: A Phase 2a randomized, single-center, double-blind, placebo-controlled study to evaluate the safety and preliminary efficacy of oral iOWH032 against cholera diarrhea in a controlled human infection model
Source: PLoS Negl Trop Dis. 2021 Nov 18;15(11):e0009969. doi: 10.1371/journal.pntd.0009969 (PMC8639072; doi:10.1371/journal.pntd.0009969)
Supplement: S1 Protocol — DRG-032-PO-2-01-USA, NCT04150250. (PDF) [file pntd.0009969.s008.pdf]

Title

A Phase 2a Randomized, Single-Center, Double-Blind, Placebo Controlled Study to Evaluate the Safety and Preliminary Efficacy of Oral iOWH032 Against Cholera Diarrhea in a Controlled Human Infection Model

Protocol Number iOWH032
DRG-032-PO-2-01-USA

Name of Principal Investigator:
[redacted]

National Clinical Trial (NCT) Identifier Number:
NCT04150250

Sponsor:
PATH

Funded by:
[redacted]

Protocol Version Number:
4.0
REDACTED VERSION

Version Date:
13 March 2020

CONFIDENTIAL

Confidentiality Statement

This document is confidential and is to be distributed for review only to investigators, potential investigators, consultants, study staff, applicable independent ethics committees or institutional review boards, and applicable regulatory authorities. The contents of this document shall not be disclosed to others without written authorization from PATH (or others, as applicable), unless it is necessary to obtain informed consent from potential study participants.

TABLE OF CONTENTS

Contents

TABLE OF CONTENTS 2
LIST OF ABBREVIATIONS AND ACRONYMS 7
STATEMENT OF COMPLIANCE 8
PROTOCOL SUMMARY 9
1 STUDY RATIONALE 13
2 KEY ROLES 14
3 INTRODUCTION: BACKGROUND INFORMATION AND SCIENTIFIC RATIONALE 15
3.1 iOWH032 General Properties & Pharmacodynamics 15
3.2 Summary of Nonclinical Studies of Study Drug 16
3.3 Summary of Clinical Studies of iOWH032 16
3.4 Background Information on the Cholera Challenge Model 18
3.5 Potential Risks and Benefits 19
3.5.1 Known Potential Risks 20
3.5.2 Known Potential Benefits 21
3.6 Overall Development Strategy 21
4 HYPOTHESIS, OBJECTIVES AND PURPOSE 21
4.1 Study Hypothesis / Hypotheses 21
4.2 Study Objectives 21
4.2.1 Primary Efficacy Objective 21
4.2.2 Primary Safety Objective: 21
4.2.3 Secondary Efficacy Objectives: 22
4.2.4 Secondary Safety Objective: 22
5 STUDY DESIGN AND ENDPOINTS 22
5.1 Description of the Study Design 22
5.2 Study Endpoints 22
5.2.1 Primary Efficacy Endpoints 22
5.2.1 Primary Safety Endpoint: 22
5.2.2 Secondary Efficacy Endpoints: 22
5.2.3 Secondary Safety Endpoints: 23
6 STUDY ENROLLMENT AND WITHDRAWAL 23
6.1 Participant Inclusion Criteria 23
6.2 Participant Exclusion Criteria 24
6.3 Strategies for Recruitment and Retention 25
6.4 Withdrawal from Study 25
6.4.2 Handling of Participant Withdrawals or Termination 26
6.5 Premature Termination or Suspension of Study 26

7 STUDY AGENTS ... 27
7.1 Study Agent and Control Description ... 27
7.1.1 Product Description ... 27
7.1.2 Manufacturer ... 27
7.1.3 Acquisition ... 27
7.1.4 Formulation, Appearance, Packaging, and Labeling ... 27
7.1.5 Product Storage and Stability ... 27
7.1.6 Preparation ... 28
7.1.7 Dosing and Administration ... 28
7.1.8 Route of Administration ... 28
7.1.9 Starting Dose ... 28
7.1.10 Dose Adjustments/Modifications/Delays ... 28
7.1.11 Duration of Therapy ... 28
7.1.12 Tracking of Dose ... 28
7.2 Challenge Strain Description ... 29
7.2.1 Product Description ... 29
7.2.2 Manufacturer ... 29
7.2.3 Acquisition ... 29
7.2.4 Formulation, Appearance, Packaging, and Labeling ... 29
7.2.5 Product Storage and Stability ... 29
7.2.6 Preparation ... 29
7.2.7 Dosing and Administration ... 29
7.2.8 Route of Administration ... 30
7.2.9 Starting Dose ... 30
7.2.10 Dose Adjustments/Modifications/Delays ... 30
7.2.11 Duration of Therapy ... 30
7.2.12 Tracking of Dose ... 30
7.3 Study Agents Accountability Procedures ... 30
8 STUDY PROCEDURES AND SCHEDULE ... 30
8.1 Study Procedures/Evaluations ... 30
8.1.1 Study Specific Procedures ... 30
8.1.2 Standard of Care Study Procedures ... 31
8.2 Laboratory Procedures/Evaluations ... 31
8.2.1 Clinical Laboratory Evaluations ... 31
8.2.2 Stool Specimen Preparation, Handling, and Storage ... 31
8.2.3 Specimen Shipment ... 31

8.3 Study Schedule 32
8.3.1 Screening (Outpatient Clinic Visit 1) 32
8.3.2 Inpatient Containment Period 33
8.3.3 Acclimatization (1 day prior to challenge, Day -1) 33
8.3.4 Challenge Day (Day 1) 34
8.3.5 Post-Challenge Observation Period (Day 1 through discharge) 34
8.4 Outpatient Clinic Visit 2 (Day 15 ±1) 35
8.5 Outpatient Clinic Visit 3 (Day 29 ±2) 35
8.6 Final Follow-up – Visit 4 (Day 180 ±14) 35
8.7 Unscheduled Visit(s) 35
8.8 Concomitant Medications, Treatments, and Procedures 36
8.9 Prohibited Medications, Treatments, and Procedures 36
9 MANAGEMENT OF EXPECTED CHOLERA ILLNESS 36
9.1 Clinical Evaluation 36
9.2 Measurement of Diarrhea and Vomitus 36
9.3 Management of Fluid Loss 36
9.4 Frequency of Vital Signs Assessment 37
9.5 Fluid Therapy/Hydration 37
9.6 Indications for Antibiotics 37
9.7 Indications for Other Concomitant Medications 38
10 ASSESSMENT OF SAFETY 38
10.1 Specification of Safety Parameters 38
10.1.1 Definition of Adverse Event (AE) 38
10.1.2 Definition of Serious Adverse Event (SAE) 39
10.1.3 Definition of Unanticipated Problems (UP) 40
10.2 Classification of an Adverse Event 40
10.2.1 Severity of Event 40
10.2.2 Relationship to Study Agent or Cholera Infection 40
10.2.3 Expectedness 41
10.3 Time Period and Frequency for Event Assessment and Follow-up 41
10.4 Reporting Procedures 42
10.4.1 Adverse Event Reporting 42
10.4.2 Serious Adverse Event Reporting 42
10.4.3 Unanticipated Problem Reporting 43
10.4.4 Reporting of Pregnancy 43
10.5 Study Halting Rules 44
10.6 Safety Oversight 44
11 CLINICAL MONITORING 45
12 STATISTICAL CONSIDERATIONS 45
12.1 Statistical and Analytical Plans 45
12.2 Statistical Hypotheses 45

12.3 Timing of Analyses ... 46
12.4 Analysis Datasets ... 46
12.5 Description of Statistical Methods ... 47
12.5.1 General Approach ... 47
12.5.2 Analysis of the Primary Efficacy Endpoint(s) ... 48
12.5.3 Analysis of the Secondary Efficacy Endpoints ... 48
12.5.4 Safety Analyses ... 49
12.5.5 Adherence and Retention Analyses ... 50
12.5.6 Protocol Deviations ... 50
12.5.7 Baseline Descriptive Statistics ... 50
12.5.8 Planned Interim Analyses ... 50
12.5.9 Multiple Comparison/Multiplicity ... 51
12.5.10 Exploratory Analyses ... 51
12.5.11 Missing data ... 51
12.6 Sample Size ... 51
12.7 Measures to Minimize Bias ... 53
12.7.1 Enrollment/ Randomization/ Masking Procedures ... 53
12.7.2 Evaluation of Success of Blinding ... 53
12.7.3 Breaking the Study Blind/Participant code ... 53
13 SOURCE DOCUMENTS AND ACCESS TO SOURCE DATA/DOCUMENTS ... 53
14 QUALITY ASSURANCE AND QUALITY CONTROL ... 54
15 ETHICAL CONSIDERATIONS (AND INFORMED CONSENT) ... 54
15.1 Ethical Standard ... 54
15.2 Institutional Review Board ... 54
15.3 Informed Consent Process ... 55
15.3.1 Consent and Other Informational Documents Provided to Participants ... 55
15.3.2 Consent Procedures and Documentation ... 55
15.4 Exclusion of Women, Minorities, and Children (Special Populations) ... 55
15.5 Participant and Data Confidentiality ... 55
15.5.1 Research Use of Stored Human Samples, Specimens, or Data ... 56
15.6 Future Use of Stored Samples ... 56
16 DATA HANDLING AND RECORDKEEPING ... 56
16.1 Data Collection and Data Management Responsibilities ... 56
16.2 Study Records Retention ... 57
16.3 Protocol Deviations ... 57
16.4 Publications and Data Sharing Policy ... 58
17 FINANCING AND INSURANCE ... 58
18 CONFLICT OF INTEREST POLICY ... 58
19 REFERENCES ... 60
APPENDIX A: PROTOCOL REVISIONS ... 62

APPENDIX B: SCREENING TESTS 63
APPENDIX C: SCHEDULE OF STUDY VISITS AND EVALUATIONS........................... 65
APPENDIX D: CLINICAL SAFETY LABORATORY TOXICITY SCALES 67
APPENDIX E: REACTOGENICITY AND SOLICITED ADVERSE EVENT TOXICITY SCALES 68

LIST OF ABBREVIATIONS AND ACRONYMS

AE	Adverse Events
ALT	Alanine Aminotransferase
AST	Aspartate Aminotransferase
CFR	Code of Federal Regulations
CFU	Colony-forming Unit
CPK	Creatine phosphokinase
CRF	Case Report Form
CFTR	Cystic Fibrosis Transmembrane Conductance Regulator
CYP2C9	Cytochrome P450 2C9
[redacted]	[redacted]
ECG	Electrocardiogram
eCRF	Electronic Case Report Form
FDA	Food and Drug Administration
GCP	Good Clinical Practices
HIV	Human Immunodeficiency Virus
ICH	International Council on Harmonisation
IND	Investigational New Drug
IRB	Institutional Review Board
ITT	Intent to Treat
MedDRA	Medical Dictionary for Regulatory Activities
mg	milligram
mITT	Modified Intent to Treat
mL	milliliter
MOP	Manual of Operating Procedures
OHRP	Office of Human Research Protections
PI	Principal Investigator
SAE	Serious Adverse Event
SAD	Single Ascending Dose
SOP	Standard Operating Procedure (s)
SRC	Safety Review Committee
TA	Test Article
TEAE	Treatment-emergent Adverse Events
ULN	Upper Limit of Normal
WHO	World Health Organization

STATEMENT OF COMPLIANCE

The trial will be conducted in accordance with the ICH E6 and the Code of Federal Regulations on the Protection of Human Subjects (45 CFR Part 46). The Principal Investigator will assure that no deviation from, or changes to the protocol will take place without prior agreement from the sponsor and documented approval from the Institutional Review Board (IRB), except where necessary to eliminate an immediate hazard(s) to the trial participants. All personnel involved in the conduct of the study will have completed ICH GCP training, and that all key investigators will have completed HSP and RCR training.

I agree to ensure that all staff members involved in the conduct of this study are informed about their obligations in meeting the above commitments.

Principal Investigator: [redacted]
Print/Type Name

Signed: ____________________________ Date: _______________
Signature

PROTOCOL SUMMARY

**Title**	A Phase 2a Randomized, Single-Center, Double-Blind, Placebo Controlled Study to Evaluate the Safety and Preliminary Efficacy of Oral iOWH032 Against Cholera Diarrhea in a Controlled Human Infection Model
**Short Title**	iOWH032 Efficacy against Cholera
**Protocol Number**	DRG-032-PO-2-01-USA
**IND Number**	110,938 (iOWH032)
**NCT Number**	NCT04150250
**Trial Phase**	Phase 2a
**Study Summary**	This is a single-center, randomized, double-blind, placebo-controlled trial to evaluate the therapeutic efficacy of oral doses of iOWH032 on diarrhea output and clinical symptoms after a cholera challenge. There will be 24 study participants challenged in the first cohort and an additional 24 study participants challenged in the second cohort, pending favorable results of an interim analysis. Participants will be randomized 1:1 to receive either iOWH032 500 mg every 8 hours for three days or matching placebo. Blinded therapeutic dosing will start at the onset of diarrhea or by 48 hours after ingesting the challenge inoculum of 10^6 cfu of *V. cholerae* El Tor Inaba strain N16961. The observation and management of cholera diarrhea and symptomatology will occur on an inpatient isolation research ward over a duration of ~11 days, including a three-day course of antibiotics to treat all participants prior to discharge from the inpatient unit. The final study follow-up visit will be six months after the challenge via phone call.
**Study Hypotheses**	• iOWH032 will demonstrate reduction in fluid output against cholera diarrhea<br>• iOWH032 will be safe and well-tolerated
**Objectives**	**Primary Efficacy Objective:**<br>• To measure the rate and extent of diarrhea following cholera challenge, in participants treated with iOWH032 compared to placebo<br>**Primary Safety Objective:**<br>• To evaluate safety and tolerability of oral doses of iOWH032 compared to placebo<br>**Secondary Efficacy Objectives:**<br>• To evaluate additional measures of efficacy against cholera illness following cholera challenge, among those randomized to be treated with iOWH032 versus placebo<br>**Secondary Safety Objectives:**<br>• To evaluate tolerability of oral doses of iOWH032 compared to placebo<br>**Exploratory Objectives:**<br>• To evaluate the pharmacokinetic (pK) properties of iOWH032<br>• To evaluate time of cessation of cholera organism in stool after challenge<br>• To evaluate a decrease for oral rehydration solution (ORS) and/or intravenous (IV) fluid replacement therapy need

Endpoint(s)	**Primary Efficacy Endpoint:**<br>• Diarrheal stool output rate, defined as the total volume of diarrheal stools (mL, Grade 3 and above) divided by the number of hours between initiation of study product dosing and initiation of antimicrobial therapy.<br><br>**Primary Safety Endpoint:**<br>• Frequency and incidence of serious adverse events (SAEs) throughout the study<br><br>**Secondary Efficacy Endpoints:**<br>• Proportion of participants with moderate to severe diarrhea following cholera challenge, defined as >3 liters and >5 liters of loose stools , respectively, within 48 hours following challenge<br>• Attack rate of any diarrhea following cholera challenge, defined as the number of participants with either 2 or more loose stools (grades 3-5) totaling > 200 mL, or 1 loose (grade 3-5) stool > 300 mL, respectively, within 48 hours following challenge<br>• Area under the curve (AUC) of diarrheal stool volume between challenge dose and initiation of antibiotics<br>• Density of cholera organisms in post-challenge stool samples measured in stool samples via quantitative stool culture.<br>• To evaluate cholera illness in each participant after cholera challenge, using the following objective parameters:<br>1. Duration of diarrheal episode as defined by time to first formed stool<br>2. Total number of loose (Grades 3-5) stools<br>3. Occurrence of fever<br>4. Occurrence of vomiting<br><br>**Secondary Safety Endpoints:**<br>• To measure the occurrence of solicited adverse effects during the three days of oral dosing, through 8 hours following the last dose, which are attributed to iOWH032 and placebo, to include:<br>1. Nausea<br>2. Abdominal discomfort and pain<br>3. Occurrence of abdominal cramps<br>4. Occurrence of headache<br>5. Occurrence of malaise<br>6. Anorexia<br>7. Pollakiuria<br>8. Micturition urgency<br>9. Sinus tachycardia<br>10. Increased alertness<br>• To measure the frequency and incidence of unsolicited adverse effects up to 28 days after the last dose.<br><br>**Exploratory Endpoints:**<br>• To determine plasma levels of iOWH032 7±1 hours after the first and last dosing in all participants

	• Time (hours) to cessation of detectable cholera in stool, defined as the time of the first sample negative via quantitative culture, after which all following samples are also negative for cholera.<br>• ORS and/or IV fluid replacement measured in liters
**Description of Study Agent**	**Study Therapeutic:** iOWH032 is a synthetically manufactured small molecule designed to inhibit the cystic fibrosis transmembrane conductance regulator (CFTR) chloride channel. iOWH032 is a lipophilic product which is poorly soluble in aqueous solutions. iOWH032 is available as 250 mg tablets.<br>**Placebo:** Matching placebo tablets.<br>**Challenge Agent:** Freshly-harvested, wild-type *V. cholerae* El Tor Inaba strain N16961 will be the challenge agent. Strain N16961 will be delivered at 10^6 colony forming units (cfu), suspended in 30 mL of sodium bicarbonate solution (~1.3% $NaHCO_3$).
**Study and Participant Duration**	The entire study duration is anticipated to be approximately 8-9 months, inclusive of screening period up to 85 days prior to enrollment, at least 28 days of scheduled follow-up after challenge and a six-month phone follow-up. Each participant is anticipated to be in the study for a period of 6 months following administration of the challenge dose.
**Study Population**	Healthy adults, 18-44 years old, inclusive. Participants with Blood type O (carrying a higher risk for Cholera Gravis vs. Non-O) will not be preferentially selected, although randomization to treatment will be stratified by blood type status (O vs Non-O).
**Number of Participants**	48 participants will be enrolled in the study, split into two cohorts of 24 each. 24 total (12 in each cohort) will receive the test product (iOWH032) and 24 total (12 in each cohort) will receive placebo in a randomized double-blind fashion. Up to 5 volunteers will be admitted to the contained units as alternates to ensure a complete cohort, but discharged prior to challenge.
**Randomization**	Participants will be randomized in a 1:1 ratio in each of two sequential cohorts to receive either iOWH032 or placebo. The participants will be stratified based on blood type so that there are equivalent numbers of volunteers with blood type O in each study arm. A randomization list will be prepared allocating participant identification numbers to the study group. The randomization scheme will be provided to the unblinded research pharmacist(s).
**Bacteriological Assessments**	All loose stools (grade 3 and higher) will be weighed/measured for volume, and graded. A maximum of two stool samples daily will be examined for the presence of *V. cholerae* prior to receipt of antibiotics. Following antibiotics, if a subject cannot produce stool, a rectal swab will be performed every 12 hours in order to fulfillment of discharge criteria.
**Sample Size**	Study enrollment will proceed in two stages, with 24 participants randomized 1:1 to iOWH032 vs. placebo within each stage. Following completion of the first stage, an interim analysis of the primary efficacy endpoint will be conducted and the 2nd cohort will be enrolled, provided the study does not demonstrate either overwhelming efficacy or evidence of futility based on the results of the first cohort. A simulation-based approach was used to select

	boundaries for interim efficacy and futility, and to compute study power and maintain overall type I error at the nominal level. Using a stratified Wilcoxon rank-sum test (Van Elteren test) to compare diarrheal stool output rate between study arms in a group-sequential inferential framework, a total of 48 participants split equally between study arms provides 90% power to observe a 50% reduction in the cumulative diarrheal stool volume, based on prior data of placebo-treated participants challenged with the same strain as used in this study.

1 Study Rationale

This clinical trial is designed to be a Phase 2a, which will document the preliminary efficacy of oral doses of iOWH032 in diminishing the severity of illness of cholera, using a well-established human challenge model.

1.1 Schematic of Study Design

In Cohort 1, 24 eligible healthy male and female participants will be randomized 1:1 to be assigned to the iOWH032 (active treatment) or placebo. Following a favorable assessment of iOWH032 in an interim analysis, Cohort 2 will include an additional 24 healthy participants randomized 1:1 to iOWH032 or placebo. All participants will be challenged with 10^6 cfu of *V. cholerae* El Tor Inaba strain N16961. Blinded treatment will begin at the onset of (any) diarrhea symptoms or 48 hours after challenge, whichever is sooner, and consists of three oral doses per day, over three days. The dosage of iOWH032 is 500 mg (two 250 mg tablets) per dose.

A 3-day course of antimicrobial therapy will be initiated at approximately 4 days post challenge or sooner if indicated. The Principal Investigator will be able to initiate antibiotics upon severe cholera definition being satisfied. The blinded study treatment will continue during antimicrobial therapy. For participants that remain asymptomatic, the last dose of iOWH032 and first dose of antibiotic will be separated by 1-2 hours. Discharge from the Clinical Research Unit will be contingent upon each participant meeting all the following criteria:

- 3 consecutive stool cultures that are negative for growth of vibrios (with each culture separated by at least 12 hours)
- The absence of moderate or higher grade objective reactogenicity (diarrhea, fever, and vomiting) for at least 12 hours prior to discharge
- The completion of a 3-day course of antimicrobial (preferred agent, ciprofloxacin, 500 mg twice daily for 3 days) therapy

No. Participants	Treatment Group	Treatment duration	Challenge inoculum
Cohort 1			
12	iOWH032	3 days	N16961 at 10^6 cfu
12	Placebo	3 days	N16961 at 10^6 cfu
Cohort 2			
12	iOWH032	3 days	N16961 at 10^6 cfu
12	Placebo	3 days	N16961 at 10^6 cfu

2 KEY ROLES

**Principal Investigator**	[redacted]<br>Pharmaron CPC Inc.<br>[redacted]
**Co-Investigator**	[redacted]<br>Center for Vaccine Development<br>University of Maryland School of Medicine<br>[redacted]<br><br>[redacted]<br>University of Maryland School of Medicine<br>[redacted]
**Medical Officer**	[redacted]<br>PATH<br>[redacted]
**PATH Study Statistician**	[redacted]<br>PATH<br>[redacted]
**Project Coordinator**	[redacted]<br>PATH<br>[redacted]
**Clinical Site Monitor**	[redacted]<br>The Total Approach Inc.<br>[redacted]
**Drug Manufacturer Representative**	[redacted]<br>Syngene International<br>[redacted]
**Clinical Microbiology Laboratory**	Center for Vaccine Development and Global Health<br>Clinical Microbiology Laboratory<br>[redacted]
**Challenge Site**	Pharmaron CPC, Inc.<br>[redacted]
**Data Management**	Pharmaron CPC, Inc.<br>[redacted]
**Ethics Committee**	Advarra IRB<br>6940 Columbia Gateway Dr.<br>#110,<br>Columbia, MD 21046

3 INTRODUCTION: BACKGROUND INFORMATION AND SCIENTIFIC RATIONALE

Cholera is an acute gastrointestinal illness caused by the ingestion of food or water contaminated with the Gram-negative bacillus *V. cholerae*. Infection is associated with copious, toxin-mediated, watery diarrhea and can result in rapid and fatal dehydration if untreated. It remains a public health problem among underprivileged populations in many developing countries, and visitors to such areas. The cholera toxin (CT) of *V. cholerae* acts through the pathophysiologic mechanism of stimulation of the cystic fibrosis transmembrane conductance regulator (CFTR) chloride ion channel. The investigational new drug iOWH032 is being developed as an oral therapy for the treatment of acute secretory diarrhea in adults and children. While oral rehydration therapy (ORT) is a safe and cost-effective treatment for acute secretory diarrhea, it does not reduce diarrheal output, which may lead to poor outcome, compliance and acceptance of this therapy. An antisecretory agent that reduces dehydration, provides rapid symptomatic relief, and speeds recovery with a short course of therapy (e.g., two to three days) is expected to improve patient outcomes and compliance. While iOWH032 is intended as a treatment for acute secretory diarrhea in epidemic conditions of both adult and pediatric populations, iOWH032 will first be evaluated in adults in this Phase 2a study prior to a potential age-descending trial.

3.1 iOWH032 General Properties & Pharmacodynamics

iOWH032 is a synthetically manufactured small molecule designed to inhibit CFTR, a cyclic adenosine monophosphate (cAMP)-activated chloride ion (Cl^-) channel expressed in epithelial cells of mammalian airways, intestine, pancreas, skin, and testis. iOWH032's ability to inhibit the CFTR chloride channel was demonstrated in vitro using T84 colon carcinoma cells and Chinese hamster ovary (CHO) cells expressing human CFTR. The 50% inhibitory concentration in these assays ranged from 2-11 μM. iOWH032's activity in vivo was demonstrated in two disease models: the mouse closed-loop model and cecectomized rat open-loop model. The CFTR chloride channel in the gastrointestinal (GI) tract has both intracellular and extracellular (luminal) inhibitor sites. It is hypothesized that the inhibitory activity, and hence the pharmacodynamic response of iOWH032, will most likely be driven by GI lumen concentrations rather than plasma concentrations. The activity at the extracellular site will depend directly on the concentration in solution within the GI tract.

iOWH032 has a molecular weight of 545.18. It has a phenolic moiety and behaves as a weak acid (experimental pKa 7.84). It is a lipophilic molecule and is expected to be freely permeable across the lipid layer of biological membranes as demonstrated both by the parallel artificial membrane permeability assay and the use of Heterogeneous Human Epithelial Colorectal Adenocarcinoma cell line (Caco-2) cell line monolayers. It is also confirmed that iOWH032 was not a substrate for the efflux transporter, P-glycoprotein (efflux ratio < 2). iOWH032 does, however, show limited solubility (< 1 μM at pH 7.4), especially in acidic conditions. Its solubility increases in the presence of simulated fasted intestinal fluid (7.5 μM).

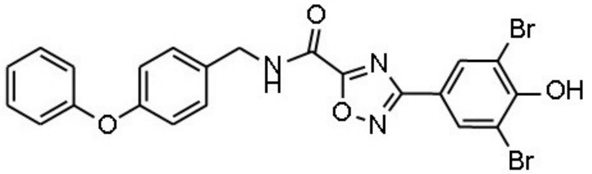

The structure of iOWH032 (3-(3,5-dibromo-4-hydroxyphenyl)-N-(4-phenoxybenzyl)-1,2,4-oxadiazole-5-carboxamide), CAS No. 1191252-49-9

iOWH032: molecular weight 545.18

The plasma half-life of iOWH032 administered orally in rats and dogs was in the range of 1 to 3 hours with maximum plasma concentrations (C_{max}) observed at approximately 1 to 2 hours post dose. The absolute bioavailability of an oral dose of 5 mg/kg was 38.1% in rats, and bioavailability of an oral dose of 3 mg/kg was 81.2% in dogs, showing absorption and bioavailability from the oral route. In vitro studies showed iOWH032 to be > 99% protein-bound in human, rat, and dog plasma. The clearance of iOWH032 was low relative to hepatic blood flow, with values of 0.452 L/hr/kg in rats and 0.096 L/hr/kg in dogs. However, despite this low clearance in both species, the corresponding half-lives of iOWH032 were short, 0.4 hours and 3.18 hours in rats and dogs, respectively, most likely due to the low volume of distribution.

3.2 Summary of Nonclinical Studies of Study Drug

The toxicology program included genotoxicity studies, acute (single-dose) and repeat-dose studies up to 14 days in duration, and reproductive toxicology studies in rats and rabbits. Animals received up to 2,000 mg/kg/day and there were no major toxicity findings with potential clinical significance.

iOWH032 was shown in an in vitro study to be a potent inhibitor of Cytochrome P450 2C9 (CYP2C9) (IC_{50} 0.08 µM) and, to a lesser extent, CYP2C19 (IC_{50} 24 µM). Concomitant administration of iOWH032 with drugs metabolized by CYP2C9 should be avoided. This includes warfarin, phenytoin, Nonsteroidal Anti-Inflammatory Drugs (NSAIDs), and other drugs described in the Appendix F. However, significant drug interactions in patients are not anticipated because of the short duration of treatment (≤ 3 days).

In the in vitro human ether-à-go-go–related gene (hERG) assay, the IC_{20} for the inhibitory effect of iOWH032 on hERG potassium currents was 1.87 µM and the IC_{50} was estimated to be > 4.95 µM, approximately 150-fold higher than the unbound concentration ($C_{max(unbound)}$ = 33.6 nM) of iOWH032 found in humans dosed with 500 mg every 8 hours. Cardiac safety was evaluated in telemetry-instrumented dogs in a cardiovascular safety pharmacology study and by monitoring ECGs during the repeat-dose toxicology study in dogs. No adverse effects of iOWH032 were observed on any cardiac parameter (blood pressure, heart rate, ECG including QTc) at doses up to 1000 mg/kg. In the clinical setting, protein binding is also expected to be high (99.79% in humans, based on in vitro studies) and, therefore, the free (unbound) concentration of iOWH032 in vivo is expected to be much lower than the concentration required to significantly inhibit the hERG potassium channel in vitro.

3.3 Summary of Clinical Studies of iOWH032

iOWH032 has been administered to 72 healthy adult volunteers in two Phase 1 studies conducted in the United States, and was found to be generally well-tolerated at single doses ranging from 30 mg to 1000 mg, and when administered for 3 days at doses ranging from 100 mg every 12 hours to 500 mg every 8 hours. A two-part bridging pharmacokinetic (DDP-CFT-PO-201) study was performed May-October 2013 in eight (8) healthy adult volunteers and twelve (12) adult male cholera patients in Bangladesh (NCT01823939).

3.3.1 iOWH032 Human Phamacokinetics Data

In the single ascending dose (SAD) study, doses of iOWH032 administered under fasted conditions exhibited absorption that was dose-independent with a median time of maximum plasma concentration (T_{max}) of 5 to 6.5 hours. Exposure to iOWH032 [C_{max} and $AUC_{0\text{-}inf}$] increased over the single-oral-dose range of 30 mg to 1000 mg in a less-than-dose-proportional manner. This was most pronounced at doses up to 300 mg; exposure at doses of 300 mg to 1000 mg appeared to increase in an approximately dose-proportional manner, but proportionality could not be confirmed statistically. Elimination half-life was similar across the tested dose range and was independent of

feeding status. However, there was a significant increase in iOWH032 exposure parameters [C_{max}: 3.85-fold and $AUC_{0\text{-}inf}$: 3.67-fold higher] when 500 mg iOWH032 was administered with a high-fat breakfast. The highest exposure to iOWH032 in this study was observed when 500 mg iOWH032 was administered with a high-fat breakfast [geometric mean: $AUC_{0\text{-}inf}$ 124,000 ng·hr/mL; C_{max} 7270 ng/mL].

In the multiple ascending dose (MAD) study, the median T_{max} ranged from 4 to 6 hours following single-dose administration on Day 1 and from 3.00 to 4.00 hours following multiple-dose administration on Day 4. Following T_{max} on Day 4, the iOWH032 plasma concentrations declined in a mostly monophasic manner. The elimination half-life of iOHW032 (10.3 to 11.7 hours) was independent of the administered dose. After single doses, geometric mean C_{max} values ranged from 2000 ng/mL (100 mg every 12 hours) to 5210 ng/mL (500 mg every 12 hours) and geometric mean AUC_{tau} values ranged from 14,900 ng·h/mL (100 mg every 12 hours) to 43,200 ng·h/mL (500 mg every 12 hours). After multiple dosing, geometric mean C_{max} values ranged from 2520 ng/mL (100 mg every 12 hours) to 8730 ng/mL (500 mg every 8 hours) and geometric mean AUC_{tau} values ranged from 23,000 ng·h/mL (100 mg every 12 hours) to 72,000 ng·h/mL (500 mg every 12 hours).

Regression analysis indicated that iOWH032 plasma exposure as measured by AUC_{tau} indicated a less-than-proportional increase over the studied dose range.

Human metabolism of iOWH032 was evaluated in vivo using liquid chromatography tandem mass spectrometry, demonstrating the major plasma and urinary metabolites to be a hydroxylated product with its glucuronide also being in urine but not in plasma. Thus, it would appear that in vivo, the major metabolite of iOWH032 circulating in human plasma is also the same as that circulating in rat plasma.

3.3.2 iOWH032 Human Safety Data

In the SAD study, the most common adverse event (AE) was pollakiuria (extraordinary daytime urinary frequency). In the 30 mg dose group, 4 of 6 participants, all who received active drug and none who received placebo, reported pollakiuria beginning 3 to 6 hours following receipt of the single dose and persisting for 19 to 30 hours. Three of these same participants also reported micturition urgency, and two reported an increase in stool frequency (3 formed stools in one day rather than their usual 1 formed stool). All adverse events were rated as mild in severity and resolved spontaneously. Repeated urinalyses (including urine specific gravities), urine cultures, serum electrolytes, blood urea nitrogen (BUN), creatinine, and complete blood counts were all normal throughout the period of time the participants were symptomatic. Plasma concentrations of iOWH032 in these 4 participants ranged from approximately 400–900 ng/mL. No urinary symptoms were reported by participants enrolled in the 300, 500, or 1000 mg dose cohorts in the SAD portion of the study. One participant in the food effects portion of the study reported pollakiuria following his receipt of the 500 mg dose in both the fed and fasted portions of the study. This participant reported onset of symptoms beginning approximately 4 hours after dosing in each treatment period and on both occasions, reported being symptomatic for approximately 4 days. As observed in the 30 mg dose cohort, repeated urinalyses (including urine specific gravities), urine cultures, serum electrolytes, BUN, creatinine, and CBCs were normal and the participant's symptoms resolved spontaneously. This participant's maximum iOWH032 plasma concentrations were 6492 ng/mL in the fed state and 3432 ng/mL in the fasting state.

One 19-year-old female with no history of cardiovascular disease experienced isolated, asymptomatic premature ventricular contractions (PVCs) at 2 and 3 hours following a single dose of 100 mg of iOWH032. ECGs that were obtained at screening and pre-dose were normal except for intermittent sinus tachycardia. On telemetry during the next 24 hours, she persisted in having

isolated asymptomatic PVCs without any accompanying symptoms of palpitations, chest pain, or dizziness. A thorough cardiac evaluation by a board-certified cardiologist revealed a normal cardiac exam, normal stress echocardiogram, normal ECG, and no evidence of structural cardiac disease.

In the MAD study, the most frequently occurring treatment-emergent adverse events (TEAEs) reported in 2 or more participants overall were application site irritation (at the site of electrocardiogram patches) (11/40, 27.5%), sinus tachycardia (3/40, 7.5%), catheter site pain (2/40, 5.0%), decreased appetite (2/40, 5.0%), and headache (2/40, 5.0%). Treatment-emergent sinus tachycardia and decreased appetite were not reported in participants who received placebo.

Three mild TEAEs of sinus tachycardia were reported by a single participant in each of the following 3-dose cohorts: 300 mg iOWH032 every 8 hours, 500 mg iOWH032 every 12 hours, and 500 mg iOWH032 every 8 hours. No clinically significant changes in blood pressure or other ECG parameters accompanied the tachycardia. Two of these participants had the maximum iOWH032 exposures in their dose cohorts and had close to the maximum overall exposure of all participants during the time of their sinus tachycardia; 1 of the 2 participants experienced an accompanying TEAE of increased alertness and the other participant reported no accompanying symptoms. The third participant who had a TEAE of sinus tachycardia reported an accompanying symptom of a "stimulated feeling" and had iOWH032 exposures that approximated the median for his dose cohort and also for all participants. One participant reported a TEAE of palpitations that was unaccompanied by tachycardia. A slight upward trend in average pulse rate change from baseline was observed with increasing iOWH032 plasma concentration, but it was not statistically significant. Mean systolic blood pressure decreased by approximately 10 mmHg in the 300-mg and 500-mg iOWH032 every-8-hour dose cohorts compared to the pooled placebo group, beginning approximately 1 hour following receipt of Dose 1 and persisting until approximately 112 hours after Dose 1 (approximately 40 hours after the receipt of Dose 10 of iOWH032). None of the individual decreases were considered clinically important decreases in systolic blood pressure and thus were not reported as adverse events.

In the PK bridging study (Bangladesh, DDP-CFT-PO-201), one SAE was reported in a patient with cholera. This SAE of elevated serum creatine kinase/creatine phosphokinase was deemed unlikely related to the drug, as there were no clinically relevant features or changes in ECG parameters that were found to be associated. No treatment was provided to the participant in response to the event. No action was taken with regards to the study drug due to the event.

3.4 Background Information on the Cholera Challenge Model

Cholera continues to be a major public health problem in nearly all developing countries including the Western hemisphere, afflicting both children and adults, and is an endemic disease in over 100 countries of the world. There are over 200 serogroups of cholera, based on the polysaccharide O-antigen. Epidemic cholera is associated with the O1 and, more recently, O139 serogroups. The O1 serogroup is further classified by biotype, classical or El Tor, and within this biotype by serotype, Ogawa or Inaba. Carefully conducted human challenge studies have been used to demonstrate the efficacy of cholera vaccine candidates. Pioneered at the University of Maryland, Baltimore (UMB), Center for Vaccine Development (CVD), cholera challenge models have been safely and successfully used for over 40 years. Investigators at CVD developed a challenge model using freshly harvested *V. cholerae* El Tor Inaba strain N16961. Previous studies demonstrated a clear dose-response (Table 1) and indicated that a 10^6 cfu challenge dose of freshly harvested *V. cholerae* strain N16961 produced consistent disease (Table 2).

Table 1: Clinical and bacteriological responses of healthy volunteers to various doses of freshly harvested *V. cholerae* El Tor Inaba strain N16961

Inoculum	Attack Rate for Diarrhea	Mean Incubation (Hours)	Mean Diarrheal Stool Volume (Liters)	Mean Number Diarrheal Stools (per day)	Vomiting	Fever	Positive Stool Culture
10^3	4/6	33	0.9	5.1	0/6	0/6	6/6
10^4	4/5	36	1.1	6.5	0/5	0/5	4/5
10^5	3/5	18	3.1	15	0/5	0/5	4/5
10^6	9/10	25	3.2	12.9	2/10	2/10	10/10

Table 2: Diarrheal attack rates with freshly harvested *V. cholerae* El Tor Inaba strain N16961, across multiple challenge studies

CVD Protocol	Inoculum	Attack Rate for Diarrhea
3002A	0.62×10^6	5/5
3005B	1.0×10^6	4/5
5009	1.9×10^6	6/8
5011	2.0×10^6	5/7
5013	1.4×10^6	7/8
5025	2.2×10^6	9/9
6001	2.3×10^6	6/7
6005	1.98×10^6	10/11
6011	1.74×10^6	6/6
7002	1.74×10^6	7/7
7004	1.79×10^6	6/8
9003	1.0×10^6	7/8
11002	1.07×10^6	13/13
	TOTAL	91/102 (89%)

It is intended that 10^6 cfu challenge doses of freshly harvested *V. cholerae* strain N16961 will be administered to study participants in this clinical trial, to allow for a preliminary assessment of the efficacy of iOWH032.

3.5 Potential Risks and Benefits

No benefits are expected to participants for their participation in this research study. The study screening visit will be free of charge, but that if any clinical abnormalities are noted during that visit that participants will be referred to their own physician for further follow-up.

There is the potential for some therapeutic efficacy against acute secretory diarrhea with oral doses of iOWH032, but this has not been proven. Participants that undergo challenge with cholera should expect to have diarrhea, in some cases the diarrhea can be severe. Cholera illness may also be associated with nausea, vomiting, abdominal cramping or discomfort, loss of appetite, muscle aches, and tiredness. The electrolyte losses with the secretory diarrhea may result in cardiac conduction abnormalities, if uncorrected. Intravascular volume losses with diarrhea can be associated with postural hypotension, shock, and death, if uncorrected. In this study, participants will be closely monitored to detect and prevent such effects, with rescue treatment readily provided (See section 9).

3.5.1 Known Potential Risks

Oral doses of iOWH032. Doses of 500 mg administered every 8 hours for 10 consecutive doses (3 days plus a single dose on Day 4) have generally been well tolerated in the limited studies that have been performed. Potential known AEs of iOWH032 are pollakiuria, micturition urgency, sinus tachycardia and increased alertness. There may be other unknown risks, discomforts, or side effects from iOWH032. Any significant adverse events, which are deemed possibly related to iOWH032, will be disclosed, as appropriate.

Challenge with *V. cholerae* strain N16961. The intended dose of 10^6 cfu is expected to elicit acute watery diarrhea within 18-48 hours of ingestion; about 20% are expected to have severe cholera. Persons that are blood group O are known to be more likely to have severe cholera. Nausea or vomiting, abdominal cramping or discomfort, and loss of appetite are also common symptoms that accompany cholera. Fever is a rarer symptom with cholera. The signs of severe dehydration can involve dry mouth, decreased urine, thirst, cold clammy skin, hypotension, lethargy, stupor, and muscle cramping. The complications of too much fluid lost through excessive diarrhea include hypoglycemia (low blood sugar), acidosis, kidney failure, pulmonary edema (fluid in lungs), arrhythmia (heart rhythm abnormalities), coma, and death. Anticipated dehydration will be managed with aggressive fluid rehydration (oral and/or intravenous) with potassium repletion. Early antibiotic therapy will be provided to participants that achieve severe cholera illness; otherwise all participants will be treated with antibiotics at approximately 4 days post-challenge. It is expected that shedding will occur in the stool; however, transmission from person to person is mitigated by housing the participants on the Clinical Research Unit until they meet the protocol discharge criteria. Participants will be counselled about use of standard hygiene practices (i.e., hand washing with soap and water after defecation), to decrease the likelihood of transmission of the strain person to person.

Risk of Allergic Reaction. There is a very rare risk for an allergic reaction to the study drug iOWH032, the placebo, the challenge inoculum, or the antibiotic. Allergic reactions generally can range from mild (for example, skin rash, itching, swelling, or numbness) to severe (for example, low blood pressure, difficulty breathing, shock, heart arrest, or death). The symptoms of a mild allergic reaction typically go away without treatment, but some cases may require treatment with antihistamines or steroids (medications used to treat allergic reactions). In the case of more severe allergic reactions such as shock, immediate and intensive medical treatment is necessary. Although the risk of an allergic reaction is very small, epinephrine, antihistamines, and other equipment will be available to treat anaphylaxis or immediate hypersensitivity reactions.

Risk with Pregnancy. The study drug iOWH032 has not been evaluated in pregnant women. Therefore, the risk to the unborn fetus or pregnant mother is unknown. Cholera infection in a pregnant woman can harm the fetus. All female study participants will be tested for pregnancy, during screening and one day before the challenge. To be eligible, female study participants must be using a highly effective form of contraception for the duration of the trial and the four weeks before and after the challenge. If a pregnancy occurs between challenge and 28 days post-challenge, it will be reported immediately (within 24 hours of identification) to the sponsor’s medical officer. A pregnancy is reported as an AE or SAE only when there is suspicion that the investigational product may have interfered with the effectiveness of contraception or there was a serious complication in the pregnancy including a spontaneous abortion or an elective termination for medical rationale, or a birth defect in the baby.

Risk with Blood Draws. Blood will be drawn at several times during the study and may also be performed to help manage the diarrheal illness. The drawing of blood may cause pain, bruising, feeling faint, fainting, needle site infections, swelling, and rarely other infections. Bruising at the site

of blood drawing can be prevented by applying pressure for several minutes. To reduce the risk of infection, the skin site is prepared with an alcohol wipe and sterile technique is used.

Rectal swab (if a stool sample is not provided). Rectal swabs may be performed as part of the requirement for discharge criteria (i.e., three sequential negative stool cultures separated by 12 hours). The rectal swab may cause the sensation of rectal pressure, but is not painful.

Risks to Confidentiality. Personal health information will be collected as a part of this study and efforts will be made to maintain confidentiality. There is a small risk of loss of confidentiality that an unauthorized person may gain access to viewing the research records. In order to maintain confidentiality, all study records will be stored in a secure location, such as a locked office and/or locked cabinet. Electronic data will be password-protected. Study records and specimens obtained will be coded. Research records will only be shared with authorized personnel and only in connection with carrying out the obligations relating to the study. Every effort will be made to keep the records as confidential as possible, within the limits of the law.

3.5.2 Known Potential Benefits

This is a healthy volunteer study which does not provide any guarantee of benefit to the participants. The benefit is largely the scientific knowledge to be gained from the study.

3.6 Overall Development Strategy

To predict the usefulness of iOWH032 as a public health tool in the treatment of cholera, PATH proposes to first determine the degree of protection provided against moderate to severe diarrheal disease with *V. cholerae* using a human challenge study as a Phase 2a trial, before proceeding to field studies. While a cholera challenge study will not answer all of the questions regarding the use of an antisecretory drug in the field, it will provide an answer on the degree of reduction of diarrheal disease under these optimal conditions. If it is not efficacious in the challenge model it is unlikely to be effective in the field. Therefore, a positive result in the challenge model would justify further field efficacy trials for fluid reduction in cholera.

If efficacy is found in the challenge study, the next step is to perform field trials in areas where cholera occurs. Our goal is to achieve US approval by submitting a New Drug Application to the FDA, followed by prequalification by the World Health Organization (WHO) to support product acquisition by UNICEF and other humanitarian agencies for its distribution to low- and middle- income countries.

4 HYPOTHESIS, OBJECTIVES AND PURPOSE

4.1 Study Hypothesis / Hypotheses

- iOWH032 will demonstrate reduction in fluid output against cholera diarrhea
- iOWH032 will be safe and well-tolerated

4.2 Study Objectives

4.2.1 Primary Efficacy Objective

- To measure the rate and extent of diarrhea following cholera challenge, in participants treated with iOWH032 compared to placebo

4.2.2 Primary Safety Objective:

- To evaluate safety of oral doses of iOWH032 compared to placebo

4.2.3 Secondary Efficacy Objectives:

- To evaluate additional measures of efficacy against cholera illness following cholera challenge, among those randomized to be treated with iOWH032 versus placebo

4.2.4 Secondary Safety Objective:

- To evaluate tolerability of oral doses of iOWH032 compared to placebo

4.2.5 Exploratory Objectives:

- To evaluate the pharmacokinetic (pK) properties of iOWH032
- To evaluate time of cessation of cholera organism in stool after challenge
- To evaluate a decrease for ORS and/or IV fluid replacement therapy need

5 STUDY DESIGN AND ENDPOINTS

5.1 Description of the Study Design

This is a single-center, randomized, double-blind, placebo-controlled challenge and therapeutic trial assessing diarrheal output and clinical symptoms when given either placebo or oral iOWH032 500 mg (two 250 mg tablets) every 8 hours for three days starting at the onset of any diarrhea (upon first Grade 3-5 stool, regardless of volume) stool or 48 hours after ingesting an inoculum of *V. cholerae* N16961, whichever is sooner, and consists of three oral doses per day, over three days.

Test Article (TA)	N	Dose
iOWH032	24	500 mg (two 250 mg tablets) every 8 hours for 3 days with meals
Placebo	24	Two placebo tablets every 8 hours for 3 days with meals
Challenge	48	10^6 CFU *Vibrio cholerae* N16961, once on challenge day

5.2 Study Endpoints

5.2.1 Primary Efficacy Endpoints

- Diarrheal stool output rate, defined as the total volume of diarrheal stools (mL, Grade 3 and above) divided by the number of hours between initiation of study product dosing and initiation of antimicrobial therapy

5.2.1 Primary Safety Endpoint:

- Frequency and incidence of serious adverse events (SAEs) throughout the study

5.2.2 Secondary Efficacy Endpoints:

- Proportion of participants with moderate to severe diarrhea following cholera challenge, defined as >3 liters and >5 liters of loose stools, respectively, within 48 hours following challenge
- Attack rate of any diarrhea following cholera challenge, defined as the number of participants with either 2 or more loose stools (grades 3-5) totaling > 200 mL or 1 loose (grade 3-5) stool > 300 mL, respectively, within 48 hours following challenge

- Area under the curve (AUC) of diarrheal stool volume between challenge dose and initiation of antibiotics
- Density of cholera organisms in stool samples measured in stool samples via quantitative stool culture
- To evaluate cholera illness in each participant after cholera challenge, using the following objective parameters:
  1. Duration of diarrheal episode as defined by time to first formed stool
  2. Total number of loose (Grades 3-5) stools
  3. Occurrence of fever
  4. Occurrence of vomiting

5.2.3 Secondary Safety Endpoints:

- To measure the occurrence of solicited adverse effects during the three days of oral dosing, through 8 hours following the last dose, which are attributed to iOWH032 and placebo, to include:
  1. Nausea
  2. Abdominal discomfort and pain
  3. Occurrence of abdominal cramps
  4. Occurrence of headache
  5. Occurrence of malaise
  6. Anorexia
  7. Pollakiuria
  8. Micturition urgency
  9. Sinus tachycardia
  10. Increased alertness
- To measure the frequency and incidence of unsolicited adverse effects up to 28 days after the last dose.

5.2.4 Exploratory Endpoints

- To determine plasma levels of iOWH032 7±1 hours after the first and last dosing in all participants
- Time (hours) to cessation of detectable cholera in stool, defined as the time of the first sample negative via quantitative stool culture, after which all following samples are also negative for cholera.
- ORS and/or IV fluid replacement in measured liters

6 STUDY ENROLLMENT AND WITHDRAWAL

6.1 Participant Inclusion Criteria

1. Willing and able to understand and provide written informed consent
2. Healthy male and female adults, age 18 to 44 years (inclusive), without clinically significant medical history, physical or clinical laboratory abnormalities (as per protocol-defined acceptable ranges), and protocol-defined abnormal electrocardiogram results at screening
3. All women must have a negative serum pregnancy test at screening and one day prior to challenge.
4. Agreement by participants to use an adequate method of contraception[§] during the study and for 4 weeks before and after the challenge.

5. Able to pass a written examination (comprehension assessment test) with a score of ≥70%, in order to demonstrate their comprehension of this study. If a participant scores at least 50%, then they will be given one more opportunity to re-test after further re-education.
6. Willing and able to comply with the study requirements and procedures.

§ Adequate contraception is defined as a contraceptive method with failure rate of less than 1% per year when used consistently and correctly and when applicable, in accordance with the product label; includes, but is not limited to, barrier with additional spermicidal foam or jelly, intrauterine device, hormonal contraception (started at least 4 weeks prior to study enrollment), or women who have intercourse limited to men who underwent vasectomy.

6.2 Participant Exclusion Criteria

1. Clinically significant history of immunodeficiency, cardiovascular disease, respiratory disease, endocrine disorder, liver disease, renal disease, gastrointestinal disease, anal or rectal disorders, neurologic disease,
2. Current nicotine use or drug, alcohol abuse within the past 6 months
3. Recipient of bone marrow or solid organ transplant
4. Use of systemic chemotherapy in the past 5 years
5. Has a malignancy (excluding localized non-melanoma skin cancers) or lymphoproliferative disorders diagnosed or treated within the past 5 years
6. Received or plans to receive systemic immunosuppressive therapy, radiation therapy, parenteral or high-dosage inhaled steroids (> 800 μg/day of beclomethasone dipropionate or equivalent) within 6 months prior to the enrollment through 28 days after challenge
7. Have a history of hospitalization for psychiatric illness, suicide attempt, or confinement for danger to self or others, within the past 10 years. Participants with a psychiatric disorder (not meeting exclusion criteria, e.g., attention-deficit hyperactivity disorder) that is controlled for a minimum of 3 months and the investigator has determined that the participant's mental status will not compromise the participant's ability to comply with protocol requirements may be enrolled
8. Have an elevated blood pressure, systolic ≥150 mmHg or diastolic ≥90 mmHg, before challenge
9. Taking any of the drugs listed in Appendix F that are metabolized by CYP2C9 or any of the following psychiatric medications: aripiprazole, carbamazepine, chlorpromazine, chlorprothixene, clozapine, divalproex sodium, fluphenazine, haloperidol, lithium carbonate, lithium citrate, loxapine, mesoridazine, molindone, olanzapine, perphenazine, pimozide, quetiapine, risperidone, thioridazine, thiothixene, trifluoperazine, triflupromazine, or ziprasidone
10. History of Guillain-Barré Syndrome
11. Too low or too high a BMI (BMI < 18.5 or > 39)
12. Has an abnormal stool pattern defined as fewer than 3 stools per week or more than 2 stools per day within the past 6 months, and any loose stools (grade 3 or higher) during the 1–2 day acclimation period before challenge
13. Has regularly used laxatives in the past 6 months
14. Has a history of eating disorders (e.g. anorexia or bulimia) within the past 10 years
15. Known allergy or previous severe adverse effect to all of the following antibiotics: ciprofloxacin (or quinolones), azithromycin and doxycycline.
16. Previously received a licensed or investigational cholera vaccine, within 10 years
17. History of cholera or enterotoxigenic *Escherichia coli* (ETEC) infection (lab-confirmed natural infection or experimental challenge), within 10 years

18. Travel to a cholera-endemic area in the past 5 years
19. Pregnant or nursing
20. Positive serology for human immunodeficiency virus (HIV), hepatitis B antigen, or hepatitis C antibody
21. Protocol-defined (appendix B) clinically abnormal 12-lead ECG at screening in the judgment of the Investigator, or based on the formal 12-lead ECG reading by a cardiologist; history of any cardiac abnormalities, including conduction abnormalities such as Wolff-Parkinson-White, dysrhythmias, or coronary artery disease
22. Presence of a clinically significant abnormality on physical examination, including (but not limited to): pathologic heart murmur, lymphadenopathy, hepatosplenomegaly, large abdominal scar of unclear origin
23. Has poor venous access, defined as the inability to obtain screening blood tests after three attempts
24. Currently on, or plans to be on, antibiotics (e.g., doxycycline) within 14 days prior to challenge and through 28 days after challenge
25. Presence of an acute illness or fever (>100.4°F) within 72 hours of admission to the inpatient Clinical Research Unit
26. Taking any prescription or over-the-counter medications that contain aspirin, non-steroidal anti-inflammatory drugs (NSAIDs), antacids, proton pump inhibitors (PPIs), anti-diarrheals, etc. within 72 hours prior to challenge
27. Received an investigational product within 30 days prior to randomization (for the monoclonal antibodies- 90 days prior to randomization) or planning to participate in another research study involving investigational product during the conduct of this study
28. Participants must not have donated blood in 8 weeks prior to study entry and agreed to not donate blood during and for 4 weeks following their active participation in this study
29. Lack of ability to fully understand the informed consent
30. Any other condition(s) that in the opinion of the investigator would jeopardize the safety or rights of a participant participating in the trial or would render the participant unable to comply with the protocol

6.3 Strategies for Recruitment and Retention

Volunteers will be recruited from the greater Baltimore area. They will be recruited using standard procedures for Pharmaron clinical trials. Volunteers for inpatient challenge studies are carefully screened on the details of the protocol, their time commitment, and the nature of the confinement on a quarantine ward. At the end of screening volunteers will take a test to make sure that they understand the study. They are also screened for general health. Volunteers receive a stipend for each visit and day in confinement that has been approved by the Advarra IRB.

6.4 Withdrawal from Study

6.4.1 Reasons for Withdrawal or Termination

Participation in the study is strictly voluntary. Participants have the right to withdraw from the study at any time and for any reason, without penalty.

The Principal Investigator (PI) and/or designee may withdraw a participant from continuing in the study for the following reasons:

- Pregnancy

- Significant study intervention non-compliance
- If any clinical adverse event (AE), laboratory abnormality, or other medical condition or situation occurs such that continued participation in the study would not be in the best interest of the participant
- Disease progression which requires discontinuation of the study intervention
- If the participant meets an exclusion criterion (either newly developed or not previously recognized) that precludes further study participation
- It is considered to be in the participant's best interest, or if the participant is not willing or able to comply with the study requirements.

6.4.2 Handling of Participant Withdrawals or Termination

Every effort will be made to undertake protocol-specified safety follow-up procedures to capture AEs, serious adverse events (SAEs), and unanticipated problems (UPs). In the event of withdrawal from study, reasonable efforts should be made to conduct the following procedures:

- Update any ongoing AE/SAEs that remain ongoing at time of participant's last visit prior to withdrawal
- Query about AEs, SAEs and concomitant medications if the interval between the participant's last visit and the time of withdrawal is within the protocol defined reporting period
- Physical examination
- Blood for safety laboratory testing if withdrawal occurs before Visit 2
- Update contact information

The reason for participant discontinuation or withdrawal from the study will be recorded on the Case Report Form (CRF). Participants who sign the informed consent form and are randomized but do not receive the challenge may be replaced. Participants who sign the informed consent form, and are randomized and receive the challenge, and subsequently withdraw, or are withdrawn or discontinued from the study, will not be replaced.

6.5 Premature Termination or Suspension of Study

Although the study Sponsor has every intention of completing this study, it may be temporarily suspended or prematurely terminated if there is sufficient reasonable cause. The Sponsor reserves the right to terminate the study at any time for clinical or administrative reasons. Written notification, documenting the reason for study suspension or termination, will be provided by the suspending or terminating party to PI and the FDA. If the study is prematurely terminated or suspended, the PI and/or designee will promptly inform the IRB and will provide the reason(s) for the termination or suspension.

Circumstances that may warrant termination or suspension include, but are not limited to:

- Determination of unexpected, significant, or unacceptable risk to participants
- Demonstration of efficacy that would warrant stopping
- Insufficient compliance to protocol requirements
- Data that are not sufficiently complete and/or evaluable
- Determination of futility

Study may resume once concerns about safety, protocol compliance, data quality are addressed and satisfy the sponsor, IRB and/or FDA.

7 STUDY AGENTS

7.1 Study Agent and Control Description

7.1.1 Product Description

The iOWH032 drug product is a chemically and physically stable immediate-release oral tablet. The key characteristics of iOWH032 tablets include fast disintegration time (less than five minutes), similar to micro-suspension (which was used for phase 1 SAD and MAD studies), and good chemical and physical stability under long-term storage conditions. The placebo will be matched in terms of visual appearance, disintegration time, and taste.

7.1.2 Manufacturer

The iOWH032 drug substance for this study was manufactured by Albany Molecular Research, Inc., Albany, New York, United States (Batch Number 6798).

The iOWH032 drug product and placebo for this study will be manufactured by Syngene International Ltd., Bengaluru, India (Batch Number TBD)

7.1.3 Acquisition

iOWH032 drug product and placebo will be shipped to the site from Syngene International Ltd., Bengaluru, India.

7.1.4 Formulation, Appearance, Packaging, and Labeling

Each 250 mg iOWH032 tablet contains 40% of iOWH032 (w/w) and 60% excipients (w/w) including Mannitol 60®, Avicel® PH-101, Kollion CL-F®, Kollidon30®, Aerosil® 200 Pharma, and magnesium stearate. The matching placebo is made of the same excipients, but without the active drug substance.

Uncoated iOWH032 tablets and matching placebos have a white, circular, biconvex appearance and their manufacturing process involves blending, wet granulation, drying, milling, and tableting.

All packaging and labeling operations will be performed according to current Good Manufacturing Practices (cGMPs) and Good Clinical Practices (GCPs) and local requirements.

All drug packages should be inspected on receipt at the study site for signs of damage. Evidence of damaged study drug is to be reported to the sponsor and stored under the labeled conditions until further instructions have been provided.

7.1.5 Product Storage and Stability

The study drugs must be kept in a securely locked, substantially constructed enclosure to which access is limited (e.g., locked cabinet). The investigator will take adequate precautions, including locked storage, to prevent theft or diversion of the study drug, consistent with International Council on Harmonisation-Good Clinical Practice (ICH-GCP). Within the locked storage area, study drug will be stored in accordance with the conditions specified on the drug labels (i.e. stored in tightly closed bottles at or below 30◦C without direct exposure to sunlight) until used.

On receipt of the study drug, the investigator or designee will perform an inventory of the shipment, comparing the shipment inventory to study drug actually received, and complete and sign an inventory log. The investigator or designee must count and verify that the shipment contains all of the items appearing on the shipment inventory. The investigator must immediately notify PATH or the drug distribution contractor of any damaged or unusable study drug that the site receives, and document any damaged or unusable study drug in the inventory log.

The investigator or designee will retain a copy of the shipment inventory received with the drug supply in the study file, send a copy to the PATH-designated representative, and forward the original to the drug distribution center.

Previous lots of iOWH032 have demonstrated good physical and chemical stability under a variety of storage conditions (25°C/60% RH, 30°C/75% RH, 40°C/75% RH) in studies lasting 18 months. The current lot will be under stability for 18 months at 25°C/60% RH.

7.1.6 Preparation

Dose preparation, as described in detail in the Pharmacy Manual, will be carried out by a qualified unblinded research pharmacist.

7.1.7 Dosing and Administration

All participants will receive 500 mg iOWH032 (two 250 mg tablets) or matching placebo three times a day with meals, approximately 8 hours part, for 3 consecutive days according to randomized treatment assignment. The treatment will begin at the onset of diarrhea symptoms (first Grade 3 or over stool) or 48 hours after challenge, whichever is sooner.

7.1.8 Route of Administration

iOWH032 and placebo tablets will be administered orally.

7.1.9 Starting Dose

All doses are set at 500 mg (two 250 mg tablets) for iOWH032 or two matching placebo tablets.

7.1.10 Dose Adjustments/Modifications/Delays

No dose adjustments are allowed. A delay in dosing for safety reasons is allowed, in case of delayed dosing those participants will be removed from the pK population. PATH Medical Officer should be notified if the investigator delays dosing due to safety reasons. PATH Medical Officer, in collaboration with the investigator, will determine if dosing can be reinitiated. The date and reason that dosing was delayed must be recorded in the source documentation and case report form, as applicable.

7.1.11 Duration of Therapy

Study drug will be administered three times a day, approximately 8 hours part, for 3 consecutive days.

7.1.12 Tracking of Dose

Each dosing will be directly observed by study staff. Appropriate staff will record each study drug administration (date and stop/start times) in the participant's chart (source document) and electronic case report form (eCRF).

7.2 Challenge Strain Description

7.2.1 Product Description

Freshly-harvested, wild-type *V. cholerae* El Tor Inaba strain N16961 will be the challenge agent. Strain N16961 will be delivered at 10^6 cfu, suspended in 30 mL of sodium bicarbonate solution (~1.3% $NaHCO_3$).

7.2.2 Manufacturer

Investigators at CVD developed the cholera challenge model using freshly harvested *V. cholerae* El Tor Inaba strain N16961. For this study, CVD will develop the master cell bank to be administered to participants.

7.2.3 Acquisition

Freshly-harvested, wild-type *V. cholerae* El Tor Inaba strain N16961 will be prepared by University of Maryland, Baltimore (UMB), Center for Vaccine Development (CVD). Vials of strain N16961 are stored in the CVD Clinical Microbiology Lab at -70°C (-60°C or colder).

7.2.4 Formulation, Appearance, Packaging, and Labeling

Final containers hold 1 mL strain N16961 with ~10^9 cfu/mL in cryovials with gasketed screw-caps. The following information is contained on the label of each vial: “New Drug Limited by Federal (US) Law to Investigational Use”, Lot #xxx, Manufacture date xx/xxx/xxxx.”

7.2.5 Product Storage and Stability

It is anticipated that the cell bank will be stable for years if maintained frozen at -70°C (-60°C or colder).

7.2.6 Preparation

Two days before the challenge date, inoculum preparation will begin from a WCB vial of frozen *V. cholerae* strain N16961.The WCB strain will be plated onto Hy-Soy Agar plates. After incubation at 37°C overnight, one colony will be selected and will be plated onto Hy-Soy Agar plate and Thiosulfate-citrate-bile salts-sucrose agar plate (TCBS). After incubation at 37°C overnight, colonies that exhibit characteristic *Vibrio* appearance will be picked for heavy growth of organisms as well as for identity tests (morphology, Gram’s staining and agglutination with *Vibrio* antiserum). From the heavily seeded Hy-Soy Agar plates, growth will be harvested into sterile phosphate buffered saline (PBS). The bacterial suspension will be diluted with sterile PBS to produce a turbidity measured by optical density (OD) 660 nm to correspond to the desired bacterial count of a target of 10^6 CFU/mL. The final stock dilution of challenge inoculum will be in a tube labeled with: organism identity, concentration, date, time, and “Caution: New Drug (limited to investigational use)”. This tube will be kept at room temperature and transported to the Research Isolation Ward. Inoculum colony counts will be performed before and after challenge to document the final concentration of inoculum.

7.2.7 Dosing and Administration

Fasting eligible participants will drink 120 mL of sodium bicarbonate solution (~1.3% $NaHCO_3$); approximately 1 minute later, participants will ingest 10^6 cfu of the challenge strain suspended in 30 mL of sodium bicarbonate solution (~1.3% $NaHCO_3$). Participants will have nothing by mouth, except water, for 90 minutes before or following ingestion of the challenge inoculum.

7.2.8 Route of Administration

The challenge strain will be administered orally.

7.2.9 Starting Dose

The challenge dose is a single inoculum of 10^6 cfu of *V. cholerae* El Tor Inaba strain N16961.

7.2.10 Dose Adjustments/Modifications/Delays

No dose adjustments are allowed. Challenge will not be re-administered if a subject who vomits the challenge dose shortly after dosing.

7.2.11 Duration of Therapy

The challenge inoculum will be administered in a single dose only.

7.2.12 Tracking of Dose

The challenge inoculum dosing will be directly observed by study staff. Appropriate staff will record each study drug administration (date and stop/start times) in the participant's chart (source document) and eCRF.

7.3 Study Agents Accountability Procedures

The site pharmacist will maintain complete records of all study drug received from the Sponsor and will be responsible for maintaining an accurate record of the randomization codes, inventory, and an accountability record of iOWH032, challenge material and placebo for this study. The site pharmacist will also be responsible for ensuring the security of these documents. At the end of the study, the site will receive instruction from the Sponsor regarding the final disposition of any remaining study drug.

8 STUDY PROCEDURES AND SCHEDULE

8.1 Study Procedures/Evaluations

8.1.1 Study Specific Procedures

Study procedures and evaluations done as part of the study include:

- Medical history: a comprehensive medical history will be collected by interview with the participant including participation in clinical trials, surgery, previous hospitalization, allergy to food/drugs, and history of any chronic or recurrent medical conditions.
- Interval medical history: inquiry regarding changes since the last medical history discussion including signs and symptoms.
- Medication history from the past 4-weeks and current medications taken, including prescription and over-the-counter medications.
- Physical examination: full physical examination will include assessment of vital signs, head, eyes, ears, nose, oropharynx, neck, chest (auscultation), lymph nodes (neck, supraclavicular, axillary, inguinal), abdomen (auscultation and palpation), musculoskeletal, skin, and neurological.
- ECG and pregnancy testing
- Safety Laboratory: Blood, stool, and urine specimens collected and evaluated
- Blood sampling for study drug pK analysis
- Cholera Challenge, iOWH032 and placebo administration and antibiotic treatment

8.1.2 Standard of Care Study Procedures

As all participants in this trial are healthy volunteers, there are no standard of care procedures that would have been conducted if the individuals were not enrolled into the trial.

Standard of care procedures for the treatment of cholera include management of hydration levels, electrolyte balance, and also prescription with a course of antimicrobials. Details of these procedures can be found in Section 9.

8.2 Laboratory Procedures/Evaluations

8.2.1 Clinical Laboratory Evaluations

Protocol-mandated clinical screening and safety laboratory tests will be conducted in real time by laboratories that are properly accredited. These tests include:

- Biochemistry: Sodium, Potassium, creatinine, alkaline Phosphatase, total bilirubin, alanine aminotransferase (ALT), albumin, aspartate aminotransferase (AST), Creatinine Phosphokinase (CPK), Glucose
- Hematology: hemoglobin, white blood cells with differential count, platelet count
- Serology: HbSAg, anti-HCV, and HIV
- Blood typing
- Pregnancy test: Serum β-HCG

Screening labs may be repeated once if certain values are outside the acceptable ranges and if the value does not increase the risk to the subject in the opinion of the clinical investigator. If a subject with repeated Grade 1 abnormal lab values judged not clinically significant by the PI, with the concurrence of the Sponsor's Medical Officer, she/he will remain eligible for enrollment into the study.

Additional blood specimens (2 mL) will be collected for iOWH032 pK analysis Day 1 and Day 3 of the study.

8.2.2 Stool Specimen Preparation, Handling, and Storage

Stool Culture: All loose stools will be collected, graded, and weighed while volunteers are in the Clinical Research Unit. Quantitative cultures will be performed on the first two stool samples of each 24-hour period prior to the initiation of antibiotics. Stool specimens will be inoculated onto thiosulfate citrate bile salts sucrose (TCBS) agar plates either directly or after overnight incubation enrichment in alkaline peptone water before plating onto TCBS agar. Up to 2 stools daily will be cultured to determine the number of organisms per gram of stool. A rectal swab will be obtained if no stool was passed. Suspicious colonies will be agglutinated with polyvalent anti-O1 antisera.[6] Subsequent to the initiation of antibiotics, one stool sample for each 12-hour period (or a rectal swab if subject cannot produce stool) will be submitted for qualitative culture only. Three sequential negative stool cultures are required for discharge from the Clinical Research Unit.

8.2.3 Specimen Shipment

All biological specimens will be transported using packaging mandated by CFR 42 Part 72. All dangerous goods materials, including diagnostic specimens and infectious substances, must be transported according to instructions detailed in the International Air Transport Association Dangerous Goods Regulations. Biohazardous waste will be contained according to institutional, transportation/carrier, and all other applicable regulations.

8.3 Study Schedule

8.3.1 Screening (Outpatient Clinic Visit 1)

Potential volunteers may be screened for eligibility up to 85 days prior to enrollment. Interested potential participants will be recruited through standard procedures at Pharmaron and brought to the study site for screening and consenting.

Pharmaron research staff will provide a detailed description of all aspects of the study, including the rationale and background, the public health significance, the procedures and schedule of visits, and a detailed discussion of the risks and the presence or absence of benefit to them, as appropriate.

After the informed consent form is signed, the participant is interviewed one-on-one by a member of the study team to discuss the study. A brief written examination is administered to assess the volunteer's comprehension of the study (i.e., *Comprehension Assessment Tool*). If this quiz is passed (≥70% correct answers), the research staff complete the medical history and concomitant medication forms, draw blood for eligibility testing, and obtain an electrocardiogram (ECG). The participant also meets the principal investigator, or designee, and has a physical examination to complete the eligibility screening. If the participant has passed all the eligibility criteria, he/she is then invited to proceed in the study and is given an appointment for the next visit. The screening procedures may be completed in a single day or on multiple days, as long as the screening procedure dates do not exceed 85 days prior to challenge.

It is recognized that volunteer studies must be carried out in an environment where no coercion is applied, and where volunteers can be adequately informed of the purpose, nature, procedures, risks and hazards of the study. To assess and document comprehension of the material presented, each participant must pass a written quiz (score 70% or higher) containing approximately 20 multiple choice and true/false questions covering all aspects of the study including the purpose, procedures, risks, benefits and pertinent microbiology. Incorrect answers will be reviewed with the participant. If the participant scores at least 50% the quiz may be retaken once, after further education and a review of the consent form. The *Comprehension Assessment Tool* is dated and signed by the participant and by a research staff member and made part of the permanent record.

Another important feature of the consent process is the repeated demonstration of both initiative and reliability by the prospective participant. There are multiple opportunities for the participant to decline to proceed further in the process. This deliberate education and screening process contributes to the informed nature of the participant's consent. The process also increases the likelihood that the participant will be committed to completing the entire inpatient containment portion of this study. Prospective participants will be carefully screened to ensure that they are in good physical and mental health.

The screening procedures include:

- Signed, witnessed informed consent
- Administration of the study Comprehension Assessment Tool
- Obtain vital signs (oral temperature, blood pressure, pulse, height, and weight)
- Collection of medical history
- Collection of concomitant medication history
- Perform physical examination, to be performed by a study PI or designee
- Perform 12-lead electrocardiogram (ECG); the interpretation of screening ECG eligibility is defined in *Appendix B*.

- Obtain the following screening laboratory studies (~18 mL blood); the acceptable values for the screening laboratory tests are defined in *Appendix B*:
    - Complete Blood Count (CBC) with differential and platelet count for the evaluation of WBC, ANC, Hemoglobin, and Platelets
    - Sodium, Potassium, Creatinine, Albumin, AST (SGOT), ALT (SGPT), Alkaline Phosphatase, and Total Bilirubin, CPK, Glucose
    - Blood Type (e.g., A, B, or O)
    - Urinalysis for the evaluation of glucose and protein
    - Pregnancy test (serum β-HCG)
    - HIV antibody
    - Hepatitis B surface antigen
    - Hepatitis C antibody

Note: Since intravenous fluids is a part of the planned management of severe diarrhea, if more than 3 attempts at venipuncture are required for obtaining screening labs, then we will consider that person ineligible on the basis of poor venous access.

8.3.2 Inpatient Containment Period

Study participants determined to be eligible will be scheduled for admission to the inpatient containment unit (Clinical Research Unit). The anticipated duration of the inpatient stay is 11 days, participant to completion of discharge criteria, described below.

8.3.3 Acclimatization (1 day prior to challenge, Day -1)

Study participants will start their inpatient stay 1 day prior to challenge for acclimatization, during which we educate and familiarize each participant with the protocol-required procedures (e.g., stool handling), hygiene practices, and the "Rules and Procedures" to be followed while in the inpatient unit. In addition, during the acclimatization period, we monitor behavior, person-to-person interactions, mood, etc. to assess each study participant for any behavior or attitudes, which might not be appropriate for an inpatient containment study (i.e., combativeness, anti-social behavior, anger outbursts, destruction of property, etc.). Any evidence that a participant may demonstrate which might pose a safety risk to themselves, other participants, or staff could be cause for ineligibility for challenge and the remainder of the inpatient stay. Refusal to comply with protocol-required procedures, adherence to hygiene practices, or repetitive breaking of the "Rules and Procedures" could also constitute ineligibility. This observation during the acclimatization period may be considered an imperfect and rather subjective method, but we have not identified any other good alternate objective measures that substitute for this direct observation procedure. Any participant who is deemed ineligible will be discharged, prior to challenge.

Baseline Clinical Safety Laboratory (~10 mL) will be obtained prior to challenge to include:
- Complete Blood Count (CBC) with differential and platelet count for the evaluation of WBC, ANC, Hemoglobin, and Platelets
- Sodium, Potassium, Creatinine, AST (SGOT), ALT (SGPT), Albumin, Alkaline Phosphatase, and Total Bilirubin, CPK, Glucose

A serum pregnancy test (must be negative) will be performed for all women. A pre-challenge stool sample will be collected as a baseline, however if a subject fails to produce a screening stool sample it will not be considered a protocol deviation.

All participants that continue to demonstrate eligibility and provide continuing consent will fast (defined by nothing by mouth except for water) for 90 minutes before ingestion of the challenge inoculum.

8.3.4 Challenge Day (Day 1)

On the morning of challenge, fasting participants will have baseline vitals (oral temperature, pulse, and blood pressure) recorded and a final eligibility confirmation will be completed prior to oral ingestion of the challenge inoculum.

Fasting eligible participants will drink 120 mL of sodium bicarbonate solution (~1.3% $NaHCO_3$); approximately 1 minute later, participants will ingest 10^6 cfu of the challenge strain suspended in 30 mL of sodium bicarbonate solution (~1.3% $NaHCO_3$). Participants will have nothing by mouth, except water, for 90 minutes before and following ingestion of the challenge inoculum.

All back-up study participants that are not challenged will be discharged from the inpatient ward upon the completion of the target number of challenges. Blinded randomization will occur for all participants that successfully complete the ingestion of challenge.

8.3.5 Post-Challenge Observation Period (Day 1 through discharge)

At the onset of symptoms or by 48 hours after ingesting challenge inoculum of 10^6 cfu of *V. cholerae* El Tor Inaba strain N16961, blinded study drug will be administered for 3 days every 8 hours to all study participants. After each dose of blinded study drug, the following tolerability will be assessed: nausea, abdominal discomfort and pain, occurrence of abdominal cramps, occurrence of headache, and occurrence of malaise, anorexia, pollakiuria, micturition urgency, sinus tachycardia, increased alertness ; an attribution must be determined as related to study drug, cholera, or an alternate etiology (unrelated to study).

Following ingestion of the challenge inoculum, the following procedures will be performed:

- All stools will be graded for consistency (Grade 1-5) and any diarrheal stool (Grade 3 or higher) will be weighed. We assume a 1:1 weight per volume conversion for diarrheal stools.
- Any vomitus will also be collected and weighed.
- Vital signs will be measured at least 3 times daily (approximately every 8 hours). On Day 1 including the pre-challenge vitals assessment, there will be 4 vital signs assessments (1x pre-challenge and 3x post-challenge). Vital signs will also be measured every 4 hours when a participant has a fever ≥ 39˚C (102.1˚F) and diarrhea, until the participant has 2 consecutive temperatures of ≤ 38˚C (100.4˚F) and cessation of diarrhea. (See Section 9 for detailed procedures for the management of illness)
- During the first 7 days following challenge, the highest grade of an adverse symptom over that day will be assessed. The documentation of the previous day's highest grade of an adverse symptom (prior 24 hours) will include an attribution: study drug, cholera, or an alternate etiology (unrelated to study). The anticipated subjective adverse symptoms include abdominal discomfort and pain, nausea, abdominal cramps, malaise, headache, and anorexia. These adverse symptoms will be graded according to the scales shown in *Appendix E.*
- For each of the first 7 days, the maximum temperature, total diarrheal stool volume, number of diarrheal stools, total vomitus volume, and number of vomiting episodes will be calculated.
- Serum samples will be collected 7±1 hour after the first and the last dose of the iOWH032 for pK analysis

The management of clinical illness is described in Section 9.0 and Manual of Operating Procedures (MOP); including: criteria for oral and/or intravenous rehydration, necessity of increased frequency of vital signs monitoring, unscheduled physical examinations, obtaining clinical safety labs, and the initiation of antibiotic or anti-pyretic therapy.

8.3.6 Discharge Criteria

Participants will not be allowed to leave the Clinical Research Unit until they demonstrate that they are no longer infectious and cannot present a hazard to the community. Therefore, each participant must meet all the following criteria to be discharged:

- The participant has at least three negative sequential stool cultures (separated by ~12 hours apart each) from which *V. cholerae* does not grow
- The absence of moderate or higher grade objective reactogenicity (diarrhea, fever, and vomiting) for at least 12 hours prior to discharge
- The participant has completed a 3-day course of antibiotic therapy, which may include
    - Ciprofloxacin 500 mg, twice daily
    - Azithromycin 500 mg, once daily (for those persons who are allergic to fluoroquinolones)
    - Doxycycline 300 mg, once daily (for those persons who are allergic to fluoroquinolones)

8.3.7 Early Termination/Early Discharge

Participants that desire to withdraw from the study or are withdrawn by the PI (e.g., due to continuing non-compliance or safety concerns) during the inpatient period will be given instructions on appropriate follow up. If a participant desires to leave the inpatient containment unit early, we will need to ensure that the participant is not infectious to the community. Therefore, we plan to administer either Ciprofloxacin 1000 mg or Azithromycin 1000 mg, as a directly observed oral dose prior to discharge—selection of antimicrobial will be based on potential allergies to medications and the subject must have a negative stool culture before being allowed to leave. We will provide medication and give instructions for 2 additional days of the respective antimicrobial, for self-administration. A document of early termination/withdrawal will be completed by the study staff and co-signed by the participant. The participant will not be compensated for any inpatient days that are not completed, including the day of early termination.

8.4 Outpatient Clinic Visit 2 (Day 15 ±1)

Participants will be scheduled for an outpatient visit shortly after discharge, approximately 2 weeks after challenge. During this visit we will assess the emergence of adverse events and for any changes to medical history and concomitant medications. 10 mL blood will be drawn for safety labs.

8.5 Outpatient Clinic Visit 3 (Day 29 ±2)

Participants will be scheduled for an outpatient visit approximately 4 weeks after challenge. During this visit we will assess the emergence of adverse events and for any changes to medical history and concomitant medications. This is the last clinic visit scheduled.

8.6 Final Follow-up – Visit 4 (Day 180 ±14)

A final follow up visit will be completed by phone. This visit will only be for the collection of serious adverse events.

8.7 Unscheduled Visit(s)

Participants that experience any serious or severe adverse effects or experience an event of concern can be scheduled for an outpatient visit to have further evaluation. If an unscheduled visit occurs, a member of the clinical study team (PI, sub-investigator, nurse coordinator, or clinical nurse) will interview and evaluate the participant to determine the cause of the visit and provide care as needed.

8.8 Concomitant Medications, Treatments, and Procedures

Drugs used during the study will be reported in the Concomitant Medication CRF, including both prescription, non-prescription, and over-the-counter medication.

8.9 Prohibited Medications, Treatments, and Procedures

Treatment with drug listed in Appendix F and antimicrobial agents other than the ones that will be used for the treatment of Cholera diarrhea will not be permitted during the inpatient period unless discussed and approved by the PATH Medical Officer and PI.

9 MANAGEMENT OF EXPECTED CHOLERA ILLNESS

9.1 Clinical Evaluation

Subsequent to cholera challenge, participants will remain on the ward for approximately 10-11 days. Vital signs and oral temperature will be measured at least every 8 ±1 hours by staff nurses who remain on the ward 24h a day. Participants will be interviewed daily by a study physician to determine the occurrence of illness signs and symptoms (e.g., anorexia, malaise, abdominal cramps, and headache) which occurred during the previous study day; these data will be recorded on a standardized form and graded in severity according to Appendix E. A focused physical examination may be performed at the discretion of the physician according to the nature of a participant's complaint.

9.2 Measurement of Diarrhea and Vomitus

Since diarrhea is anticipated to be a common occurrence, all participants will be expected to collect every stool that is passed, from the time of challenge until discharge. Participants will be instructed to use a plastic stool collection basin, commonly called a "hat". All stools will be graded by the study staff. The grading of stool is based on consistency and the definition of diarrhea is a grade 3 or higher stool, as follows:

- Grade 1 – well formed (normal stool, does not take the shape of the container)
- Grade 2 – soft (normal stool, does not take the shape of the container)
- Grade 3 – thick liquid (diarrhea, takes the shape of the container readily)
- Grade 4 – opaque watery diarrhea
- Grade 5 – rice water diarrhea (clear watery)

Any Grade 3 or higher stool (diarrheal stool) must be weighed, to estimate the volume of fluid loss (assume ~1 g diarrheal stool = 1 mL of fluid lost).

Similarly, any episodes of vomiting should be collected in either a stool "hat" or a plastic "kidney" basin, provided. If a vomiting or diarrhea episode is not able to be collected in a basin (e.g., the participant has an "accident" while sleeping or before they are able to reach a toilet), then the volume of the output will be estimated.

9.3 Management of Fluid Loss

Oral Rehydration Solution (ORS) will be offered as the primary means of management of fluid loss. Participants who develop diarrheal stools (Grade 3 or higher) during the inpatient observation will be required to ingest standard WHO Oral Rehydration Solution (ORS) at 1.5 times the stool volume. Vomitus will be replaced with ORS in equal amounts, 1:1 ratio. At the discretion of the investigator, additional ORS may be administered. ORS will be prepared according to the manufacturer's package insert (Jianas Bros ORS). Unused ORS should be discarded 24 hours after preparation.

For the duration of diarrhea, the participant will be requested to provide a urine specimen from every void that they experience; the specimen will be tested for urine specific gravity. In the event that intravenous fluids are required, serum electrolytes (Sodium, Potassium, Chloride, Bicarbonate), BUN, and creatinine will be measured.

A physician is available by telephone or beeper at all times. Nurses will notify the on-call physician if any of the following occurs in a participant who is experiencing diarrhea and/or vomiting:

- Syncope
- Complaint of dizziness or lightheadedness or established orthostatic hypotension
- Urine specific gravity > 1.025
- > 500 mL behind in ORS replacement
- Vomiting of ≥ 500 mL once or total volume within the past 4 hours
- High fever ≥ 39°C (102.1°F)
- Severe headache, severe malaise, or severe abdominal pain
- Participant has a complaint for which he/she requests treatment
- Any other clinical situation that concerns the nurse

If a participant develops severe watery diarrhea or persistent vomiting and cannot maintain full hydration by oral means, according to the criteria on section 9.5, IV fluid replacement will be administered.

9.4 Frequency of Vital Signs Assessment

Vital signs (blood pressure, pulse, and oral temperature) will be measured approximately every 8±1 hours, unless more frequent monitoring is needed. Once a participant has passed a diarrheal stool (Grade 3 or higher), vital signs will be measured every 4 hours until the participant passes a Grade 1 or 2 stool or 24 hours have passed since the last Grade 3 – 5 stool, whichever comes first.

Vital signs will also be measured every 4 hours when a participant has a fever ≥ 39°C (102.1°F), until the participant has 2 consecutive temperatures of ≤ 38°C (100.4°F).

Any participant that complains of dizziness or lightheadedness upon standing will have orthostatic blood pressures assessed—BP after supine for ~5 minutes and BP after standing ~2-3 minutes. Orthostatic hypotension is defined as a drop in systolic BP > 20 mmHg or in diastolic BP > 10 mmHg.

9.5 Fluid Therapy/Hydration

Intravenous fluids (Lactated Ringers solution) will be administered to participants with diarrhea who meet any of the following criteria:

- Orthostatic hypotension
- Urine specific gravity > 1.025
- No urine output for ≥ 8 hours
- > 1000 mL deficit in ORS replacement
- At the investigator's discretion, based on clinical evaluation or on a participant's difficulty in keeping up with ongoing diarrheal losses by oral rehydration alone

Intravenous therapy will continue until the above criteria are no longer satisfied, the participant is able to take fluid by mouth, and a study physician determines that it is no longer required.

9.6 Indications for Antibiotics

Antimicrobials will be administered immediately if a participant meets the criteria for severe cholera diarrhea, defined as ≥5 L (5 kg) of cumulative diarrheal stool. All other participants who have not

met the criteria for severe cholera diarrhea will receive antibiotics at approximately 4 days after the challenge. Antimicrobials will continue for the completion of a 3-day course. In rare and unexpected instances, the study investigator will be allowed to administer antimicrobials for alternate reasons—e.g., if in his/her judgment it is necessary to ensure the safety of the participant.

The administration of a defined course of antibiotics (which starts, at the latest, on the fourth day post-challenge) results in the rapid elimination of susceptible cholera organisms from the stool, such that it is extremely unlikely that the discharge criterion (for three sequential negative stool cultures, ~12 hours apart) will fail to be achieved by the tenth day post-challenge.

If cholera organisms continue to be grown from stool cultures after the completion of the 3-day course of antibiotics, then another course of antibiotics can be entertained. Study participants will continue to be required to reside in the Clinical Research Unit until all the discharge criteria are met. Participants will be compensated for the additional days on the Clinical Research Unit. This event is considered to be a very rare likelihood since failure to eradicate the cholera challenge strain never occurred during nearly 40 years of experience using the N16961 strain.

9.7 Indications for Other Concomitant Medications

Any concomitant medication, prescription or over-the-counter, will be evaluated for continuation during the inpatient setting. Any such medication will need to be discussed with and approved by the investigator prior to admission. The supply of such medication will be the responsibility of the participant and will be handed over to the research staff upon admission; daily administration will be recorded. Any un-declared prescription or over-the-counter medications that are discovered during the inpatient stay will constitute a violation of the ward “Rules and Procedures.”

Other medications may be administered during the study period as follows:

- Smoking is not allowed on the ward, but participants will be able to request a nicotine patch.
- An anti-pyretic and analgesic (i.e. acetaminophen) may be prescribed for severe headaches, other pains, or fevers (e.g., sustained temperatures of ≥ 39°C).
- At the investigator’s discretion (e.g., upon review of serum electrolyte results during severe diarrhea), oral potassium may be administered for repletion of electrolyte losses.
- Other medications, which are deemed necessary for the safety and welfare of the participant

The prescription of any medication must be ordered and signed by the investigator and each administration recorded.

10 ASSESSMENT OF SAFETY

The assessment of the safety of the study drug and challenge agent will be through the detection and documentation of adverse effects, both solicited adverse events (AEs) and unsolicited AEs, and/or clinically significant laboratory abnormalities, through 28 days post-challenge. Only the occurrence of serious adverse events (SAEs) will be reported between 28 days and 180 days post-challenge.

10.1 Specification of Safety Parameters

10.1.1 Definition of Adverse Event (AE)

An adverse event is any untoward medical occurrence in a participant after administration of the investigational products and that does not necessarily have a causal relationship with the

investigational products. An AE can therefore be any unfavorable and unintended sign (including abnormal laboratory findings), symptoms, physical examinations, or disease temporally associated with the use of the investigational products, whether or not related to the investigational products. This definition includes exacerbations of pre-existing conditions. Stable pre-existing conditions which do not change in nature or severity during the study are not considered AEs; however, these should be reported as part of the medical history. In certain instances, it may be not be possible to distinguish between relatedness due to study drug or cholera; in those cases an AE may be noted as related to both.

Any AEs that are reported after signing the study ICF up to the Day -1 inpatient ward admission will be reported as a part of the medical history.

Solicited AEs are pre-specified AEs that could potentially be in association with the study drug or cholera infection. Investigators will attempt to assign causality of solicited AEs to either the study drug, cholera infection, or an alternate etiology. A solicited AE is a predetermined event, identified in the Investigator's Brochure, which may reflect safety concerns related to the investigational product.

Unsolicited AEs are any AEs reported spontaneously by the participant, observed by the study personnel during study visits or those identified during review of medical records or source documents. Investigators will attempt to assign causality of unsolicited AEs to either the study drug, cholera infection, or an alternate etiology.

10.1.2 Definition of Serious Adverse Event (SAE)

A serious adverse event (SAE) is any adverse event that results in any of the following outcomes:

1. Death
2. A life-threatening event. Life-threatening events mean that the study participant was, in the opinion of the site PI or Sponsor, at risk of death at the time of the event; it does not refer to an event which hypothetically might have caused death if it were more severe.
3. Requires inpatient hospitalization or prolongation of existing hospitalization
4. Results in persistent or significant incapacity or substantial disruption of the ability to conduct normal life functions
5. Congenital abnormality or birth defect
6. Important medical event that may not result in one of the above outcomes but may jeopardize the health of the study participant and/or requires medical or surgical intervention to prevent one of the outcomes listed in the above definition of serious adverse event. Examples of such medical events include allergic bronchospasm requiring intensive treatment in an emergency room or at home, blood dyscrasias or convulsions that do not result in inpatient hospitalization, or the development of drug dependency or drug abuse.

All SAEs will be:

- recorded in the research record
- reported to the local IRB, per local IRB guidelines
- reviewed and evaluated by a study clinician, the PI and Independent Medical Monitor
- followed through resolution by a study clinician

All SAEs, whether related or unrelated, will be reported to the PATH medical officer within 24 hours of site awareness.

10.1.3 Definition of Unanticipated Problems (UP)

The Office of Human Research Protections (OHRP) considers unanticipated problems involving risks to participants or others to include, any incident, experience, or outcome that meets all of the following criteria:

- Unexpected in terms of nature, severity, or frequency given (a) the research procedures that are described in the protocol-related documents, such as the IRB-approved research protocol and informed consent document; and (b) the characteristics of the participant population being studied;
- Related or possibly related to participation in the research ("possibly related" means there is a reasonable possibility that the incident, experience, or outcome may have been caused by the procedures involved in the research); and
- Suggests that the research places participants or others at a greater risk of harm (including physical, psychological, economic, or social harm) than was previously known or recognized.

This study will use the OHRP definition of UP.

10.2 Classification of an Adverse Event

10.2.1 Severity of Event

Severity of Adverse Events: All AEs will be assessed by the clinician using a protocol-defined grading system in Appendix E. For events not included in the protocol-defined grading system, the following guidelines will be used to quantify severity:

Mild: events require minimal or no treatment and do not interfere with the participant's daily activities.

Moderate: events result in a low level of inconvenience or concern with the therapeutic measures. Moderate events may cause some interference with functioning.

Severe: events interrupt a participant's usual daily activity and may require systemic drug therapy or other treatment. Severe events are usually incapacitating.

Life threatening: any adverse drug experience that places the participant, in the view of the investigator, at immediate risk of death from the reaction as it occurred, i.e., it does not include a reaction that had it occurred in a more severe form, might have caused death.

Changes in the severity of an AE should be documented to allow an assessment of the duration of the event at each level of intensity to be performed. Adverse events characterized as intermittent require documentation of onset and duration of each episode.

10.2.2 Relationship to Study Agent or Cholera Infection

Relationship (causality or attribution) of all AEs to the study drug, known cholera infection, or to an alternate etiology (unrelated to the study) is part of the documentation process, but it is not a factor in determining what is (or is not) reported in the study. If there is any doubt as to whether a clinical observation is an AE, the event should be reported.

The PI must assign a relationship of each AE to the receipt of the investigational product. The investigator will use clinical judgment in conjunction with the assessment of a plausible biologic mechanism, a temporal relationship between the onset of the event in relation to receipt of the

investigational product, and identification of possible alternate etiologies including underlying disease, concurrent illness, or concomitant medications.

All AEs must have their relationship assessed using one of three terms: related to study drug, related to cholera, or not related (an alternate etiology can be identified and is likely or feasible). To help assess, the following guidelines are used.

Related to study drug (i.e., iOWH032 or placebo) – There is a reasonable possibility (perhaps due to the timing of onset of the symptoms) that the study drug caused the adverse event. Reasonable possibility means that there is evidence to suggest a causal relationship between the study drug and the adverse event.

Related to cholera - There is a reasonable possibility that the cholera infection caused the adverse event. Reasonable possibility means that there is evidence to suggest a causal relationship between the cholera illness and the adverse event.

Not Related – There is not a reasonable possibility that the event is related to either the study drug (i.e., iOWH032 or placebo) or cholera and there is a reasonable alternate etiology. Thus, allergic reactions to the protocol-required antimicrobial will be classified as “not related” and the alternate etiology will be designated as a reaction to the antimicrobial.

10.2.3 Expectedness

The study investigator will be responsible for determining whether an AE is expected or unexpected. An AE will be considered unexpected if the nature, severity, or frequency of the event is not consistent with the risk information previously described for the study agent in the Investigator’s Brochure.

10.3 Time Period and Frequency for Event Assessment and Follow-up

The occurrence of an AE or SAE may come to the attention of study personnel during study visits or upon review by a study monitor. All AEs including local and systemic reactions not meeting the criteria for SAEs will be captured on the appropriate CRF. Information to be collected includes event description, time of onset, clinician’s assessment of severity, relationship to study product, and time of resolution/stabilization of the event. All AEs occurring through 28 days post-challenge, must be documented appropriately regardless of relationship. All AEs will be followed to adequate resolution.

Any medical condition that is present at the time that the participant is screened will be considered as baseline and not reported as an AE. However, if the study participant’s condition deteriorates at any time during the study, it will be recorded as an AE. UPs will be recorded in the data collection system throughout the study.

Changes in the severity of an AE will be documented to allow an assessment of the duration of the event at each level of severity to be performed. AEs characterized as intermittent require documentation of onset and duration of each episode.

The PI will record all reportable events with start dates occurring any time after the challenge is obtained until 28 (for unsolicited AEs) or 180 days (for SAEs) after the last day of study participation. At each study visit, the investigator will inquire about the occurrence of AE/SAEs since the last visit. Events will be followed for outcome information until resolution or stabilization.

10.4 Reporting Procedures

10.4.1 Adverse Event Reporting

To improve the quality and precision of AE data, the investigator should observe the following guidelines:

- AEs must be graded, assessed for severity and causality, and reviewed by a site investigator.
- Whenever possible, use recognized medical terms when reporting AEs and avoid the use of colloquialisms or abbreviations.
- If known, report the diagnosis (i.e., syndrome or disease) rather than component symptoms, signs or laboratory values (e.g., report congestive heart failure rather than dyspnea, rales, and cyanosis); however, symptoms or signs that are considered unrelated to an observed syndrome or disease should be reported as individual AEs (e.g., if congestive heart failure and severe headache are observed at the same time, each event should be recorded as an individual AE).
- AEs occurring secondary to other events (e.g., sequelae) should be identified by the primary cause. A 'primary' AE, if clearly identifiable, generally represents the most accurate clinical term to report. For example: orthostatic hypotension → fainting and fall to floor → head trauma → neck pain; the primary AE is orthostatic hypotension, which is what should be reported. If a primary SAE is reported, events occurring secondary to the primary event should be described in the narrative description of the case.
- Death is an outcome of an event. The event that resulted in the death should be reported as the SAE.
- For hospitalizations for surgical or diagnostic procedures, the illness leading to the surgical or diagnostic procedure should be recorded as the SAE, not the procedure itself. The procedure should be captured in the case narrative as part of the action taken in response to the illness.
- Elective surgical or diagnostic procedures with or without hospitalizations (e.g., circumcision or elective abortion of a pregnancy) will not be recorded as an AE. The procedure should be captured in the case narrative as part of medical history.

10.4.2 Serious Adverse Event Reporting

The study clinician will complete a SAE Form within the following timelines:

- All SAEs, whether related or unrelated, will be recorded on the SAE Form and submitted to the PATH Medical Officer within 24 hours of site awareness. See **Section 1, Key Roles** for contact information.

Even if the investigator does not have complete information regarding an SAE, the SAE should still be reported within 24 hours and once additional relevant information is received, the report should be updated within 24 hours. The investigator should always provide an assessment of causality at the time of the initial report, even though this assessment may subsequently change based on the follow-up information received. After receipt of the initial report, PATH Medical Officer will contact the investigator if it is necessary to obtain further information for assessment of the event.

All SAEs will be followed until satisfactory resolution or until the site investigator deems the event to be chronic or the adherence to be stable. Other supporting documentation of the event may be requested by PATH and should be provided as soon as possible. PATH will be responsible for notifying FDA of any unexpected fatal or life-threatening suspected adverse reaction as soon as possible but in no case later than 7 calendar days after the PATH's initial receipt of the information.

10.4.3 Unanticipated Problem Reporting

Incidents or events that meet the OHRP criteria for UPs require the creation and completion of an UP report form. It is the site investigator's responsibility to report UPs to Advarra and to PATH. The UP report will include the following information:

- Protocol identifying information: protocol title and number, PI's name, and the Advarra Protocol number;
- A detailed description of the event, incident, experience, or outcome;
- An explanation of the basis for determining that the event, incident, experience, or outcome represents an UP;
- A description of any changes to the protocol or other corrective actions that have been taken or are proposed in response to the UP.

To satisfy the requirement for prompt reporting, UPs will be reported using the following timeline:

- UPs that are SAEs will be reported to the IRB and to PATH within 24 hours of the investigator becoming aware of the event.
- Any other UP will be reported to the IRB and to the study sponsor within 72 hours of the investigator becoming aware of the problem.
- All UPs should be reported to appropriate institutional officials (as required by an institution's written reporting procedures), the supporting agency head (or designee), and OHRP within 96 hours of the IRB's receipt of the report of the problem from the investigator.

10.4.4 Reporting of Pregnancy

iOWH032 has not been shown to be safe during pregnancy, so pregnant women may not participate in the study and women with child-bearing potential must agree to use effective methods of birth control 4 weeks prior to challenge and continue throughout the study. Although every effort will be made to counsel women about the pregnancy precautions related to study participation, if a woman becomes pregnant during the treatment phase of the trial, efforts will be made to obtain permission to continue to follow the participant through the pregnancy and to document the outcome of the pregnancy. The pregnancy and its outcome will be documented, even if birth occurs after the scheduled end of the study for the participant. A pregnancy in a participant is not in and of itself an AE. A pregnancy is reported as an AE or SAE only when there is suspicion that the investigational product may have interfered with the effectiveness of contraception or there was a serious complication in the pregnancy including a spontaneous abortion or an elective termination for medical rationale. The participant will be asked to provide serial follow-ups on the status of her pregnancy as well as provide health information on her infant following delivery. The participant will be asked to provide copies of clinic visits only if needed. The participants will receive no further study drug but may continue to receive antimicrobials if needed. The participants will continue safety monitoring and all follow up visits as noted in the study schedule.

Principal investigator will notify study Independent Medical Monitor and PATH Medical Officer for participants that become pregnant during the study. Each pregnancy must be reported immediately (within 24 hours of identification) by e-mail or fax to the sponsor.

10.5 Study Halting Rules

The study is planned to have each cohort of participants to be challenged at the same time. The greatest potential for risk is with the intentional cholera infection and with the dosing of the study drug (during the first 5 days post-challenge). Should one of the study halting criteria be met, all participants of that cohort (and the following cohort, if applicable) will continue to be actively monitored and managed for their clinical illness (if during the inpatient period) and continued to be followed for safety information (if during the outpatient period).

If any of the following events occurs during the study, administration of IP will be halted to all subjects in the study until a thorough joint review of the safety concern is undertaken and completed by the Safety Review Committee (SRC) and PI. The halting rules refer to suspected adverse reactions and will be triggered automatically if any of the events described below are met during the conduct of the study:

- One or more participants experience a serious adverse event related to the study product.
- Two or more participants experience the same severe (Grade 3) AE of the same organ class (systemic toxicity, or clinical laboratory tests or vital signs) deemed related to the study product within seven days following the study drug administration
- One serious and unexpected, solicited or unsolicited, suspected or confirmed adverse reaction evaluated by the PI, Independent Medical Monitor, and PATH Medical Officer and determined to be an unacceptable risk to the health and safety of other investigational product recipients

10.6 Safety Oversight

Safety monitoring/oversight will be performed by a Safety Review Committee composed of three voting members (including two independent medical experts in the field): a chair, co-chair, and an independent medical monitor. The primary responsibility of the SRC is to monitor participant safety. The responsibilities of the SRC and safety oversight activities are described in the protocol and will be clearly outlined in the SRC Charter. The SRC considers study-specific data as well as relevant background information about the disease, test article, and target population under study. The SRC will review challenge data shortly after completion of the inpatient phase and will review the interim analysis after Cohort 1 has completed study activities before proceeding to the second cohort. One of the SRC members, a physician, will be a designated Independent Medical Monitor. The SRC will also be empowered to suspend the study, recommend amendments to the protocol, recommend proceeding with the next Cohort, and/or to request further information for their Additionally, SRC will review the safety data only post enrollment of Cohort 2.

The Independent Medical Monitor will be responsible for regularly reviewing the cumulative safety data, including a review of safety laboratory test results and adverse event reporting, should there be a halt in the study or at the request of the SRC. The SRC is empowered with halting the study at any time. Should there be a halt in the study, no further challenges or study drug dosing will be performed; all enrolled participants will continue to be followed; for participants that are in the Clinical Research Unit, the management of diarrhea or other symptoms will continue until resolution. Antimicrobials will be administered immediately if a participant meets the criteria for severe cholera diarrhea. Further drug administration, in accordance with the protocol, may be resumed with the concurrence of the SRC, PATH Medical Officer and the PI. The sponsor will make the final determination as to whether the study will be stopped permanently. If the study is permanently stopped, participants will be followed until their next scheduled study visit for the safety assessments or until AE/SAE resolution, whichever is longer.

11 CLINICAL MONITORING

Site monitoring is conducted to ensure that the rights and well-being of human participants are protected, that the reported trial data are accurate, complete, and verifiable, and that the conduct of the trial is in compliance with the currently approved protocol/amendment(s), with GCP, and with applicable regulatory requirement(s). Monitoring refers to the methods used by sponsors of investigational studies, or Contract Research Organizations (CROs) delegated site monitoring responsibilities, to oversee the conduct of, and reporting of data from, clinical investigations. Site monitoring includes ensuring appropriate clinical investigator supervision of study site staff and third party contractors.

- Monitoring for this study will be performed by The Total Approach.
- Monitoring visits will occur on-site throughout the study and will include 100% data verification of primary endpoint data (such as clinical signs and fluid output data) and excretion data. Targeted 10% data verification will be conducted for vital signs and other non-critical data.
- PATH Medical Officer will be provided copies of monitoring reports within 14 days of visit.
- Details of clinical site monitoring are documented in a Clinical Monitoring Plan. The Clinical Monitoring Plan describes in detail who will conduct the monitoring, at what frequency monitoring will be done, at what level of detail monitoring will be performed, and the distribution of monitoring reports.
- Independent internal audits will also be conducted, as described in the Pharmaron Quality Management Plan. The results of the internal audit are reviewed with the PI, coordinator, and Clinical Research Manager; Corrective Action Plans are formulated in response to any issue.

12 STATISTICAL CONSIDERATIONS

12.1 Statistical and Analytical Plans

The statistical analysis will be conducted following the principles as specified in the International Conference on Harmonization (ICH) Topic E9 (ICH, 1998). The final statistical analyses will be performed after database lock and final unblinding of the data.

Full details of the statistical analysis and methods will be documented in a statistical analysis plan (SAP) which will be finalized prior the interim analysis. The SAP will follow the general guidance of this protocol, and will further describe the selection of participants to be included in the analyses, detailed statistical and analytical plans for both the interim and final analyses, and procedures for accounting for missing, unused, and spurious data.

12.2 Statistical Hypotheses

The null (H_0) and alternative (H_1) hypotheses of the primary efficacy endpoint analysis are as follows:

H_0: The diarrheal stool output rate from participants treated with iOWH032 is not lower than that of participants treated with placebo.

H_1: The diarrheal stool output rate from participants treated with iOWH032 is lower than that of participants treated with placebo.

The diarrheal stool output rate is defined as the total volume of diarrheal stools (mL, Grade 3 and above) divided by the number of hours between initiation of study product dosing and initiation of antimicrobial therapy. The hypothesis is one-sided to permit the interim analysis and clear description of efficacy/futility boundaries. See Section 12.5.8 for a description of the one-sided alpha levels to be used for testing the primary endpoint.

This hypothesis test will be supplemented with two-sided confidence intervals for the difference of medians, as described below. Secondary efficacy endpoints regarding AUC of diarrheal stool volume as well as quantitative culture of cholera organisms (defined by AUC and peak shedding) from stool samples will be analysed with similar methods.

The null (H_0) and alternative (H_1) hypotheses for the secondary analysis of the proportion of participants with moderate or severe diarrhea are as follows:

H_0: iOWH032 and placebo do not differ in the proportion of participants that develop moderate or severe diarrhea

H_1: iOWH032 and placebo differ in the proportion of participants that develop with moderate or severe diarrhea

12.3 Timing of Analyses

When all Cohort 1 participants have completed the inpatient containment period and primary efficacy endpoint data are available, an interim analysis of the primary efficacy endpoint and accompanying safety data will be initiated. The study is designed with binding efficacy and futility thresholds, described in Section 12.5.8. Cohort 2 will be enrolled following this interim analysis if neither overwhelming efficacy, nor evidence of futility is indicated, with the final analysis resulting from the combined data from both cohorts. This process will include the review of the safety data by the SRC and determination of lack of major safety signal.

Additionally, topline results will be conducted on the after the efficacy data data from Cohorts 1 and 2 have been collected (i.e. including data prior to Visit 2). These topline results will include analysis of the primary efficacy endpoint, as well as the analyses of the secondary endpoints of proportion of participants with MSD, attack rate of any diarrhea, AUC of stool volume, duration of episode (time to first formed stool), and total number of loose stools conducted on the mITT population. In addition, subject disposition and demographic and baseline characteristics tables will be provided. The topline results will be unblinded only to the treatment-level and not to the individual subject. To avoid identification of individual subjects, some summary statistics such as minimum and maximum will not be presented in the summary tables.

The final statistical analyses will be performed after database lock and final unblinding of the data.

12.4 Analysis Datasets

Assignment of participants to analysis populations will be completed prior to unblinding, for each cohort.

For purposes of analysis, the following populations are defined:

Population	Description
Enrolled	All participants who sign the ICF.

Population	Description
Intent to Treat (ITT)	All participants who are randomized to study treatment Participants will be analysed according to their randomized treatment.
Modified ITT (mITT)	The subset of ITT who receive at least one dose of study drug. Any participant displaying no indication of cholera infection (no diarrheal stool output of grade 3 or higher) within 48 hours of challenge will be removed from the mITT population, prior to unblinding of data.
Per Protocol Population	The subset of the mITT consisting of those participants who had no major protocol deviations and received all doses of assigned study drug.<br>All protocol deviations will be assessed and documented on a case-by-case basis before the database lock, and deviations considered having a serious impact on the efficacy results will lead to the relevant participant being excluded from the per-protocol population. Before database lock, potential participant exclusions from per-protocol population will be reviewed by the Sponsor and documented.
Safety	The Safety Analysis Set includes all participants who received any study treatment. Safety analyses and demographic/baseline characteristic summaries will be based on the safety population, according to treatment received.

12.5 Description of Statistical Methods

12.5.1 General Approach

This is a single-centre, double-blind, randomized, placebo-controlled trial comparing the efficacy and safety of iOWH032 to placebo after a cholera challenge in adult healthy volunteers.

SAS version 9.4 or greater will be used for analysis. For descriptive statistics, continuous variables will be summarised by the number of non-missing observations, mean, median, standard deviation, and minimum and maximum. Categorical variables will be summarised by presenting the frequency and percent. Unless otherwise specified, inference such as confidence interval construction will be conducted with two-tailed Type I error level $\alpha = 0.05$. No adjustment for multiple comparisons across endpoints will be conducted. All secondary endpoints will be considered as supportive evidence of efficacy and will be analysed without any procedures to account for multiple comparisons.

In general, nonparametric analyses or exact methods (e.g., Fisher's exact test) will be preferred for efficacy analyses, with confidence intervals for binary variables computed via the Clopper-Pearson exact method, and confidence intervals for continuous variables computed via the percentile bootstrap method, using n=10,000 replicates each.

Primary efficacy analysis will be stratified by blood type status (O vs Non-O) as described below. All efficacy endpoints will be summarized, by group and by group and O vs Non-O status, using daily and overall summaries, where appropriate. Exploratory analyses will consider stratification by cohort to account for potential differences in challenge inoculum between cohorts.

Analysis of diarrheal stool output rate, diarrheal stool volume, cholera-containing diarrheal stool volume, and density of cholera organisms will be supplemented with reverse cumulative distribution curves.

All data will be provided in listings, in addition to the summaries described below.

12.5.2 Analysis of the Primary Efficacy Endpoint(s)

The primary efficacy endpoint is "Diarrheal stool output rate", defined as the total volume of diarrheal stools (mL, Grade 3 and above) divided by the number of hours between initiation of study product dosing and initiation of antimicrobial therapy.

Statistical comparison of the primary endpoint between groups will be conducted with the stratified (by blood type group) version of the Wilcoxon rank-sum test, the Van Elteren test. If a one-sided statistical test rejects H_0 and the median is less for the iOWH032 group than for the placebo group, the study will have demonstrated that iOWH032 is superior. The hypothesis test will be supplemented by a two-sided confidence intervals for the difference in median diarrheal stool output rate, using the percentile bootstrap method, with n=10,000 replicates. See Section 12.5.8 for a description of the one-sided alpha levels to be used for testing the primary endpoint.

The principal analysis will be performed using the mITT population. If greater than 20% of subjects are excluded from the mITT due to onset of symptoms after 48 hours, the primary endpoint will be calculated again including patients with symptom onset after 48 hours. For subjects with symptom onset after 48 hours, the time between onset of symptoms and administration of antimicrobials will define the denominator used to calculate the diarrheal stool output rate. Supportive analysis will be performed using the per-protocol population.

Analysis of the primary endpoint will be conducted at an interim analysis upon completion of 24 participants, and again on the aggregate data after an additional 24 participants have completed the study. Methods defined in Section 12.5.8 will be used to control overall type I error rate at one-sided level alpha = 0.025.

12.5.3 Analysis of the Secondary Efficacy Endpoints

Secondary efficacy endpoints include a) the proportion of participants with moderate or severe diarrhea following cholera challenge defined as >3.0 L and >5.0 L of loose stools, as well as b) the attack rate of any diarrhea following cholera challenge defined as the number of participants with 2 or more loose stools (grade 3-5) totalling >200 mL within 48 hours or 1 loose (grade 3-5) stool > 300 mL.

Both endpoints will be computed as the proportion within each group satisfying the respective definitions, and will be accompanied by exact two-sided 95% confidence intervals. This will be supplemented with corresponding summaries within study arm and blood type group. Statistical inference for each endpoint will be conducted with the Cochran-Mantel-Haenszel test, stratified by blood type group.

If a two-sided- statistical test with Type I error rate of 0.05 rejects H_0 and the proportion of participants is less for the iOWH032 group than for the placebo group, the study will have demonstrated that iOWH032 is superior.

The AUC of diarrheal stool volume and cholera organisms will be computed via the trapezoidal rule. The secondary endpoints of AUC of cholera-containing diarrheal stool volume, AUC of cholera organisms, and peak shedding of cholera organisms (highest cfu counts for each participant) will be analysed in quantitative stool culture and will be summarized and analysed with methods similar to the primary efficacy endpoint, to be further defined in the SAP.

Cholera illness during the interval immediately following challenge and prior to initiation of antibiotics will be summarized by group and by blood type group (O vs non O) within group.

- Time (hours, post-challenge) to the last unformed stool (grade >=2, overall, as well as by-grade), will be summarized with Kaplan-Meier methods and compared with the log-rank test
- The total count of loose stools will be summarized as a continuous variable and compared separately for each blood type group with the Wilcoxon rank-sum test and jointly with the Van Elteren test, stratified by blood type group (O vs non O).
- The presence of fever or vomiting will each be summarized as categorical variables, with between-group inference conducted separately for each blood type group with Fisher’s exact test, and jointly via the Cochran-Mantel-Haenszel test, stratified by blood type group (O vs non O).

The principal analyses will be performed using the mITT population. Supportive analysis will be performed using the per-protocol population.

Detection of cholera in stool post-challenge will be summarized overall and by time point using categorical methods. Time to cessation of detectable cholera in stool (hours) will be analysed using Kaplan-Meier methods. Additional exploratory efficacy analyses may be identified, and should be described in SAP, prior to the interim analysis.

12.5.4 Safety Analyses

All safety analyses and demographic/baseline characteristic summaries will be based on the safety population, according to treatment received.

Adverse Events: The summary of AEs will be based on treatment-emergent AEs (TEAEs) defined as AEs which start or worsen following the start of challenge and up until the follow-up visit.

TEAE summaries will be based on system organ class and preferred term assigned to the event using the Medical Dictionary for Regulatory Activities (MedDRA). A summary table will be presented, by treatment group, for the numbers and percentages of participants with any TEAEs, related TEAEs, serious TEAEs, serious and related TEAEs, any severe TEAEs, and TEAEs leading to discontinuation from the study. TEAEs will also be summarized by preferred term/system organ class and severity by treatment group. This table will also be presented for TEAEs causally related to study treatment (defined as those described as possibly, probably or definitely related to study drug). Tables will also include an overall event count by treatment group. All subject-level percentages (solicited/unsolicited AEs, etc.) will be supplemented with two-sided 95% confidence intervals computed via the Clopper-Pearson method.

Solicited adverse events collected during the dosing phase will be summarized by causality and severity, using the maximum grade overall.

Clinical laboratory values will be graded per Appendix D, and abnormalities for each lab (and overall) will be summarized by maximum grade overall and per time point. A corresponding summary will be produced only for those considered to be clinically relevant.

Physical exam abnormalities will be summarized. Clinically relevant abnormal vital signs will be summarized. Abnormal screening ECG results will be summarized.

Continuous summaries of clinical lab values and vital signs, including change from baseline, will be computed by group and time point.

12.5.5 Adherence and Retention Analyses

Participants who prematurely discontinue from the study will not be included in analyses/summary statistics beyond the time of discontinuation.

Rates of discontinuation and reason for discontinuation will be summarized in data listings or summary tables.

12.5.6 Protocol Deviations

Protocol deviations will be summarized by group and listed, with classification into major/minor of participant-specific deviations completed on blinded data prior to database lock.

12.5.7 Baseline Descriptive Statistics

Demographic data, including blood type, age, gender, height, weight, BMI, and race/ethnicity will be summarized using descriptive statistics by treatment group. Continuous variables will be summarised by the number of non-missing observations, mean, median, standard deviation, and minimum and maximum. Categorical variables will be summarised by presenting the frequency and percent.

Prior/ongoing and new concomitant medications will be summarized (separately) by anatomical-therapeutic-chemical (ATC) class after coding with the WHO Drug dictionary.

12.5.8 Planned Interim Analyses

Once efficacy data are available from the completion of Cohort 1, an interim analysis of the primary efficacy endpoint will be conducted to guide the decision to move forward with Cohort 2. The interim analysis focusing on safety and efficacy of the iOWH032 will be partially blinded and will be shared with the SRC for a recommendation to the sponsor. Only the primary endpoint will be compared to the boundary values below for purposes of interim and final analysis.

The following boundary values will be used at the interim analysis:

- If the one-sided Van Elteren test of the superiority of the diarrheal stool output rate (iOWH032 vs placebo) yields a p-value less than **0.0051**, then the study product will be deemed superior to placebo, and the second cohort will not be enrolled
- If the one-sided Van Elteren test of the superiority of the diarrheal stool output rate (iOWH032 vs placebo) yields a p-value greater than **0.4585**, this will be considered evidence of futility (lack of demonstrated efficacy), and the second cohort will not be enrolled
- In all other cases, the 2nd cohort will be enrolled

Due to the nonzero probability of concluding efficacy (under alternative hypotheses) at the interim analysis, the alpha level (type I error rate) for the final analysis needs to be adjusted to maintain overall one-sided level alpha = 0.025. The threshold to define success on the primary efficacy endpoint at the final analysis is one-sided alpha = **0.0238**.

12.5.9 Multiple Comparison/Multiplicity

Adjustment for multiplicity for the group-sequential analysis of the primary efficacy endpoint is described in Section 12.5.8. All secondary endpoints will be considered as supportive evidence of efficacy and will be analysed without any procedures to account for multiple comparisons.

12.5.10 Exploratory Analyses

Additional exploratory analyses may be identified, and should be described in SAP, prior to the interim analysis.

12.5.11 Missing data

All reasonable measures will be taken to ensure minimal missing data. Despite this, some data are likely to be missing at the end of the study. No imputation is planned for any endpoints in this study, and all data will be considered to be missing completely at random. Should observations indicate this is not likely to be true for a given endpoint, additional sensitivity analyses may be conducted, such as multiple imputation, to assess the impact of the missing data.

12.6 Sample Size

This study is a Phase 2a study to determine the presence and magnitude of treatment effect of iOWH032 on prevention of cholera sequela after a cholera challenge. Data from placebo-vaccinated participants in a prior cholera challenge study conducted at the University of Maryland, including a mixture of type O and Non-O participants, demonstrated a mean total fluid output of 4448 ± 3178 mL over the post-challenge follow-up period[5]. Type O participants had a minimum total diarrheal output volume of approximately 500 mL and a maximum of approximately 11,200 mL, with a roughly uniform distribution across this span. For non-Type O participants, the minimum was approximately 600 mL and the maximum was approximately 7,800 mL. The daily rate of diarrheal stool output between challenge and initiation of antibiotic therapy estimated from these same data yield a roughly uniform distribution between 100 mL/day (both groups) and 2,200 mL/day (non-type O) or 3,000 mL/day (Type O). For sample size computation, it was assumed that:

- The placebo group in the present study would yield daily diarrheal stool rates uniformly distributed between 100 mL and 3,000 mL/day for Type O participants, and between 100 mL/day and 2,200 mL for non-Type O participants
- Treatment with iOWH032 would yield approximately a 50% reduction in daily diarrheal stool rate, with values uniformly distributed between 100 mL and ½ the upper limit for the placebo participants (1,500 mL/day and 1,100 mL/day for Type O and non-Type O participants, respectively)
- Approximately 40% of participants will be blood Type O, and will be equally distributed across study arm and study stage

This represents an assumption that all participants will respond to treatment, which is currently unknown. No prior data are available to inform the expected effect size, and the hypothesis selected here is expected to represent a feasible effect size for an impactful therapy. Randomization is stratified by blood type group (type O vs non-type O) to ensure balanced randomization, and

therefore a stratified analog of the Wilcoxon rank-sum test, the Van Elteren test, is employed for joint analysis across blood type group.

Participants are to be excluded from analysis if there is lack of evidence of cholera infection following challenge, defined as no diarrheal stool output of grade 3 or higher within 48 hours of challenge. Additionally, participants displaying rapid onset of severe cholera may have diarrheal stool output volume truncated due to early initiation of antimicrobial therapy. The use of a rate endpoint, as well as the nonparametric test for analysis is expected to yield a result that is insensitive to the influence of these observations, which are expected to be rare. The distribution of diarrheal stool output volume and rate is not well-characterized, and cannot be assumed to be normally-distributed based on prior data, further implying the need for a nonparametric analysis.

The primary efficacy endpoint is to be analysed at both an interim and a final analysis. Because of the multiplicity of testing and accompanying efficacy and futility thresholds at the interim analysis, a group-sequential framework is employed. Traditional methods for trial design are intractable in this case, due to the anticipated distribution of the endpoint and the use of stratified nonparametric testing for its evaluation. For this reason, a simulation-based framework was used to simulate data under both the null and alternative hypotheses. Power is computed as the proportion of simulations under the alternative hypothesis which yield a conclusion of superiority at either interim or, given that futility is not declared at interim, at the final analysis. Type I error is computed as the proportion of simulations under the null hypothesis which yield the same conclusion, erroneously. The simulation procedure was submitted to a constrained optimization routine that selected boundary values which ensure overall ≥90% power and type I error ≤0.025, simultaneously. Further simulations confirmed the adequacy of these boundaries to provide high power while maintaining nominal type I error rate. The boundary values that will be used at the interim analysis are:

- If the one-sided Van Elteren test of the superiority of the diarrheal stool output rate (iOWH032 vs placebo) yields a p-value less than **0.0051**, then the study product will be deemed superior to placebo, and the second cohort will not be enrolled
- If the one-sided Van Elteren test of the superiority of the diarrheal stool output rate (iOWH032 vs placebo) yields a p-value greater than **0.4585**, this will be considered evidence of futility (lack of demonstrated efficacy), and the second cohort will not be enrolled
- In all other cases, the 2nd cohort will be enrolled

Due to the nonzero probability of concluding efficacy at the interim analysis, the alpha level (type I error rate) for the final analysis needs to be adjusted to maintain overall one-sided level alpha = 0.025. The threshold to define success on the primary efficacy endpoint at the final analysis is one-sided alpha = **0.0238**. This hypothesis test will be supplemented by a two-sided 95% confidence interval for difference in median diarrheal stool output rate, using the percentile bootstrap method, with n=10,000 replicates.

With the abovementioned boundary values and sample size, the power decreases to 70% for a 40% reduction in daily diarrheal stool rate and to 30% a reduction of 25% in daily diarrheal stool rate. However, and effect size of 50% reduction is expected to represent a feasible effect size for an impactful therapy.

For the secondary endpoint of proportion of participants with moderate or severe diarrhea, with 24 participants per group, and assuming 40% of participants are Type O, adequate power is available to

detect odds ratios of moderate or severe diarrhea in iOWH032-treated participants vs placebo which are significantly less than 1, using a Cochran-Mantel-Haenszel test stratified by blood type group, with two-sided level alpha = 0.05. For example, if 67% (47%) of the type O (non-type O) control participants experience moderate or severe diarrhea, and only 20% (10%) of type O (non-type O) iOWH032-treated participants do (common odds ratio 0.123), then 80% power is available to detect a common odds ratio < 1. Only a single analysis will be conducted for efficacy endpoints other than the primary efficacy endpoint, upon collection of data from all participants included in the experiment.

12.7 Measures to Minimize Bias

12.7.1 Enrollment/ Randomization/ Masking Procedures

This parallel group study is randomized and double-blinded to minimise bias, and includes a placebo control arm to enable those effects related to dosing of randomized study treatment to be more readily identified. Participants will be randomized using a 1:1 ratio to iOWH032 or matching placebo. The randomization scheme will be generated by an independent statistician. The study site will receive one set of randomization envelopes to be stored and used in the study clinic. Clinic staff will assign these envelopes in sequential order, by envelope number, to eligible participants.

Section 12.7.3 details the procedures for breaking the study blind.

12.7.2 Evaluation of Success of Blinding

This study is designed as a randomized, double blind, placebo controlled, parallel group study. As such, participants will be allocated to either iOWH032 or matching placebo. No specific evaluation of the success of study blinding is planned. Any unblinding will be noted in case report forms.

12.7.3 Breaking the Study Blind/Participant code

In the event of a medical emergency, the site PI may require that the blind be broken for the subject experiencing the emergency when knowledge of the subject's treatment assignment may influence the subject's clinical care. Prior to unblinding, the site PI is encouraged (to the extent possible, without jeopardizing the subject's health) to contact PATH Medical Officer (or designee) to discuss the decision to break the blind. In case of medical emergency, PATH must be notified within 24 hours after breaking the blind. The date and reason that the blind was broken must be recorded in the source documentation and case report form, as applicable.

13 SOURCE DOCUMENTS AND ACCESS TO SOURCE DATA/DOCUMENTS

Source data are all information, original records of clinical findings, observations, or other activities in a clinical trial necessary for validation of the clinical data. Examples of these original documents and data records include, but are not limited to, hospital records, clinical and office charts, laboratory notes, memoranda, participants' memory aid or evaluation checklists, pharmacy dispensing records, recorded data from automated instruments, copies or transcriptions certified after verification as being accurate and complete, microfiches, photographic negatives, microfilm or magnetic media, x-rays, and participant files and records kept at the pharmacy, at the laboratories, and medico-technical departments involved in the clinical trial. All information on the Case Report Form (CRF) will be traceable to these source documents, which are generally maintained in the participant's study file. The source documents will include a copy of the signed Informed Consent/HIPAA authorization. The source document data collection forms for Screening, Inpatient

Pre-Challenge to Day of Challenge, Inpatient Post-Challenge, Flow Sheet of Stool and Emesis Record, Outpatient Visits and Adverse Events will also serve as Case Report Form (CRF) data collection instruments.

14 QUALITY ASSURANCE AND QUALITY CONTROL

All clinical trials conducted by Pharmaron are internally monitored for quality assurance and control. Quality Control (QC) and Quality Assurance (QA) procedures are designed to ensure that the site and investigator comply with applicable regulations, adhere to the IRB-approved protocol, generate quality data, protect data integrity, and safeguard the safety and well-being of study participants. The quality plan has the authority to enforce and correct deficiencies in clinical and laboratory conduct of a trial. The QC process involves day-to-day logic and edits checks of source documents, case report forms, and laboratory documents. The QA process involves periodic retrospective audits of study records and prospective reviews of clinical operations and assurance that all research required training has been completed as applicable. These audits also involve the review of the regulatory file, consent forms/process, eligibility, study product accountability and storage, specimen collection, processing, storage, and shipping. All observations are reviewed with the PI, coordinator, and Clinical Research Manager; Corrective Action Plans are formulated in response to any issue.

15 ETHICAL CONSIDERATIONS (AND INFORMED CONSENT)

15.1 Ethical Standard

The investigator will ensure that this study is conducted in full conformity with the principles set forth in The Belmont Report: Ethical Principles and Guidelines for the Protection of Human Subjects of Research of the US National Commission for the Protection of Human Subjects of Biomedical and Behavioral Research (April 18, 1979) and codified in 45 CFR Part 46 and/or the ICH E6; 62 Federal Regulations 25691 (1997).

15.2 Institutional Review Board

The local institution must provide for the review and approval of this protocol and the associated informed consent documents by an appropriate ethics review committee or Institutional Review Board (IRB). Any amendments to the protocol or consent materials must also be approved before they are placed into use unless it is in the best interest of the participants' safety to implement changes prior to approval. In both the United States and in other countries, only institutions holding a current U. S. Federal-Wide Assurance issued by OHRP may participate.

Refer to: http://www.hhs.gov/ohrp/assurances/.

Prior to enrollment of participants into this clinical study, the protocol and the informed consent form(s) will be reviewed and approved by the appropriate IRB. Any amendments to the protocol or consent materials will also be reviewed and approved by the appropriate IRB. The responsible official for the IRB will sign the IRB letter of approval of the protocol prior to the start of this clinical study. Should amendments to the protocol be required, the amendments will be submitted to the IRB; an IRB letter of approval of the amendment must be obtained prior to acting upon the amendment in the protocol.

15.3 Informed Consent Process

15.3.1 Consent and Other Informational Documents Provided to Participants

Consent forms describing in detail the study agent, study procedures, and risks are given to the participant and written documentation of informed consent is required prior to starting intervention/administering study product. The following consent materials are submitted with this protocol:

- Recruitment script
- Patient information sheet
- Informed consent form
- Study comprehension assessment test

15.3.2 Consent Procedures and Documentation

Informed consent is a process that is initiated prior to the individual's agreeing to participate in the study and continues throughout the individual's study participation. Extensive discussion of risks and possible benefits of this therapy will be provided to the participant. Consent forms describing in detail the study, study procedures and risks are given to the potential participant and written documentation of informed consent is required prior to starting any study procedure. Consent forms will be IRB-approved and the participant will be asked to read and review the document. Upon reviewing the document, the investigator will explain the research study to the participant and answer any questions that may arise. The participants will sign the informed consent document prior to any procedures being done specifically for the study. As a part of the consenting procedures the participants should pass a written examination (comprehension assessment test) with a score ≥70%, in order to demonstrate their comprehension of this study. If a participant scores at least 50%, then they will be given one more opportunity to re-test after further re-education. The participant should have the opportunity to discuss the study with their surrogates or think about it prior to agreeing to participate. The participant may withdraw consent at any time throughout the course of the trial. A copy of the informed consent document will be given to the participant for their records. The rights and welfare of the participant will be protected by emphasizing to them that the quality of their medical care will not be adversely affected if they decline to participate in this study.

15.4 Exclusion of Women, Minorities, and Children (Special Populations)

This clinical study will include women, adults 18 years of age and older, and all minorities who meet the inclusion/exclusion criteria, regardless of religion, sex, or ethnic background.

15.5 Participant and Data Confidentiality

Participant confidentiality is strictly held in trust by the investigators, their staff, and the Sponsor. This confidentiality is extended to cover testing of biological samples and other testing in addition to the clinical information relating to participant.

The study protocol, documentation, data, and all other information generated will be held in strict confidence. No information concerning the study or the data will be released to any unauthorized third party without prior written approval of the sponsor.

The study monitor or other authorized representatives of the sponsor may inspect all documents and records required to be maintained by the investigator, including but not limited to, medical

records (office, clinic, or hospital) and pharmacy records for the participants in this study. The clinical study site will permit access to such records.

Participant identity data will be contained in paper study records which will be kept in a locked file cabinet and in a secure electronic database, accessible only to authorized users at each clinical site. The study database will be user-restricted and password-protected. The study database will identify participants by a coded study participant ID number assigned by clinical site personnel, thus participants will not be identified by name.

15.5.1 Research Use of Stored Human Samples, Specimens, or Data

- Intended Use: data collected under this protocol may be used to study cholera and other enteric health conditions.
- Storage: Access to samples stored during the course of the study will be limited. Samples and data will be stored using codes assigned by the investigators. Data will be kept in password-protected computers. Only investigators will have access to the samples and data.
- Tracking: Data will be tracked using a central inventory system. .

Data collected for this study will be analyzed and stored at the Pharmaron study site. After the study is completed, the de-identified, archived data will be transmitted to and stored at PATH for use by other researchers including those outside of the study. Permission to transmit data to PATH will be included in the informed consent. When the study is completed, access to study data will be provided through PATH.

15.6 Future Use of Stored Samples

No stool, vomitus, or urine specimens will be stored after the end of the study for future use. All blood specimens collected during the study will be stored, analyzed, and destroyed within 10 years. The informed consent form includes language to allow participants to agree for potential future research with their blood samples and data related to enteric diseases. The samples and data used for future research will be de-identified and labeled with study identification number and not any personally identifiable information. A Biorepository Governance Plan will be maintained by PATH to describe the disposition and access to samples being stored for future research.

16 Data Handling and Recordkeeping

16.1 Data Collection and Data Management Responsibilities

The investigator is responsible for ensuring the accuracy, completeness, legibility, and timeliness of the data reported. Source documents are maintained for recording data for each participant enrolled in this clinical study. Study participants' data collected on the CRF during the trial will only be identified by participant number. If, as an exception, it is necessary for safety or regulatory reasons to identify the patient, both the Sponsor and the Investigator are bound to keep this information confidential.

All the information required by the protocol should be provided; any omissions require explanation. Each source document and corresponding CRF should be completed and available for monitoring and/or collection within a timely manner so that the monitor may check the entries for completeness, accuracy and legibility, ensure the CRF is signed by the Investigator, and transmit the data to the Sponsor.

All source documents and CRF should be completed in a neat, legible manner to ensure accurate interpretation of data. Black or blue ink is required to ensure clarity of reproduced copies. When making a change or correction, cross out the original entry with a single line and initial and date the change. DO NOT ERASE, OVERWRITE OR USE CORRECTION FLUID OR TAPE ON THE ORIGINAL.

PATH is responsible for data management activities and will develop a Data Management Plan (DMP) prior to participant enrollment. The DMP will describe roles of stakeholders and specific procedures to ensure appropriate handling of data at all steps of the data management process, to ensure a valid and high-quality database at the end of the study, ready for analysis.

The Investigator or designee must enter the information required by the protocol onto the CRF provided. The Sponsor's clinical site monitor will review the CRF for completeness and accuracy and instruct site personnel to make any required corrections or additions. Source data will be entered into a Medidata RAVE® data management system, managed by Pharmaron. Medidata RAVE® is a secure cloud-based application for building an electronic database that provides a robust audit trail. The electronic data capture (EDC) system includes password protection and internal quality checks, such as automatic range checks, to identify data that appear inconsistent, incomplete, or inaccurate.

Entered data will be verified by an independent monitoring CRO who will query the site for potential discrepancies following a review of the source data against the database for a percentage of the data. The site Investigator, or designee, will be responsible for the resolution of queries and appropriate documented changes in the database.

Automated queries generated by the EDC system are used to flag data discrepancies or inconsistencies. The site investigator, or designee, is required to provide a satisfactory resolution to the queries within the EDC system. In addition, the medical reviewers will also review data for medical inconsistencies, which will be raised as manual queries within the EDC system. The Data Manager reviews all the discrepancies to ensure that these are adequately resolved and to identify corrective and preventive actions if required. The responsible data manager will perform all activities as per standard operating procedures (SOPs) and/or work instructions. All study specific processes will be described in the DMP. Quality control audits of all key safety, laboratory, and clinical data in the database will be made after data entry has been completed. Coexistent medical conditions, adverse events and other medical events will be coded using MedDRA dictionary. Concomitant medications will be coded using WHO-DD dictionary. When the database has been declared to be complete and accurate, the database will be locked for final statistical analysis. Any changes to the database after that time can only be made by joint written agreement of the study team.

16.2 Study Records Retention

Study documents should be retained for a minimum of 2 years after the last approval of a marketing application in an ICH region and until there are no pending or contemplated marketing applications in an ICH region or until at least 2 years have elapsed since the formal discontinuation of clinical development of the investigational product. These documents should be retained for a longer period, however, if required by local regulations. No records will be destroyed without the written consent of the sponsor, if applicable. It is the responsibility of the sponsor to inform the investigator when these documents no longer need to be retained.

16.3 Protocol Deviations

A protocol deviation is any noncompliance with the clinical trial protocol, GCP, or MOP requirements. The noncompliance may be either on the part of the participant, the investigator, or the study site staff. As a result of deviations, corrective actions are to be developed by the site and implemented promptly.

These practices are consistent with ICH E6:

- 4.5 Compliance with Protocol, sections 4.5.1, 4.5.2, and 4.5.3
- 5.1 Quality Assurance and Quality Control, section 5.1.1
- 5.20 Noncompliance, sections 5.20.1, and 5.20.2.

It is the responsibility of the site to use continuous vigilance to identify and report deviations within 48 hours of identification of the protocol deviation to PATH. All deviations must be addressed in study source documents, reported PATH. Protocol deviations must be sent to Advarra per their guidelines. The site PI/study staff is responsible for knowing and adhering to Advarra requirements. PATH Medical Officer will be responsible for PATH Research Ethics Committee correspondence, expedited and other reporting requirements. Further details about the handling of protocol deviations will be included in the MOP.

16.4 Publications and Data Sharing Policy

The study sponsor will register this trial on ClinicalTrials.gov in accordance with Section 801 of the FDA Amendments Act of 2007.

It is understood by the investigators that the information generated in this study will be used by the sponsor in connection with the development of the product and therefore may be disclosed to government regulatory agencies in various countries. The sponsor (and manufacturer) also recognizes the importance of communicating study findings and therefore encourages their publication in reputable scientific journals and presentation at seminars or conferences, while protecting the integrity of the ongoing trial. Any publication, lecture, manuscripts of the findings of this study by any individual involved with the study will be governed by the procedure outlined in the Clinical Trial Agreement. Within any presentation or publication, confidentiality of individual participants will be maintained, with identification by participant code number and initials, if applicable.

17 Financing and Insurance

The trial is supported by a grant from [redacted].

PATH will provide locally admitted clinical trial insurance for the payment of any expenses related to the treatment of injuries directly attributable to participation in this study.

18 Conflict of Interest Policy

The independence of this study from any actual or perceived influence, such as by the pharmaceutical industry, is critical. Therefore, any actual conflict of interest of persons who have a role in the design, conduct, analysis, publication, or any aspect of this trial will be disclosed and managed. Furthermore, persons who have a perceived conflict of interest will be required to have such conflicts managed in a way that is appropriate to their participation in the trial. The study leadership has established policies and procedures for all study group members to disclose all

conflicts of interest and will establish a mechanism for the management of all reported dualities of interest.

19 References

1. de Hostos EL, Choy RK, Nguyen T. Developing novel antisecretory drugs to treat infectious diarrhea. Future Med Chem. 2011. 3(10): 1317-25.
2. Muanprasat C, Sonawane ND, Salinas D, Taddei A, Galietta LJ, Verkman AS. Discovery of glycine hydrazide pore-occluding CFTR inhibitors: mechanism, structure-activity analysis, and in vivo efficacy. J Gen Physiol. 2004. 124(2): 125-37.
3. Weiglmeier PR, Rösch P, Berkner H. Cure and curse: E. coli heat-stable enterotoxin and its receptor guanylyl cyclase C. Toxins (Basel). 2010 2(9):2213-29.
4. Schwertschlag U, Kumar A, Kochhar S, Ings R, Ji Y, de Hostos E L, Choy R, Salam M, Khan W A, Lin P Pharmacokinetics and Tolerability of iOWH032, an Inhibitor of the Cystic Fibrosis Transmembrane Conductance Regulator (CFTR) Chloride Channel, in Normal Volunteers and Cholera Patients. Gastroenterology 2014. 146 (5): Supplement 1, S-633.
5. Levin MM, Nalin DR, Craig JP, Hoover D, Bergquist EJ, Waterman D, Holley HP, Hornick RB, Pierce NP, Libonati JP. Immunity of cholera in man: relative role of antibacterial versus antitoxic immunity. Trans R Soc Trop Med Hyg. 1979;73(1):3-9.
6. Levin MM, Black RE, Clements ML, Cisneros L, Nalin DR, Young CR. Duration of infection-derived immunity to cholera. J Infect Dis. 1981 Jun;143(6):818-20.
7. Clements ML, Levin MM, Young CR, Black RE, Lim YL, Robins-Browne RM, Craig JP. Magnitude, kinetics, and duration of vibriocidal antibody responses in North Americans after ingestion of Vibrio cholerae. J Infect Dis. 1982 Apr;145(4):465-73
8. Black RE, Levin MM, Clements ML, Young CR, Svennerholm AM, Holmgren J. Protective efficacy in humans of killed whole-vibrio oral cholera vaccine with and without the B subunit of cholera toxin. Infect Immun. 1987 May;55(5):1116-20.
9. Levin MM, Kaper JB, Herrington D, Losonsky G, Morris JG, Clements ML, Black RE, Tall B, Hall R. Volunteer studies of deletion mutants of Vibrio cholerae O1 prepared by recombinant techniques. Infect Immun. 1988 Jan;56(1):161-7.
10. Levin MM, Kaper JB, Herrington D, Ketley J, Losonsky G, Tacket CO, Tall B, Cryz S. Safety, immunogenicity, and efficacy of recombinant live oral cholera vaccines, CVD 103 and CVD 103-HgR. Lancet. 1988 Aug 27;2(8609):467-70.
11. Tacket CO, Forrest B, Morona R, Attridge SR, LaBrooy J, Tall BD, Reymann M, Rowley D, Levin MM. Safety, immunogenicity, and efficacy against cholera challenge in humans of a typhoid-cholera hybrid vaccine derived from Salmonella typhi Ty21a. Infect Immun. 1990 Jun;58(6):1620-7.
12. Tacket CO, Losonsky G, Nataro JP, Cryz SJ, Edelman R, Kaper JB, Levin MM. Onset and duration of protective immunity in challenged volunteers after vaccination with live oral cholera vaccine CVD 103-HgR. J Infect Dis. 1992 Oct;166(4):837-41.
13. Losonsky GA, Lim Y, Motamedi P, Comstock LE, Johnson JA, Morris JG Jr, Tacket CO, Kaper JB, Levin MM. Vibriocidal antibody responses in North American volunteers exposed to wild-type or vaccine Vibrio cholerae O139 specificity and relevance to immunity. Clin Diagn Lab Immunol. 1997 May;4(3):264-9.
14. Tacket CO, Kotloff KL, Losonsky G, Nataro JP, Michalski J, Kaper JB, Edeman R, Levin MM. Volunteer studies investigating the safety and efficacy of live oral El Tor Vibrio cholerae O1 vaccine strain CVD 111. Am J Trop Med Hyg. 1997 May;56(5):533-7.
15. Sack DA, Tacket CO, Cohen MB, Sack RB, Losonsky GA, Shimko J, Nataro JP, Edelman R, Levine MM, Giannella RA, Schiff G, Lang D. Validation of a volunteer model of cholera with frozen bacteria as the challenge. Infect Immun. 1998 May;66(5):1968-72.

16. Chen WH, Cohen MB, Kirkpatrick BD, et al. Single-dose Live Oral Cholera Vaccine CVD 103-HgR Protects Against Human Experimental Infection With Vibrio cholerae O1 El Tor. Clin Infect Dis. 2016;62(11):1329-1335.
17. Rennels MB, Levine MM, Daya V, et al. Selective vs. nonselective media and direct plating vs. enrichment technique in isolation of Vibrio cholerae: recommendations for clinical laboratories. J Infect Dis. 1980 Sep;142(3):328-31.

APPENDIX A: PROTOCOL REVISIONS

Version	Date	Significant Revisions	Ethics/Regulatory Approvals
1.0	7/24/2019	N/A	
2.0	10/30/2019	Safety Monitoring, AE definition, cholera inoculum preparation procedures	Advarra IRB, FDA
3.0	12/23/2019	Exploratory endpoint, screening stool, Lab normal values, mITT description etc.	Advarra IRB, FDA
4.0	03/13/2020	Additional topline analyses	Advarra IRB, FDA

APPENDIX B: SCREENING TESTS

Analyte	Unit	Acceptable Values for Safety Labs:
White Blood Cell count (WBC)	thou/mcL	3600–11000 (2800–11,000 for African-Americans)
Absolute Neutrophil Count (ANC)	thou/mcL	1500–8000 (1200-8000 for African-Americans)
Hemoglobin (Females)	g/dL	F: 11.0–15.6
Hemoglobin (Males)	g/dL	M: 13.0–17.2
Platelet count	per mm^3	125,000–450,000
ALT	IU/L	<50
AST	IU/L	<40
Alkaline Phosphates	IU/L	44-147
Sodium	mEq/L	135-145
Potassium	mEq/L	3.5-5.5
Glucose (nonfasting)	mg/dL	≤ 115
Creatinine (females)	mg/dL	< 1.6
Creatinine (males)	mg/dL	< 1.6
Creatine phosphokinase (Males and females)	U/L	22-360
Albumin	g/dL	<3.3
Bilirubin, total	mg/dL	< 1.4 (unless known Gilbert's syndrome, then <2.0)
Glucose - urinalysis	n/a	trace or negative
Protein - urinalysis	n/a	≤ 1+
Occult blood - urinalysis	n/a	Small, trace or negative*
RBC (Females) - urinalysis	per hpf	0–4*
RBC (Males) - urinalysis	per hpf	0–4
Serum β-HCG	n/a	Negative
Hepatitis B surface antigen	n/a	Negative
Hepatitis C virus ELISA	n/a	Negative
HIV	n/a	Negative
n/a, not applicable<br>*presence is acceptable for menstruating women		
12-lead Electrocardiogram	Must not have any of the following, in order to be acceptable:<br>• Pathologic Q wave abnormalities<br>• Significant ST-T wave changes<br>• Left ventricular hypertrophy<br>• Right bundle branch block<br>• Left bundle branch block<br>• A-V heart block	

Analyte	Unit	Acceptable Values for Safety Labs:
		• Non-sinus rhythm, excluding isolated premature atrial contractions

APPENDIX C: SCHEDULE OF STUDY VISITS AND EVALUATIONS

Study visit	Visit 1 (screen)	*Inpatient Containment Period*											Visit 2	Visit 3	Visit 4
**Study Day**	$D_{-85\ to\ -7}$	D_{-1}	D_{1}	D_{2}	D_{3}	D_{4}	D_{5}	D_{6}	D_{7}	D_{8}	D_{9}	D_{10}	$D_{15\pm1}$	$D_{29\pm2}$	$D_{180\pm14}$
**Informed Consent**	√														
**Medical history, medications**	√														
**Comprehension Assessment Tool**	√														
**Physical examination**	√		√[h]	√[h]	√[h]	√[h]	√[h]	√[h]	√[h]	√[h]	√[h]	√[h]	√[h]	√[h]	
**Pregnancy test**[a]	√	√													
**Vital signs**	√		√[g]	√[g]	√[g]	√[g]	√[g]	√[g]	√[g]	√[g]	√[g]	√[g]			
**Screening laboratory**[b]	18 mL														
**12-lead ECG**	√														
**Pre-challenge Stool Sample**		√													
**Safety laboratory**[c]		10 mL											10 mL		
**Eligibility confirmation**	√	√	√												
**Challenge**			√												
**Blinded Study Drug Administration**[d]			(√)	(√)	(√)										
**3-day course of antibiotics**[e]							(√)	(√)	(√)						
**Observe & manage illness**[f]			√	√	√	√	√	√	√	(√)	(√)	(√)			
**Stool grading and cultures**[f]			√	√	√	√	√	√	√	(√)	(√)	(√)			
**pK analysis**[i]			√		√										
**Cumulative blood volume**	18 mL	10 mL	2 mL		2 mL								10 mL		
**Solicited AEs 7 days post-challenge**			√	√	√	√	√	√	√	√					
**Unsolicited AEs**			√	√	√	√	√	√	√	√	√	√	√	√	
**SAEs**			√	√	√	√	√	√	√	√	√	√	√	√	√
**Concomitant medications**			√	√	√	√	√	√	√	√	√	√	√	√	√
**Interval medical history**			√	√	√	√	√	√	√	√	√	√	√	√	√
**Unanticipated problems (UPs)**	√	√	√	√	√	√	√	√	√	√	√	√	√	√	√

a. For females of child bearing potential, a serum pregnancy test will be performed at screening and one day before challenge
b. Screening laboratory to include: Complete Blood Count (CBC) for WBC, ANC, Hemoglobin, and Platelets; Chemistry panel for Na, K, ALT, AST, Creatinine, Albumin, ALP, CPK, Glucose and Total Bilirubin; Serology for HbSAg, anti-HCV, and HIV; Blood Typing; and Urinalysis

c. Safety laboratory to include: Complete blood count (CBC) for WBC, ANC, Hemoglobin, and Platelets; and Chemistry panel for Na, K, ALT,AST,ALP, Creatinine, Albumin, and Total Bilirubin, CPK, Glucose
d. Blinded therapeutic dosing will start at the onset of symptoms or by 48 hours after ingesting the cholera challenge inoculum.
e. A 3-day course of antibiotics will be initiated upon satisfying the criteria for severe cholera diarrhea or approximately 4 day post-challenge, whichever is earlier
f. The observation and management of cholera illness (includes measurement of vomiting) and stool grading and stool cultures are intended to be performed throughout the post-challenge inpatient period, until discharge criteria are satisfied. For the duration of diarrhea, the participant will be requested to provide a urine specimen from every void that they experience; the specimen will be tested for urine specific gravity. In case of IV fluid replacement, serum electrolytes, BUN, and creatinine will be measured.
g. Vital signs will be measured at least 3 times daily (approximately every 8 ±1 hours). Vital signs will also be measured every 4 hours when a participant has a fever ≥ 39°C (102.1°F), until the participant has 2 consecutive temperatures of ≤ 38°C (100.4°F). On Day 1, including the pre-challenge vitals assessment, there will be 4 vital signs assessments (1x pre-challenge and 3x post-challenge).
h. Focused physical examination at the discretion of the physician
i. pK plasma samples for the levels of iOWH032 will be collected 7±1 hours after the first and last dosing in all participants

APPENDIX D: CLINICAL SAFETY LABORATORY TOXICITY SCALES

Test	Mild Grade 1	Moderate Grade 2	Severe Grade 3	Potentially Life Threatening Grade 4
WBC, increase	1.1 – 1.5 xULN	1.6 – 2.0 xULN	2.1 – 2.5 xULN	≥ 2.6 xULN
WBC, decrease*	1.1 – 1.5 xLLN	1.6 – 2.0 xLLN	2.1 – 2.5 xLLN	≥ 2.6 xLLN
ANC decrease	1.1 – 1.5 xLLN	1.6 – 2.0 xLLN	2.1 – 2.5 xLLN	≥ 2.6 xLLN
Hemoglobin decrease	1.1 – 1.5 xLLN	1.6 – 2.0 xLLN	2.0 – 3.0 xLLN	>3 xLLN
Platelet count	1.3 – 1.5 xLLN	1.6 – 2.0 xLLN	2.0 – 3.0 xLLN	>3 xLLN
Glucose Nonfasting, High	116-160	161-250	251-500	≥ 500
Glucose, Low	55-64	40-54	30-39	< 30
Sodium, low	132 -134	130 - 132	125 - 129	< 125
Sodium, high	146 – 148	149 - 150	151 - 152	> 152
Potassium, high	5.6 – 5.8	5.6 – 6.1	6.2 – 6.5	> 6.5
Potassium, low	3.3 – 3.4	3.1 – 3.2	2.9 – 3.0	<2.9
Creatinine*	1.1 – 1.5 xULN	1.6 – 2.0 xULN	2.1 – 3.0 xULN	> 3.0 xULN
AST (SGOT)	1.6 -.2.5 xULN	2.6 – 4.0 xULN	4.1 – 10.0 xULN	>10 xULN
ALT (SGPT)	1.6 -.2.5 xULN	2.6 – 4.0 xULN	4.1 – 10.0 xULN	>10 xULN
Alkaline Phosphatase	1.6 -.2.0 xULN	2.1 – 3.0 xULN	3.1 – 10.0 xULN	>10 xULN
Total Bilirubin	1.2 -.1.5 xULN	1.6 – 2.0 xULN	2.0 - 4.0 xULN	>4 xULN

ULN = upper limit of normal range

LLN = lower limit of normal range

*if the baseline is outside of the reference range (e.g., African-American acceptable value), then relative changes to the baseline value will be evaluated for toxicity

APPENDIX E: REACTOGENICITY AND SOLICITED ADVERSE EVENT TOXICITY SCALES

Grading of Stool Consistency

normal stool		*loose or diarrheal stool*		
Grade 1	Grade 2	Grade 3	Grade 4	Grade 5
Well formed; does not take the shape of the container	Soft; does not easily take the shape of the container	Thick liquid stool; easily takes the shape of the container	Opaque watery diarrheal stool	Clear watery or "rice water" diarrheal stool

Diarrhea Severity		
Mild	Moderate	Severe
2 or more loose stools of ≥ 200 mL within 48 hours *or* a single loose stool of ≥ 300 mL	Cumulative loose stools of ≥ 3 liter	Cumulative loose stools of ≥ 5 liters

Objective Reactogenicity	Mild Grade 1	*Moderate Grade 2*	*Severe Grade 3*	*Potentially Life Threatening Grade 4*
Diarrhea	*(refer to table above)*			*Requires hospitalization*
Vomiting (episodes/24 hour)	1-2	3-4	≥ 5	
Fever, °C (°F)	38.0 – 38.4 (100.4 – 101.1)	38.5 – 38.9 (101.2 – 102)	≥ 39 (≥ 102.1)	
Tachycardia, bpm	101 - 115	116 - 125	> 125	
Systolic Hypotension, mmHg	85 - 89	80 - 84	< 80	
Subjective Reactogenicity, includes: abdominal pain/cramping, malaise, nausea, anorexia, arthralgia	No interference with activity	Some interference with activity	Prevents daily activity	Requires hospitalization

APPENDIX F: EXAMPLES OF SUBSTRATES THAT ARE METABOLIZED BY CYP2C9

**Drug name**	**Class**
Irbesartan	Angiotensin II blocker
Losartan	Angiotensin II blocker
Phenytoin	Antiepileptic
Cyclophosphamide	Alkylating agent
Tamoxifen	Anti-estrogen
Fluvastatin	Statin
Celecoxib	NSAID
Diclofenac	NSAID
Ibuprofen	NSAID
Lornoxicam	NSAID
Meloxicam	NSAID
Naproxen	NSAID
Glibenclamide	Sulfonylurea
Glimepiride	Sulfonylurea
Glipizide	Sulfonylurea
Tolbutamide	Sulfonylurea
Warfarin	Anticoagulant
