# Supplementary material for: A Phase 2a randomized, single-center, double-blind, placebo-controlled study to evaluate the safety and preliminary efficacy of oral iOWH032 against cholera diarrhea in a controlled human infection model
Source: PLoS Negl Trop Dis. 2021 Nov 18;15(11):e0009969. doi: 10.1371/journal.pntd.0009969 (PMC8639072; doi:10.1371/journal.pntd.0009969)
Supplement: S2 Dataset — (RTF) [file pntd.0009969.s010.rtf]

PATH 
Protocol: DRG-032-PO-2-01-USA 	Page 1 of 36 	

Listing 16.2.6.1 Diarrhea Collection - Safety Population

Treatment
Group	Subject ID	Visit	None	Date and Time of
Collection (Study Day)	Volume of Diarrhea
Collection(ml)	Grade	Comments	
iOWH032	01003	Day 1	Yes					
		Day 2	No	05DEC2019/06:58 (1)	134.5	3		
				05DEC2019/08:29 (1)	289.31	3		
				05DEC2019/11:49 (1)	91.24	3		
				05DEC2019/12:50 (1)	50.13	3		
				05DEC2019/17:38 (1)	91.14	3		
				05DEC2019/20:53 (1)	167.25	3		
		Day 3	No	06DEC2019/01:03 (2)	125.82	3		
				06DEC2019/04:14 (2)	223.66	3		
				06DEC2019/07:33 (2)	198.37	3		
				06DEC2019/19:39 (2)	123.31	3		
		Day 4	No	07DEC2019/02:31 (3)	192.40	3		
				07DEC2019/07:52 (3)	202.20	4		
		Day 5	No	08DEC2019/06:45 (4)	206.80	3		
				08DEC2019/10:55 (4)	123.14	3		
				08DEC2019/12:44 (4)	151.87	3		
				08DEC2019/17:17 (4)	69.49	3		
		Day 6	Yes					
		Day 7	No	10DEC2019/22:12 (6)	257.91	3		
				10DEC2019/22:58 (6)	124.93	3		
 	
	01009	Day 1	Yes					
		Day 2	Yes					
 	
Study day is relative to the initiation of study drug on Day 1.
Program: l_diar.sas 	Executed: 01DEC2020 16:53   	


PATH 
Protocol: DRG-032-PO-2-01-USA 	Page 2 of 36 	

Listing 16.2.6.1 Diarrhea Collection - Safety Population

Treatment
Group	Subject ID	Visit	None	Date and Time of
Collection (Study Day)	Volume of Diarrhea
Collection(ml)	Grade	Comments	
iOWH032	01009	Day 3	Yes					
		Day 4	No	07DEC2019/10:53 (2)	163.18	3		
		Day 5	No	08DEC2019/17:12 (3)	111.17	3		
		Day 6	No	09DEC2019/08:13 (4)	100.49	4		
		Day 7	Yes					
 	
	01014	Day 1	Yes					
		Day 2	No	05DEC2019/21:51 (1)	66.91	4		
		Day 3	No	06DEC2019/09:09 (2)	89.56	4		
		Day 4	No	07DEC2019/01:00 (3)	62.62	4		
		Day 5	Yes					
		Day 6	No	09DEC2019/10:38 (5)	103.32	4		
				09DEC2019/22:53 (5)	73.67	4		
		Day 7	No	10DEC2019/11:35 (6)	63.35	3		
				10DEC2019/19:08 (6)	106.28	3		
 	
	01015	Day 1	Yes					
		Day 2	No	05DEC2019/07:18 (1)	111.2	3		
				05DEC2019/09:30 (1)	123.66	4		
		Day 3	No	06DEC2019/15:18 (2)	105.17	4		
		Day 4	No	07DEC2019/16:46 (3)	176.75	5		
		Day 5	Yes					
		Day 6	No	09DEC2019/11:49 (5)	23.62	3		
				09DEC2019/16:46 (5)	95.26	4		
 	
Study day is relative to the initiation of study drug on Day 1.
Program: l_diar.sas 	Executed: 01DEC2020 16:53   	


PATH 
Protocol: DRG-032-PO-2-01-USA 	Page 3 of 36 	

Listing 16.2.6.1 Diarrhea Collection - Safety Population

Treatment
Group	Subject ID	Visit	None	Date and Time of
Collection (Study Day)	Volume of Diarrhea
Collection(ml)	Grade	Comments	
iOWH032	01015	Day 7	No	10DEC2019/11:23 (6)	151.26	3		
				10DEC2019/19:44 (6)	106.68	3		
		Day 8 UNS 1	No	11DEC2019/11:07 (7)	109.43	3		
 	
	01018	Day 1	Yes					
		Day 2	No	05DEC2019/09:51 (1)	195.24	3		
				05DEC2019/10:22 (1)	122.61	3		
				05DEC2019/11:10 (1)	143.10	4		
				05DEC2019/12:09 (1)	325.85	4		
				05DEC2019/12:47 (1)	135.38	3		
				05DEC2019/14:33 (1)	175.81	4		
				05DEC2019/18:45 (1)	128.04	4		
				05DEC2019/21:08 (1)	185.43	4		
		Day 3	No	06DEC2019/07:06 (2)	483.87	4		
				06DEC2019/10:36 (2)	498.73	4		
				06DEC2019/11:13 (2)	187.65	4		
				06DEC2019/12:54 (2)	183.56	4		
				06DEC2019/14:09 (2)	196.68	4		
				06DEC2019/19:27 (2)	336.70	4		
				06DEC2019/21:29 (2)	334.11	4		
		Day 4	No	07DEC2019/00:26 (3)	133.96	4		
				07DEC2019/09:37 (3)	715.56	4		
				07DEC2019/10:42 (3)	219.96	4		
 	
Study day is relative to the initiation of study drug on Day 1.
Program: l_diar.sas 	Executed: 01DEC2020 16:53   	


PATH 
Protocol: DRG-032-PO-2-01-USA 	Page 4 of 36 	

Listing 16.2.6.1 Diarrhea Collection - Safety Population

Treatment
Group	Subject ID	Visit	None	Date and Time of
Collection (Study Day)	Volume of Diarrhea
Collection(ml)	Grade	Comments	
iOWH032	01018	Day 4	No	07DEC2019/21:45 (3)	194.86	3		
				07DEC2019/22:45 (3)	180.65	3		
		Day 5	No	08DEC2019/09:30 (4)	681.16	5		
				08DEC2019/11:15 (4)	155.02	5		
		Day 6	No	09DEC2019/11:41 (5)	128.37	3		
		Day 7	Yes					
 	
	01021	Day 1	Yes					
		Day 2	Yes					
		Day 3	Yes					
		Day 4	Yes					
		Day 5	Yes					
		Day 6	Yes					
		Day 7	Yes					
 	
	01028	Day 1	No	04DEC2019/18:34 (1)	6.36	3		
				04DEC2019/23:32 (1)	3.98	3		
		Day 2	Yes					
		Day 3	Yes					
		Day 4	Yes					
		Day 5	Yes					
		Day 6	No	09DEC2019/09:22 (6)	158.17	3		
		Day 7	Yes					
 	
	01029	Day 1	Yes					
 	
Study day is relative to the initiation of study drug on Day 1.
Program: l_diar.sas 	Executed: 01DEC2020 16:53   	


PATH 
Protocol: DRG-032-PO-2-01-USA 	Page 5 of 36 	

Listing 16.2.6.1 Diarrhea Collection - Safety Population

Treatment
Group	Subject ID	Visit	None	Date and Time of
Collection (Study Day)	Volume of Diarrhea
Collection(ml)	Grade	Comments	
iOWH032	01029	Day 2	No	05DEC2019/08:06 (1)	279.87	3		
				05DEC2019/12:16 (1)	234.83	4		
				05DEC2019/13:39 (1)	286.40	4		
				05DEC2019/16:52 (1)	119.62	4		
				05DEC2019/21:13 (1)	68.11	4		
				05DEC2019/23:44 (1)	122.40	4		
		Day 3	No	06DEC2019/04:45 (2)	289.27	4		
				06DEC2019/07:38 (2)	243.70	4		
				06DEC2019/09:03 (2)	131.71	4		
				06DEC2019/12:12 (2)	167.84	4		
				06DEC2019/14:25 (2)	142.87	4		
				06DEC2019/16:10 (2)	135.57	4		
				06DEC2019/17:56 (2)	274.19	4		
				06DEC2019/19:58 (2)	220.10	4		
				06DEC2019/21:38 (2)	119.12	4		
				06DEC2019/22:40 (2)	221.73	4		
				06DEC2019/23:57 (2)	171.29	4		
		Day 4	No	07DEC2019/00:33 (3)	95.68	5		
				07DEC2019/02:49 (3)	411.22	5		
				07DEC2019/07:49 (3)	562.88	5		
				07DEC2019/11:50 (3)	509.73	5		
				07DEC2019/13:21 (3)	346.92	5		
 	
Study day is relative to the initiation of study drug on Day 1.
Program: l_diar.sas 	Executed: 01DEC2020 16:53   	


PATH 
Protocol: DRG-032-PO-2-01-USA 	Page 6 of 36 	

Listing 16.2.6.1 Diarrhea Collection - Safety Population

Treatment
Group	Subject ID	Visit	None	Date and Time of
Collection (Study Day)	Volume of Diarrhea
Collection(ml)	Grade	Comments	
iOWH032	01029	Day 4	No	07DEC2019/14:50 (3)	382.12	5		
				07DEC2019/18:17 (3)	173.69	5		
		Day 5	No	08DEC2019/12:22 (4)	100.75	4		
				08DEC2019/16:19 (4)	213.75	4		
		Day 6	No	09DEC2019/08:47 (5)	372.22	4		
				09DEC2019/17:47 (5)	100.40	4		
		Day 7	No	10DEC2019/08:38 (6)	237.46	3		
				10DEC2019/22:35 (6)	96.80	3		
 	
	01043	Day 1	Yes					
		Day 2	No	29JAN2020/19:33 (-1)	162.59	3		
		Day 3	No	30JAN2020/09:09 (1)	396.95	4		
				30JAN2020/12:32 (1)	484.32	4		
				30JAN2020/14:06 (1)	406.05	5		
				30JAN2020/18:52 (1)	256.45	5		
				30JAN2020/22:14 (1)	158.66	5		
		Day 4	No	31JAN2020/06:31 (2)	271.48	4		
				31JAN2020/12:53 (2)	291.20	5		
				31JAN2020/16:22 (2)	189.82	5		
		Day 5	No	01FEB2020/11:49 (3)	289.04	5		
		Day 6	No	02FEB2020/09:55 (4)	170.32	4		
				02FEB2020/23:10 (4)	102.40	3		
		Day 7	Yes					
 	
Study day is relative to the initiation of study drug on Day 1.
Program: l_diar.sas 	Executed: 01DEC2020 16:53   	


PATH 
Protocol: DRG-032-PO-2-01-USA 	Page 7 of 36 	

Listing 16.2.6.1 Diarrhea Collection - Safety Population

Treatment
Group	Subject ID	Visit	None	Date and Time of
Collection (Study Day)	Volume of Diarrhea
Collection(ml)	Grade	Comments	
iOWH032	01045	Day 1	Yes					
		Day 2	Yes					
		Day 3	Yes					
		Day 4	Yes					
		Day 5	Yes					
		Day 6	Yes					
		Day 7	Yes					
 	
	01048	Day 1	Yes					
		Day 2	No	29JAN2020/04:40 (1)	767.11	3		
				29JAN2020/11:02 (1)	91.60	4		
				29JAN2020/19:40 (1)	69.70	4		
		Day 3	No	30JAN2020/05:35 (2)	147.45	4		
				30JAN2020/05:55 (2)	86.69	4		
				30JAN2020/09:12 (2)	108.94	5		
				30JAN2020/12:43 (2)	98.08	5		
		Day 4	No	31JAN2020/06:14 (3)	177.80	3		
				31JAN2020/06:58 (3)	122.85	4		
				31JAN2020/10:48 (3)	145.72	5		
				31JAN2020/20:28 (3)	166.30	4		
		Day 5	No	01FEB2020/09:29 (4)	193.53	5		
				01FEB2020/11:42 (4)	130.93	5		
				01FEB2020/12:45 (4)	151.16	5		
 	
Study day is relative to the initiation of study drug on Day 1.
Program: l_diar.sas 	Executed: 01DEC2020 16:53   	


PATH 
Protocol: DRG-032-PO-2-01-USA 	Page 8 of 36 	

Listing 16.2.6.1 Diarrhea Collection - Safety Population

Treatment
Group	Subject ID	Visit	None	Date and Time of
Collection (Study Day)	Volume of Diarrhea
Collection(ml)	Grade	Comments	
iOWH032	01048	Day 5	No	01FEB2020/14:52 (4)	74.82	5		
		Day 6	Yes					
		Day 7	Yes					
 	
	01050	Day 1	Yes					
		Day 2	Yes					
		Day 3	Yes					
		Day 4	Yes					
		Day 5	No	01FEB2020/07:05 (3)	324.22	3		
		Day 6	Yes					
		Day 7	Yes					
 	
	01053	Day 1	Yes					
		Day 2	No	29JAN2020/07:18 (1)	483.99	3		
				29JAN2020/09:26 (1)	268.85	3		
				29JAN2020/12:27 (1)	157.92	4		
		Day 3	No	30JAN2020/01:44 (2)	451.13	4		
				30JAN2020/04:35 (2)	355.31	4		
				30JAN2020/08:28 (2)	625.03	5		
				30JAN2020/11:00 (2)	352.05	5		
				30JAN2020/13:59 (2)	401.22	5		
				30JAN2020/15:49 (2)	304.20	5		
				30JAN2020/17:15 (2)	340.21	5		
				30JAN2020/20:50 (2)	411.99	5		
 	
Study day is relative to the initiation of study drug on Day 1.
Program: l_diar.sas 	Executed: 01DEC2020 16:53   	


PATH 
Protocol: DRG-032-PO-2-01-USA 	Page 9 of 36 	

Listing 16.2.6.1 Diarrhea Collection - Safety Population

Treatment
Group	Subject ID	Visit	None	Date and Time of
Collection (Study Day)	Volume of Diarrhea
Collection(ml)	Grade	Comments	
iOWH032	01053	Day 3	No	30JAN2020/22:18 (2)	316.39	5		
		Day 4	No	31JAN2020/11:26 (3)	214.11	5		
				31JAN2020/13:09 (3)	279.02	5		
				31JAN2020/15:43 (3)	237.34	5		
				31JAN2020/16:50 (3)	254.30	5		
		Day 5	No	01FEB2020/00:21 (4)	206.06	5		
				01FEB2020/17:48 (4)	263.10	4		
		Day 6	No	02FEB2020/04:35 (5)	230.70	3		
				02FEB2020/10:45 (5)	127.40	3		
				02FEB2020/15:38 (5)	89.03	3		
		Day 7	No	03FEB2020/01:47 (6)	202.79	3		
				03FEB2020/04:56 (6)	158.75	3		
 	
	01055	Day 1	Yes					
		Day 2	No	05DEC2019/09:47 (1)	102.17	3		
				05DEC2019/12:12 (1)	137.37	3		
				05DEC2019/13:45 (1)	126.66	3		
				05DEC2019/14:29 (1)	125.62	4		
				05DEC2019/15:07 (1)	138.85	4		
				05DEC2019/16:24 (1)	156.79	4		
				05DEC2019/17:36 (1)	75.23	4		
				05DEC2019/19:40 (1)	81.42	3		
				05DEC2019/20:58 (1)	122.80	4		
 	
Study day is relative to the initiation of study drug on Day 1.
Program: l_diar.sas 	Executed: 01DEC2020 16:53   	


PATH 
Protocol: DRG-032-PO-2-01-USA 	Page 10 of 36 	

Listing 16.2.6.1 Diarrhea Collection - Safety Population

Treatment
Group	Subject ID	Visit	None	Date and Time of
Collection (Study Day)	Volume of Diarrhea
Collection(ml)	Grade	Comments	
iOWH032	01055	Day 3	No	06DEC2019/05:41 (2)	146.54	4		
				06DEC2019/09:34 (2)	78.90	4		
		Day 4	No	07DEC2019/04:40 (3)	150.75	3		
				07DEC2019/13:58 (3)	70.98	3		
		Day 5	No	08DEC2019/04:59 (4)	115.92	3		
				08DEC2019/09:48 (4)	126.78	4		
		Day 6	Yes					
		Day 7	Yes					
 	
	01058	Day 1	Yes					
		Day 2	No	05DEC2019/12:19 (1)	340.68	3		
				05DEC2019/15:20 (1)	170.51	3		
				05DEC2019/17:18 (1)	151.55	4		
				05DEC2019/20:50 (1)	316.72	5		
		Day 3	No	06DEC2019/00:44 (2)	216.68	4		
				06DEC2019/02:16 (2)	151.89	4		
				06DEC2019/14:57 (2)	442.61	4		
				06DEC2019/22:15 (2)	182.34	4		
		Day 4	No	07DEC2019/11:38 (3)	341.13	4		
				07DEC2019/18:52 (3)	140.78	3		
		Day 5	No	08DEC2019/08:22 (4)	258.26	3		
				08DEC2019/18:00 (4)	168.04	3		
		Day 6	Yes					
 	
Study day is relative to the initiation of study drug on Day 1.
Program: l_diar.sas 	Executed: 01DEC2020 16:53   	


PATH 
Protocol: DRG-032-PO-2-01-USA 	Page 11 of 36 	

Listing 16.2.6.1 Diarrhea Collection - Safety Population

Treatment
Group	Subject ID	Visit	None	Date and Time of
Collection (Study Day)	Volume of Diarrhea
Collection(ml)	Grade	Comments	
iOWH032	01058	Day 7	No	10DEC2019/10:20 (6)	131.29	3		
 	
	01062	Day 1	Yes					
		Day 2	Yes					
		Day 3	No	30JAN2020/13:28 (1)	471.47	3		
				30JAN2020/18:05 (1)	263.60	4		
				30JAN2020/20:08 (1)	294.41	4		
				30JAN2020/23:58 (1)	109.41	4		
		Day 4	Yes					
		Day 5	No	01FEB2020/09:35 (3)	267.87	3		
		Day 6	Yes					
		Day 7	Yes					
 	
	01064	Day 1	Yes					
		Day 2	Yes					
		Day 3	Yes					
		Day 4	No	31JAN2020/19:12 (2)	95.89	3		
		Day 5	Yes					
		Day 6	Yes					
		Day 7	Yes					
		Visit 2 UNS 1	No	12FEB2020/10:59 (14)	85.28			
 	
	01070	Day 1	Yes					
		Day 2	No	29JAN2020/07:34 (1)	258.32	3		
				29JAN2020/16:25 (1)	103.90	4		
 	
Study day is relative to the initiation of study drug on Day 1.
Program: l_diar.sas 	Executed: 01DEC2020 16:53   	


PATH 
Protocol: DRG-032-PO-2-01-USA 	Page 12 of 36 	

Listing 16.2.6.1 Diarrhea Collection - Safety Population

Treatment
Group	Subject ID	Visit	None	Date and Time of
Collection (Study Day)	Volume of Diarrhea
Collection(ml)	Grade	Comments	
iOWH032	01070	Day 2	No	29JAN2020/18:22 (1)	76.86	4		
				29JAN2020/22:02 (1)	82.02	4		
		Day 3	No	30JAN2020/03:52 (2)	490.03	4		
				30JAN2020/06:26 (2)	407.02	4		
				30JAN2020/11:37 (2)	542.26	5		
				30JAN2020/13:32 (2)	383.85	5		
				30JAN2020/16:50 (2)	673.09	5		
				30JAN2020/18:39 (2)	249.67	5		
				30JAN2020/21:53 (2)	246.26	5		
		Day 4	No	31JAN2020/04:28 (3)	239.06	5		
				31JAN2020/11:14 (3)	589.04	5		
		Day 5	No	01FEB2020/00:13 (4)	341.83	5		
				01FEB2020/12:38 (4)	502.96	5		
				01FEB2020/14:23 (4)	476.55	5		
				01FEB2020/17:52 (4)	348.77	5		
		Day 6	No	02FEB2020/16:46 (5)	89.84	4		
		Day 7	No	03FEB2020/10:41 (6)	316.75	4		
				03FEB2020/14:08 (6)	129.13	3		
 	
	01071	Day 1	Yes					
		Day 2	No	05DEC2019/14:48 (1)	67.03	4		
				05DEC2019/19:01 (1)	143.00	5		
		Day 3	No	06DEC2019/11:10 (2)	312.07	4		
 	
Study day is relative to the initiation of study drug on Day 1.
Program: l_diar.sas 	Executed: 01DEC2020 16:53   	


PATH 
Protocol: DRG-032-PO-2-01-USA 	Page 13 of 36 	

Listing 16.2.6.1 Diarrhea Collection - Safety Population

Treatment
Group	Subject ID	Visit	None	Date and Time of
Collection (Study Day)	Volume of Diarrhea
Collection(ml)	Grade	Comments	
iOWH032	01071	Day 4	No	07DEC2019/08:12 (3)	125.90	4		
		Day 5	No	08DEC2019/08:57 (4)	164.50	4		
		Day 6	No	09DEC2019/00:12 (5)	134.77	3		
		Day 7	Yes					
 	
	01077	Day 1	Yes					
		Day 2	No	29JAN2020/07:58 (1)	234.37	3		
		Day 3	Yes					
		Day 4	No	31JAN2020/08:09 (3)	164.92	4		
		Day 5	Yes					
		Day 6	Yes					
		Day 7	Yes					
 	
	01087	Day 1	Yes					
		Day 2	No	29JAN2020/07:13 (1)	139.01	4		
				29JAN2020/08:53 (1)	101.35	4		
				29JAN2020/20:40 (1)	84.30	3		
		Day 3	No	30JAN2020/14:33 (2)	116.40	4		
		Day 4	No	31JAN2020/14:47 (3)	88.51	3		
				31JAN2020/20:12 (3)	109.13	4		
		Day 5	No	01FEB2020/09:16 (4)	176.18	5		
		Day 6	Yes					
		Day 7	No	03FEB2020/17:43 (6)	186.29	3		
 	
	01099	Day 1	Yes					
 	
Study day is relative to the initiation of study drug on Day 1.
Program: l_diar.sas 	Executed: 01DEC2020 16:53   	


PATH 
Protocol: DRG-032-PO-2-01-USA 	Page 14 of 36 	

Listing 16.2.6.1 Diarrhea Collection - Safety Population

Treatment
Group	Subject ID	Visit	None	Date and Time of
Collection (Study Day)	Volume of Diarrhea
Collection(ml)	Grade	Comments	
iOWH032	01099	Day 2	No	29JAN2020/16:39 (1)	116.54	3		
				29JAN2020/19:12 (1)	248.44	3		
				29JAN2020/21:15 (1)	276.55	4		
				29JAN2020/22:58 (1)	228.95	4		
		Day 3	No	30JAN2020/02:00 (2)	288.80	4		
				30JAN2020/07:40 (2)	301.66	4		
				30JAN2020/09:27 (2)	294.84	5		
				30JAN2020/10:49 (2)	122.87	5		
				30JAN2020/13:52 (2)	451.23	5		
				30JAN2020/15:52 (2)	351.81	5		
				30JAN2020/18:13 (2)	181.75	4		
				30JAN2020/19:59 (2)	180.11	4		
				30JAN2020/22:53 (2)	280.44	4		
		Day 4	No	31JAN2020/02:59 (3)	170.62	3		
				31JAN2020/09:10 (3)	293.55	4		
				31JAN2020/16:30 (3)	154.12	3		
				31JAN2020/19:43 (3)	227.44	4		
		Day 5	No	01FEB2020/04:32 (4)	513.59	5		
				01FEB2020/08:58 (4)	262.49	4		
				01FEB2020/10:02 (4)	284.04	5		
				01FEB2020/11:30 (4)	282.34	5		
				01FEB2020/13:16 (4)	170.90	4		
 	
Study day is relative to the initiation of study drug on Day 1.
Program: l_diar.sas 	Executed: 01DEC2020 16:53   	


PATH 
Protocol: DRG-032-PO-2-01-USA 	Page 15 of 36 	

Listing 16.2.6.1 Diarrhea Collection - Safety Population

Treatment
Group	Subject ID	Visit	None	Date and Time of
Collection (Study Day)	Volume of Diarrhea
Collection(ml)	Grade	Comments	
iOWH032	01099	Day 5	No	01FEB2020/15:30 (4)	211.49	5		
				01FEB2020/18:52 (4)	236.28	4		
				01FEB2020/20:44 (4)	198.31	4		
				01FEB2020/22:40 (4)	236.87	4		
		Day 6	No	02FEB2020/00:33 (5)	281.83	4		
				02FEB2020/06:40 (5)	383.26	4		
				02FEB2020/09:12 (5)	220.54	4		
				02FEB2020/10:17 (5)	90.24	4		
		Day 7	Yes					
 	
	01104	Day 1	Yes					
		Day 2	Yes					
		Day 3	Yes					
		Day 4	Yes					
		Day 5	Yes					
		Day 6	Yes					
		Day 7	Yes					
 	
Placebo	01004	Day 1	Yes					
		Day 2	Yes					
		Day 3	No	06DEC2019/05:02 (1)	112.03	4		
				06DEC2019/09:26 (1)	242.31	4		
				06DEC2019/12:24 (1)	162.22	4		
				06DEC2019/17:22 (1)	74.58	3		
 	
Study day is relative to the initiation of study drug on Day 1.
Program: l_diar.sas 	Executed: 01DEC2020 16:53   	


PATH 
Protocol: DRG-032-PO-2-01-USA 	Page 16 of 36 	

Listing 16.2.6.1 Diarrhea Collection - Safety Population

Treatment
Group	Subject ID	Visit	None	Date and Time of
Collection (Study Day)	Volume of Diarrhea
Collection(ml)	Grade	Comments	
Placebo	01004	Day 3	No	06DEC2019/20:37 (1)	214.23	3		
				06DEC2019/23:27 (1)	121.69	3		
		Day 4	No	07DEC2019/01:14 (2)	157.43	4		
				07DEC2019/06:50 (2)	148.26	4		
				07DEC2019/11:34 (2)	154.87	4		
				07DEC2019/13:39 (2)	63.07	4		
				07DEC2019/23:34 (2)	114.36	3		
		Day 5	No	08DEC2019/07:47 (3)	97.60	4		
				08DEC2019/14:22 (3)	104.85	4		
				08DEC2019/18:22 (3)	176.06	3		
				08DEC2019/20:39 (3)	91.37	3		
		Day 6	No	09DEC2019/10:42 (4)	134.32	3		
				09DEC2019/13:18 (4)	76.32	4		
				09DEC2019/16:14 (4)	149.10	3		
		Day 7	No	10DEC2019/08:50 (5)	114.98	3		
 	
	01007	Day 1	Yes					
		Day 2	No	05DEC2019/20:03 (1)	245.21	4		
				05DEC2019/23:08 (1)	217.27	4		
		Day 3	No	06DEC2019/16:51 (2)	265.05	4		
				06DEC2019/19:32 (2)	254.00	4		
				06DEC2019/21:34 (2)	186.88	4		
		Day 4	No	07DEC2019/03:39 (3)	206.86	5		
 	
Study day is relative to the initiation of study drug on Day 1.
Program: l_diar.sas 	Executed: 01DEC2020 16:53   	


PATH 
Protocol: DRG-032-PO-2-01-USA 	Page 17 of 36 	

Listing 16.2.6.1 Diarrhea Collection - Safety Population

Treatment
Group	Subject ID	Visit	None	Date and Time of
Collection (Study Day)	Volume of Diarrhea
Collection(ml)	Grade	Comments	
Placebo	01007	Day 4	No	07DEC2019/10:17 (3)	501.83	4		
				07DEC2019/17:58 (3)	94.25			
				07DEC2019/21:50 (3)	207.12	3		
		Day 5	No	08DEC2019/07:41 (4)	411.43	5		
				08DEC2019/17:43 (4)	260.18	4		
		Day 6	No	09DEC2019/10:57 (5)	342.04	4		
				09DEC2019/22:10 (5)	372.51	4		
		Day 7	Yes					
 	
	01017	Day 1	Yes					
		Day 2	No	05DEC2019/07:12 (1)	297.9	3		
				05DEC2019/07:23 (1)	118.3	4		
				05DEC2019/07:59 (1)	141.43	4		
				05DEC2019/11:27 (1)	98.82	4		
				05DEC2019/18:00 (1)	121.57	4		
		Day 3	No	06DEC2019/00:57 (2)	181.07	4		
				06DEC2019/05:55 (2)	114.13	5		
				06DEC2019/08:36 (2)	116.54	5		
		Day 4	No	07DEC2019/02:35 (3)	88.06	5		
				07DEC2019/03:01 (3)	200	5		
		Day 5	No	08DEC2019/05:27 (4)	120.17	3		
				08DEC2019/23:17 (4)	140.39	4		
		Day 6	No	09DEC2019/10:46 (5)	71.97	4		
 	
Study day is relative to the initiation of study drug on Day 1.
Program: l_diar.sas 	Executed: 01DEC2020 16:53   	


PATH 
Protocol: DRG-032-PO-2-01-USA 	Page 18 of 36 	

Listing 16.2.6.1 Diarrhea Collection - Safety Population

Treatment
Group	Subject ID	Visit	None	Date and Time of
Collection (Study Day)	Volume of Diarrhea
Collection(ml)	Grade	Comments	
Placebo	01017	Day 6	No	09DEC2019/17:16 (5)	51.47	4		
		Day 7	Yes					
		Day 8 UNS 1	No	11DEC2019/11:02 (7)	169.05	3		
				11DEC2019/19:56 (7)	239.97	3		
				11DEC2019/20:30 (7)	145.76	3		
		Day 9 UNS 1	No	12DEC2019/06:53 (8)	175.53	4		
 	
	01030	Day 1	Yes					
		Day 2	No	05DEC2019/13:08 (1)	406.86	4		
				05DEC2019/14:24 (1)	202.83	4		
				05DEC2019/15:26 (1)	209.56	4		
				05DEC2019/16:32 (1)	161.19	4		
				05DEC2019/17:42 (1)	130.22	5		
				05DEC2019/18:19 (1)	84.41	5		
				05DEC2019/19:18 (1)	194.67	5		
				05DEC2019/20:11 (1)	101.80	5		
				05DEC2019/22:01 (1)	192.18	5		
				05DEC2019/22:57 (1)	218.95	5		
				05DEC2019/23:39 (1)	132.61	4		
		Day 3	No	06DEC2019/03:09 (2)	241.37	4		
				06DEC2019/04:00 (2)	158.92	4		
				06DEC2019/07:15 (2)	277.89	4		
				06DEC2019/08:59 (2)	143.43	4		
 	
Study day is relative to the initiation of study drug on Day 1.
Program: l_diar.sas 	Executed: 01DEC2020 16:53   	


PATH 
Protocol: DRG-032-PO-2-01-USA 	Page 19 of 36 	

Listing 16.2.6.1 Diarrhea Collection - Safety Population

Treatment
Group	Subject ID	Visit	None	Date and Time of
Collection (Study Day)	Volume of Diarrhea
Collection(ml)	Grade	Comments	
Placebo	01030	Day 3	No	06DEC2019/10:15 (2)	404.52	4		
				06DEC2019/15:07 (2)	328.31	5		
				06DEC2019/17:19 (2)	54.71	4		
		Day 4	No	07DEC2019/07:17 (3)	281.25	4		
				07DEC2019/22:00 (3)	138.25	3		
		Day 5	No	08DEC2019/01:26 (4)	165.84	3		
		Day 6	No	09DEC2019/19:48 (5)	145.08	3		
		Day 7	Yes					
 	
	01031	Day 1	Yes					
		Day 2	No	05DEC2019/19:30 (1)	93.67	3		
		Day 3	No	06DEC2019/07:42 (2)	114.79	3		
				06DEC2019/09:49 (2)	113.57	3		
				06DEC2019/13:59 (2)	121.95	4		
				06DEC2019/23:10 (2)	49.71	4		
		Day 4	No	07DEC2019/11:57 (3)	118.61			
		Day 5	No	08DEC2019/11:19 (4)	184.23	5		
		Day 6	No	09DEC2019/10:52 (5)	156.59	4		
				09DEC2019/20:40 (5)	128.98	3		
		Day 7	No	10DEC2019/19:29 (6)	123.56	3		
		Day 8 UNS 1	No	11DEC2019/11:19 (7)	152.71	3		
 	
	01038	Day 1	Yes					
		Day 2	No	05DEC2019/12:35 (1)	110.94	3		
 	
Study day is relative to the initiation of study drug on Day 1.
Program: l_diar.sas 	Executed: 01DEC2020 16:53   	


PATH 
Protocol: DRG-032-PO-2-01-USA 	Page 20 of 36 	

Listing 16.2.6.1 Diarrhea Collection - Safety Population

Treatment
Group	Subject ID	Visit	None	Date and Time of
Collection (Study Day)	Volume of Diarrhea
Collection(ml)	Grade	Comments	
Placebo	01038	Day 2	No	05DEC2019/13:32 (1)	143.90	3		
				05DEC2019/14:08 (1)	101.84	4		
				05DEC2019/14:52 (1)	127.12	4		
		Day 3	No	06DEC2019/00:38 (2)	45.52	4		
				06DEC2019/07:58 (2)	137.49	4		
				06DEC2019/08:27 (2)	73.78	4		
				06DEC2019/09:44 (2)	99.61	4		
				06DEC2019/10:59 (2)	84.23	4		
				06DEC2019/11:38 (2)	65.26	4		
				06DEC2019/12:21 (2)	84.62	4		
				06DEC2019/13:03 (2)	121.66	4		
				06DEC2019/13:48 (2)	54.24	4		
				06DEC2019/14:38 (2)	89.91	4		
				06DEC2019/15:32 (2)	95.78	4		
				06DEC2019/16:22 (2)	56.42	4		
				06DEC2019/16:57 (2)	98.01	4		
				06DEC2019/17:50 (2)	109.07	4		
				06DEC2019/18:30 (2)	45.52	4		
				06DEC2019/19:54 (2)	105.22	4		
				06DEC2019/21:25 (2)	61.83	4		
				06DEC2019/22:37 (2)	142.50	4		
				06DEC2019/23:32 (2)	94.71	4		
 	
Study day is relative to the initiation of study drug on Day 1.
Program: l_diar.sas 	Executed: 01DEC2020 16:53   	


PATH 
Protocol: DRG-032-PO-2-01-USA 	Page 21 of 36 	

Listing 16.2.6.1 Diarrhea Collection - Safety Population

Treatment
Group	Subject ID	Visit	None	Date and Time of
Collection (Study Day)	Volume of Diarrhea
Collection(ml)	Grade	Comments	
Placebo	01038	Day 4	No	07DEC2019/02:26 (3)	100.98	4		
				07DEC2019/05:12 (3)	138.81	4		
				07DEC2019/06:56 (3)	140.95	4		
				07DEC2019/07:33 (3)	175.31	4		
				07DEC2019/08:10 (3)	137.47	4		
				07DEC2019/08:37 (3)	109.90	4		
				07DEC2019/09:16 (3)	92.99	4		
				07DEC2019/11:17 (3)	102.56	4		
				07DEC2019/12:31 (3)	79.34	4		
				07DEC2019/14:44 (3)	45.19	4		
		Day 5	Yes					
		Day 6	Yes					
		Day 7	Yes					
 	
	01040	Day 1	Yes					
		Day 2	No	29JAN2020/18:58 (1)	409.87	3		
		Day 3	No	30JAN2020/13:56 (2)	127.73	3		
		Day 4	No	31JAN2020/17:33 (3)	129.28	3		
		Day 5	No	01FEB2020/04:04 (4)	238.79	4		
		Day 6	Yes					
		Day 7	Yes					
 	
	01041	Day 1	Yes					
		Day 2	No	29JAN2020/10:04 (1)	147.66	3		
 	
Study day is relative to the initiation of study drug on Day 1.
Program: l_diar.sas 	Executed: 01DEC2020 16:53   	


PATH 
Protocol: DRG-032-PO-2-01-USA 	Page 22 of 36 	

Listing 16.2.6.1 Diarrhea Collection - Safety Population

Treatment
Group	Subject ID	Visit	None	Date and Time of
Collection (Study Day)	Volume of Diarrhea
Collection(ml)	Grade	Comments	
Placebo	01041	Day 2	No	29JAN2020/11:38 (1)	167.85	3		
				29JAN2020/20:14 (1)	243.72	3		
		Day 3	No	30JAN2020/06:55 (2)	192.14	4		
				30JAN2020/12:45 (2)	154.97	4		
				30JAN2020/21:56 (2)	56.27	3		
		Day 4	No	31JAN2020/03:43 (3)	49.84	3		
				31JAN2020/09:15 (3)	72.07	3		
				31JAN2020/13:21 (3)	111.95	4		
				31JAN2020/18:28 (3)	69.65	4		
		Day 5	No	01FEB2020/12:33 (4)	160.18	5		
				01FEB2020/21:15 (4)	160.16	4		
		Day 6	Yes					
		Day 7	Yes					
 	
	01042	Day 1	Yes					
		Day 2	Yes					
		Day 3	No	06DEC2019/22:56 (1)	176.24	3		
		Day 4	No	07DEC2019/20:15 (2)	306.75	3		
		Day 5	Yes					
		Day 6	Yes					
		Day 7	Yes					
 	
	01046	Day 1	Yes					
		Day 2	No	05DEC2019/05:50 (1)	211.39	3		
 	
Study day is relative to the initiation of study drug on Day 1.
Program: l_diar.sas 	Executed: 01DEC2020 16:53   	


PATH 
Protocol: DRG-032-PO-2-01-USA 	Page 23 of 36 	

Listing 16.2.6.1 Diarrhea Collection - Safety Population

Treatment
Group	Subject ID	Visit	None	Date and Time of
Collection (Study Day)	Volume of Diarrhea
Collection(ml)	Grade	Comments	
Placebo	01046	Day 2	No	05DEC2019/07:36 (1)	26.87	3		
				05DEC2019/14:50 (1)	184.59	4		
				05DEC2019/15:12 (1)	132.32	4		
				05DEC2019/15:23 (1)	98.33	4		
				05DEC2019/16:10 (1)	ND	4		
				05DEC2019/16:49 (1)	203.22	5		
				05DEC2019/17:48 (1)	99.56	4		
				05DEC2019/18:28 (1)	233.70	5		
				05DEC2019/21:05 (1)	484.53	5		
				05DEC2019/23:13 (1)	432.55	5		
		Day 3	No	06DEC2019/06:02 (2)	237.30	4		
				06DEC2019/06:25 (2)	178.89	4		
				06DEC2019/07:46 (2)	166.44	3		
				06DEC2019/16:30 (2)	169.74	4		
		Day 4	No	07DEC2019/06:24 (3)	259.70	4		
				07DEC2019/07:10 (3)	217.14	4		
		Day 5	No	08DEC2019/05:49 (4)	98.30	4		
				08DEC2019/06:00 (4)	114.62	4		
				08DEC2019/07:20 (4)	350.87	4		
		Day 6	No	09DEC2019/06:30 (5)	390.59	3		
				09DEC2019/18:53 (5)	170.64	3		
		Day 7	Yes					
 	
Study day is relative to the initiation of study drug on Day 1.
Program: l_diar.sas 	Executed: 01DEC2020 16:53   	


PATH 
Protocol: DRG-032-PO-2-01-USA 	Page 24 of 36 	

Listing 16.2.6.1 Diarrhea Collection - Safety Population

Treatment
Group	Subject ID	Visit	None	Date and Time of
Collection (Study Day)	Volume of Diarrhea
Collection(ml)	Grade	Comments	
Placebo	01046	Day 8 UNS 1	No	11DEC2019/20:29 (7)	245.49	3		
		Day 9 UNS 1	No	12DEC2019/05:30 (8)	497.35	3		
				12DEC2019/08:29 (8)	204.45	3		
		Day 10 UNS 1	No	13DEC2019/06:19 (9)	449.37	3		
		Visit 2 UNS 1	No	17DEC2019/05:20 (13)	ND	3		
 	
	01051	Day 1	Yes					
		Day 2	No	05DEC2019/19:07 (1)	340.68	3		
		Day 3	Yes					
		Day 4	Yes					
		Day 5	Yes					
		Day 6	Yes					
		Day 7	Yes					
 	
	01059	Day 1	Yes					
		Day 2	No	05DEC2019/17:53 (1)	112.13	4		
				05DEC2019/19:12 (1)	242.16	4		
				05DEC2019/21:18 (1)	299.60	4		
		Day 3	No	06DEC2019/02:08 (2)	130.28	4		
				06DEC2019/09:58 (2)	290.72	4		
				06DEC2019/11:46 (2)	241.82	4		
				06DEC2019/14:31 (2)	396.00	4		
				06DEC2019/15:38 (2)	152.25	4		
				06DEC2019/19:46 (2)	347.29	4		
 	
Study day is relative to the initiation of study drug on Day 1.
Program: l_diar.sas 	Executed: 01DEC2020 16:53   	


PATH 
Protocol: DRG-032-PO-2-01-USA 	Page 25 of 36 	

Listing 16.2.6.1 Diarrhea Collection - Safety Population

Treatment
Group	Subject ID	Visit	None	Date and Time of
Collection (Study Day)	Volume of Diarrhea
Collection(ml)	Grade	Comments	
Placebo	01059	Day 3	No	06DEC2019/23:14 (2)	478.93	4		
		Day 4	No	07DEC2019/02:05 (3)	239.08	4		
				07DEC2019/02:41 (3)	210.55	4		
				07DEC2019/09:44 (3)	389.13	4		
				07DEC2019/11:54 (3)	193.39	4		
				07DEC2019/15:42 (3)	284.55	3		
				07DEC2019/19:50 (3)	320.62	3		
		Day 5	No	08DEC2019/09:44 (4)	661.20	3		
				08DEC2019/13:09 (4)	176.74	4		
				08DEC2019/21:45 (4)	156.68	4		
		Day 6	No	09DEC2019/01:58 (5)	428.14	3		
				09DEC2019/10:50 (5)	343.78	3		
				09DEC2019/12:26 (5)	102.14	3		
		Day 7	Yes					
 	
	01063	Day 1	Yes					
		Day 2	No	29JAN2020/05:06 (1)	540.40	3		
				29JAN2020/07:05 (1)	102.30	4		
				29JAN2020/08:23 (1)	153.81	5		
				29JAN2020/09:32 (1)	278.40	5		
				29JAN2020/10:17 (1)	242.34	5		
				29JAN2020/11:06 (1)	305.32	5		
				29JAN2020/11:39 (1)	173.44	4		
 	
Study day is relative to the initiation of study drug on Day 1.
Program: l_diar.sas 	Executed: 01DEC2020 16:53   	


PATH 
Protocol: DRG-032-PO-2-01-USA 	Page 26 of 36 	

Listing 16.2.6.1 Diarrhea Collection - Safety Population

Treatment
Group	Subject ID	Visit	None	Date and Time of
Collection (Study Day)	Volume of Diarrhea
Collection(ml)	Grade	Comments	
Placebo	01063	Day 2	No	29JAN2020/13:44 (1)	278.81	4		
				29JAN2020/14:59 (1)	54.90	4		
				29JAN2020/15:56 (1)	156.45	4		
				29JAN2020/16:57 (1)	303.50	4		
				29JAN2020/18:07 (1)	316.24	4		
				29JAN2020/19:21 (1)	199.96	4		
				29JAN2020/21:18 (1)	425.72	4		
				29JAN2020/22:43 (1)	147.50	4		
				29JAN2020/23:38 (1)	271.81	4		
		Day 3	No	30JAN2020/01:01 (2)	91.71	4		
				30JAN2020/05:04 (2)	610.73	4		
				30JAN2020/09:15 (2)	499.70	5		
				30JAN2020/11:05 (2)	293.50	4		
				30JAN2020/11:35 (2)	152.08	4		
				30JAN2020/12:00 (2)	185.35	4		
				30JAN2020/12:54 (2)	140.24	4		
				30JAN2020/14:15 (2)	326.74	5		
				30JAN2020/14:49 (2)	115.43	4		
				30JAN2020/16:17 (2)	222.43	5		
				30JAN2020/17:14 (2)	211.88	4		
				30JAN2020/17:44 (2)	114.76	4		
				30JAN2020/18:14 (2)	156.31	4		
 	
Study day is relative to the initiation of study drug on Day 1.
Program: l_diar.sas 	Executed: 01DEC2020 16:53   	


PATH 
Protocol: DRG-032-PO-2-01-USA 	Page 27 of 36 	

Listing 16.2.6.1 Diarrhea Collection - Safety Population

Treatment
Group	Subject ID	Visit	None	Date and Time of
Collection (Study Day)	Volume of Diarrhea
Collection(ml)	Grade	Comments	
Placebo	01063	Day 3	No	30JAN2020/19:34 (2)	227.97	4		
				30JAN2020/20:27 (2)	539.29	4		
				30JAN2020/21:34 (2)	274.02	5		
				30JAN2020/22:35 (2)	213.72	5		
				30JAN2020/23:31 (2)	234.28	5		
		Day 4	No	31JAN2020/04:45 (3)	441.83	4		
				31JAN2020/08:37 (3)	229.06	4		
				31JAN2020/10:21 (3)	282.44	4		
				31JAN2020/14:13 (3)	112.00	4		
				31JAN2020/16:47 (3)	117.90	4		
				31JAN2020/18:52 (3)	246.60	3		
				31JAN2020/20:47 (3)	301.30	4		
		Day 5	No	01FEB2020/04:44 (4)	244.94	4		
				01FEB2020/09:53 (4)	217.00	4		
				01FEB2020/14:02 (4)	207.61	4		
				01FEB2020/16:33 (4)	94.26	3		
				01FEB2020/21:25 (4)	252.55	4		
				01FEB2020/22:50 (4)	163.22	3		
		Day 6	No	02FEB2020/01:07 (5)	168	4		
				02FEB2020/10:53 (5)	195.52	3		
				02FEB2020/13:43 (5)	122.47	4		
				02FEB2020/16:09 (5)	99.06	3		
 	
Study day is relative to the initiation of study drug on Day 1.
Program: l_diar.sas 	Executed: 01DEC2020 16:53   	


PATH 
Protocol: DRG-032-PO-2-01-USA 	Page 28 of 36 	

Listing 16.2.6.1 Diarrhea Collection - Safety Population

Treatment
Group	Subject ID	Visit	None	Date and Time of
Collection (Study Day)	Volume of Diarrhea
Collection(ml)	Grade	Comments	
Placebo	01063	Day 6	No	02FEB2020/20:46 (5)	169	3		
		Day 7	No	03FEB2020/09:14 (6)	169.9	3		
				03FEB2020/15:08 (6)	192.60	3		
				03FEB2020/20:06 (6)	147.45	3		
 	
	01068	Day 1	Yes					
		Day 2	No	29JAN2020/11:32 (1)	116.92	3		
				29JAN2020/12:50 (1)	197.75	4		
				29JAN2020/16:17 (1)	267.02	5		
		Day 3	No	30JAN2020/00:30 (2)	195.02	4		
				30JAN2020/10:34 (2)	96.37	5		
				30JAN2020/20:46 (2)	136.50	4		
		Day 4	No	31JAN2020/09:02 (3)	168.14	5		
				31JAN2020/20:34 (3)	115.27	4		
		Day 5	No	01FEB2020/14:58 (4)	151.03	4		
		Day 6	Yes					
		Day 7	Yes					
 	
	01072	Day 1	Yes					
		Day 2	No	29JAN2020/04:54 (1)	128.78	3		
				29JAN2020/05:32 (1)	258.86	3		
				29JAN2020/07:24 (1)	374.20	3		
				29JAN2020/09:06 (1)	130.75	4		
				29JAN2020/12:04 (1)	107.65	4		
 	
Study day is relative to the initiation of study drug on Day 1.
Program: l_diar.sas 	Executed: 01DEC2020 16:53   	


PATH 
Protocol: DRG-032-PO-2-01-USA 	Page 29 of 36 	

Listing 16.2.6.1 Diarrhea Collection - Safety Population

Treatment
Group	Subject ID	Visit	None	Date and Time of
Collection (Study Day)	Volume of Diarrhea
Collection(ml)	Grade	Comments	
Placebo	01072	Day 2	No	29JAN2020/14:17 (1)	216.93	5		
				29JAN2020/14:55 (1)	185.02	5		
				29JAN2020/15:31 (1)	184.54	5		
				29JAN2020/16:34 (1)	195.60	5		
				29JAN2020/18:42 (1)	195.60	5		
				29JAN2020/21:23 (1)	254.87	5		
		Day 3	No	30JAN2020/01:50 (2)	524.60	5		
				30JAN2020/04:11 (2)	408.24	5		
				30JAN2020/08:33 (2)	393.04	5		
				30JAN2020/10:03 (2)	456.36	5		
				30JAN2020/10:51 (2)	112.89	5		
				30JAN2020/12:02 (2)	328.60	5		
				30JAN2020/14:30 (2)	147.00	5		
				30JAN2020/15:55 (2)	344.93	5		
				30JAN2020/18:25 (2)	154.13	5		
				30JAN2020/20:35 (2)	352.10	5		
		Day 4	No	31JAN2020/01:17 (3)	285.38	5		
				31JAN2020/06:38 (3)	353.05	5		
				31JAN2020/08:45 (3)	180.20	5		
				31JAN2020/11:30 (3)	168.77	5		
				31JAN2020/15:02 (3)	218.54	5		
				31JAN2020/16:32 (3)	234.93	5		
 	
Study day is relative to the initiation of study drug on Day 1.
Program: l_diar.sas 	Executed: 01DEC2020 16:53   	


PATH 
Protocol: DRG-032-PO-2-01-USA 	Page 30 of 36 	

Listing 16.2.6.1 Diarrhea Collection - Safety Population

Treatment
Group	Subject ID	Visit	None	Date and Time of
Collection (Study Day)	Volume of Diarrhea
Collection(ml)	Grade	Comments	
Placebo	01072	Day 5	No	01FEB2020/05:47 (4)	298.55	5		
				01FEB2020/09:38 (4)	83.73	4		
				01FEB2020/23:06 (4)	151.58	3		
		Day 6	No	02FEB2020/06:50 (5)	226.49	3		
		Day 7	Yes					
 	
	01074	Day 1	Yes					
		Day 2	Yes					
		Day 3	No	30JAN2020/13:15 (1)	54.38	3		
				30JAN2020/15:45 (1)	50.21	3		
				30JAN2020/19:54 (1)	61.09	3		
		Day 4	No	31JAN2020/08:49 (2)	81.0	4		
				31JAN2020/12:58 (2)	66.57	4		
				31JAN2020/23:00 (2)	70.38	5		
		Day 5	No	01FEB2020/09:40 (3)	186.92	5		
				01FEB2020/20:30 (3)	83.45	4		
		Day 6	No	02FEB2020/10:41 (4)	69.56	3		
		Day 7	Yes					
 	
	01075	Day 1	Yes					
		Day 2	Yes					
		Day 3	Yes					
		Day 4	Yes					
		Day 5	Yes					
 	
Study day is relative to the initiation of study drug on Day 1.
Program: l_diar.sas 	Executed: 01DEC2020 16:53   	


PATH 
Protocol: DRG-032-PO-2-01-USA 	Page 31 of 36 	

Listing 16.2.6.1 Diarrhea Collection - Safety Population

Treatment
Group	Subject ID	Visit	None	Date and Time of
Collection (Study Day)	Volume of Diarrhea
Collection(ml)	Grade	Comments	
Placebo	01075	Day 6	Yes					
		Day 7	Yes					
 	
	01078	Day 1	Yes					
		Day 2	No	29JAN2020/14:13 (1)	214.70	3		
				29JAN2020/16:11 (1)	108.32	4		
				29JAN2020/18:04 (1)	114.80	4		
				29JAN2020/20:50 (1)	82.96	4		
		Day 3	No	30JAN2020/07:34 (2)	298.60	5		
				30JAN2020/09:25 (2)	188.97	5		
				30JAN2020/12:40 (2)	111.23	5		
				30JAN2020/14:45 (2)	156.81	5		
				30JAN2020/16:08 (2)	183.14	5		
		Day 4	No	31JAN2020/08:05 (3)	308.91	5		
				31JAN2020/16:11 (3)	374.27	5		
		Day 5	No	01FEB2020/09:59 (4)	462.86	4		
				01FEB2020/13:04 (4)	458.80	5		
				01FEB2020/16:37 (4)	325.84	4		
		Day 6	No	02FEB2020/08:53 (5)	189	4		
				02FEB2020/23:27 (5)	196.25	3		
		Day 7	No	03FEB2020/05:03 (6)	286.11	3		
				03FEB2020/18:15 (6)	202.70	3		
				03FEB2020/21:36 (6)	228.88	3		
 	
Study day is relative to the initiation of study drug on Day 1.
Program: l_diar.sas 	Executed: 01DEC2020 16:53   	


PATH 
Protocol: DRG-032-PO-2-01-USA 	Page 32 of 36 	

Listing 16.2.6.1 Diarrhea Collection - Safety Population

Treatment
Group	Subject ID	Visit	None	Date and Time of
Collection (Study Day)	Volume of Diarrhea
Collection(ml)	Grade	Comments	
Placebo	01080	Day 1	Yes					
		Day 2	No	05DEC2019/13:42 (1)	256.20	3		
				05DEC2019/15:01 (1)	173.00	4		
				05DEC2019/16:41 (1)	134.12	4		
				05DEC2019/18:06 (1)	177.80	5		
				05DEC2019/21:30 (1)	70.39	5		
				05DEC2019/23:48 (1)	141.31	4		
		Day 3	No	06DEC2019/09:52 (2)	204.68	4		
				06DEC2019/10:57 (2)	164.73	4		
				06DEC2019/15:03 (2)	214.38	4		
				06DEC2019/17:37 (2)	303.54	5		
				06DEC2019/21:27 (2)	123.12	3		
		Day 4	No	07DEC2019/10:05 (3)	302.90	5		
				07DEC2019/12:11 (3)	293.16	5		
				07DEC2019/13:14 (3)	209.04	4		
				07DEC2019/14:46 (3)	340.11	4		
				07DEC2019/18:20 (3)	340.43	5		
				07DEC2019/19:25 (3)	231.20	4	Red spots in stool	
				07DEC2019/20:20 (3)	152.48	4		
				07DEC2019/22:18 (3)	317.24	4		
		Day 5	No	08DEC2019/14:51 (4)	254.12	5		
				08DEC2019/21:56 (4)	99.30	3		
 	
Study day is relative to the initiation of study drug on Day 1.
Program: l_diar.sas 	Executed: 01DEC2020 16:53   	


PATH 
Protocol: DRG-032-PO-2-01-USA 	Page 33 of 36 	

Listing 16.2.6.1 Diarrhea Collection - Safety Population

Treatment
Group	Subject ID	Visit	None	Date and Time of
Collection (Study Day)	Volume of Diarrhea
Collection(ml)	Grade	Comments	
Placebo	01080	Day 6	No	09DEC2019/10:31 (5)	53.6	4		
		Day 7	Yes					
 	
	01086	Day 1	Yes					
		Day 2	Yes					
		Day 3	No	30JAN2020/09:52 (1)	175.56	4		
				30JAN2020/11:22 (1)	122.36	4		
				30JAN2020/14:42 (1)	106.00	4		
				30JAN2020/23:33 (1)	231.85	3		
		Day 4	No	31JAN2020/15:52 (2)	146.55	3		
				31JAN2020/19:52 (2)	132.91	3		
		Day 5	No	01FEB2020/10:18 (3)	446.11	4		
				01FEB2020/12:23 (3)	137.23	4		
				01FEB2020/14:50 (3)	234.30	4		
		Day 6	No	02FEB2020/02:29 (4)	112.80	4		
				02FEB2020/09:35 (4)	310.91	4		
		Day 7	Yes					
 	
	01090	Day 1	Yes					
		Day 2	No	29JAN2020/18:55 (1)	215.42	3		
		Day 3	No	30JAN2020/01:20 (2)	470.95	4		
				30JAN2020/07:14 (2)	342.83	4		
				30JAN2020/19:08 (2)	335.31	4		
		Day 4	No	31JAN2020/09:44 (3)	617.35	4		
 	
Study day is relative to the initiation of study drug on Day 1.
Program: l_diar.sas 	Executed: 01DEC2020 16:53   	


PATH 
Protocol: DRG-032-PO-2-01-USA 	Page 34 of 36 	

Listing 16.2.6.1 Diarrhea Collection - Safety Population

Treatment
Group	Subject ID	Visit	None	Date and Time of
Collection (Study Day)	Volume of Diarrhea
Collection(ml)	Grade	Comments	
Placebo	01090	Day 4	No	31JAN2020/15:04 (3)	256.64	4		
				31JAN2020/20:03 (3)	226.60	4		
		Day 5	No	01FEB2020/08:14 (4)	671.86	4		
				01FEB2020/20:15 (4)	620.90	4		
		Day 6	No	02FEB2020/07:24 (5)	624.90	4		
				02FEB2020/20:05 (5)	166.83	4		
		Day 7	Yes					
 	
	01092	Day 1	Yes					
		Day 2	Yes					
		Day 3	No	30JAN2020/01:38 (1)	319.15	4		
				30JAN2020/11:33 (1)	166.27	4		
		Day 4	Yes					
		Day 5	No	01FEB2020/02:26 (3)	187.65	4		
				01FEB2020/06:32 (3)	490.17	4		
				01FEB2020/07:56 (3)	291.32	4		
				01FEB2020/09:46 (3)	377.08	5		
				01FEB2020/11:07 (3)	98.72	5		
				01FEB2020/15:21 (3)	175.46	5		
				01FEB2020/17:45 (3)	329.91	5		
				01FEB2020/18:46 (3)	132.03	5		
				01FEB2020/23:56 (3)	232.12	5		
		Day 6	No	02FEB2020/02:02 (4)	272.75	5		
 	
Study day is relative to the initiation of study drug on Day 1.
Program: l_diar.sas 	Executed: 01DEC2020 16:53   	


PATH 
Protocol: DRG-032-PO-2-01-USA 	Page 35 of 36 	

Listing 16.2.6.1 Diarrhea Collection - Safety Population

Treatment
Group	Subject ID	Visit	None	Date and Time of
Collection (Study Day)	Volume of Diarrhea
Collection(ml)	Grade	Comments	
Placebo	01092	Day 6	No	02FEB2020/06:55 (4)	148.29	5		
				02FEB2020/09:01 (4)	214.23	5		
				02FEB2020/11:38 (4)	119.93	3		
		Day 7	Yes					
 	
	01100	Day 1	Yes					
		Day 2	Yes					
		Day 3	Yes					
		Day 4	No	31JAN2020/10:30 (2)	83.69	4		
				31JAN2020/22:50 (2)	86.43	3		
		Day 5	No	01FEB2020/14:34 (3)	185.79	3		
				01FEB2020/16:26 (3)	175.80	3		
		Day 6	Yes					
		Day 7	Yes					
 	
	01102	Day 1	Yes					
		Day 2	No	29JAN2020/16:56 (1)	115.30	4		
				29JAN2020/18:10 (1)	120.80	4		
		Day 3	No	30JAN2020/01:30 (2)	125.90	3		
				30JAN2020/09:06 (2)	215.95	5		
				30JAN2020/19:02 (2)	50.65	5		
				30JAN2020/19:03 (2)	50.75	3		
		Day 4	No	31JAN2020/12:10 (3)	109.37	4		
				31JAN2020/23:02 (3)	100.52	3		
 	
Study day is relative to the initiation of study drug on Day 1.
Program: l_diar.sas 	Executed: 01DEC2020 16:53   	


PATH 
Protocol: DRG-032-PO-2-01-USA 	Page 36 of 36 	

Listing 16.2.6.1 Diarrhea Collection - Safety Population

Treatment
Group	Subject ID	Visit	None	Date and Time of
Collection (Study Day)	Volume of Diarrhea
Collection(ml)	Grade	Comments	
Placebo	01102	Day 5	No	01FEB2020/00:59 (4)	75.60	4		
				01FEB2020/04:38 (4)	72.45	3		
				01FEB2020/14:46 (4)	179.26	5		
				01FEB2020/16:52 (4)	102.13	5		
		Day 6	No	02FEB2020/01:33 (5)	152.11	3		
		Day 7	Yes					
 	
Study day is relative to the initiation of study drug on Day 1.
Program: l_diar.sas 	Executed: 01DEC2020 16:53   	
